# Active upper‐limb therapies for hand function, individual goal achievement, and self‐care in children with cerebral palsy: A network meta‐analysis

**DOI:** 10.1111/dmcn.16476

**Published:** 2025-09-05

**Authors:** Andrea Burgess, Mark D. Chatfield, Diana Hermith‐Ramirez, Michelle Jackman, Megan Thorley, Sarah Reedman, Roslyn N. Boyd, Leanne Sakzewski

**Affiliations:** ^1^ Queensland Cerebral Palsy and Rehabilitation Research Centre, Child Health Research Centre, Faculty of Medicine The University of Queensland Brisbane QLD Australia; ^2^ Children's Health Queensland Queensland Paediatric Rehabilitation Service Brisbane QLD Australia; ^3^ Cerebral Palsy Alliance Research Institute The University of Sydney Sydney NSW Australia

## Abstract

**Aim:**

To compare active upper‐limb therapies for children with cerebral palsy using a network meta‐analysis.

**Method:**

For this systematic review, five electronic databases were searched up to 2nd September 2024. Outcomes pertaining to improved hand use (Assisting Hand Assessment, AHA), goal attainment (Canadian Occupational Performance Measure, COPM), and self‐care were analysed with therapies classified into 15 discrete categories.

**Results:**

Quantitative analysis of 48 randomized controlled trials (*n* = 1629) was performed. Compared with control, treatment effect on hand function (AHA mean difference, standard error) was greater for bimanual therapy (BiM: 4.6, 1.0), modified constraint‐induced movement therapy (mCIMT; 4.0, 1.0), goal‐directed therapy (GDT; 3.8, 1.6), action observation (4.9, 1.1), and mCIMT + intensive (7.4, 2.5). For COPM performance, treatment effect was greater for cognitive orientation to occupational performance (CO‐OP; 5.9, 1.4), BiM (3.3, 0.4), mCIMT (2.5, 0.5), GDT (2.3, 0.7), mirror therapy (2.6, 1.2), and mCIMT + GDT (4.2, 1.1). For self‐care, treatment effect (standardized mean difference, standard error) was greater for BiM (0.39, 0.10), mCIMT (0.37, 0.08), and mCIMT + GDT (0.43, 0.32).

**Interpretation:**

BiM and mCIMT were confirmed as effective interventions for hand function, self‐care, and individual goal achievement. Mirror therapy, CO‐OP, and four different combination approaches feature single studies, small sample sizes, and high risk of bias, requiring further clinical trials to confirm efficacy.

## Graphical abstract

This systematic review examined effectiveness of upper limb functional therapies for children with cerebral palsy, where the child was an active participant. Modified Constraint Induced Movement Therapy (mCIMT) followed by Bimanual Therapy (BiM) comprised the largest number of studies and both approaches consistently demonstrated statistically significant effectiveness across all three outcomes of bimanual performance, individual goal achievement and self‐care.

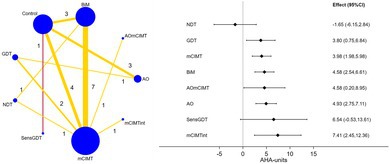

**What this paper adds**

- Bimanual therapy (BiM) and modified constraint‐induced movement therapy (mCIMT) are effective interventions for hand function, self‐care, and individual goal achievement.
- Action observation is confirmed as an effective intervention for hand function.
- Goal‐directed therapy and cognitive orientation to occupational performance are effective for individual goal achievement.
- Both BiM and mCIMT have a large treatment effect on hand function for children aged 0 to 3 years.

**Abbreviations:**
AHA: Assisting Hand Assessment
BiM: bimanual therapy
CIMT: constraint‐induced movement therapy
CO‐OP: cognitive orientation to daily occupational performance
COPM: Canadian Occupational Performance Measure
GDT: goal‐directed therapy
HABIT: hand arm bimanual intensive training
HEI: home‐based early intervention
mCIMT: modified constraint‐induced movement therapy
NDT: neurodevelopmental treatment
NMA: network meta‐analysis
PEDI: Pediatric Evaluation of Disability Inventory
PEDI‐CAT: Pediatric Evaluation of Disability Inventory ‐ Computer Adaptive Test
RCT: randomized controlled trial.

Cerebral palsy (CP)^1^ is the most common physical disability in childhood,^2^ with estimated prevalence in high‐income countries ranging from 1.4 to 1.8 per 1000 live births, and in low‐ and middle‐income countries from 3.2 to 3.7 per 1000 children.^3^, ^4^, ^5^, ^6^ Around 30% of children with CP have a unilateral presentation,^2^ while for children with bilateral CP the upper‐limb involvement may be symmetrical or asymmetrical.^7^, ^8^ Impaired upper‐limb function leads to limitations in independence, participation, and quality of life. Effective rehabilitation for upper‐limb dysfunction is paramount to promote better performance of day‐to‐day bimanual activities and to achieve functional independence in home, school, and community life.

There is a large body of evidence to support upper‐limb rehabilitation approaches for children with unilateral CP compared with those for children with bilateral CP.^9^, ^10^ Findings suggest moderate to strong effects for intramuscular injections of botulinum neurotoxin A combined with occupational therapy to improve upper‐limb outcomes and achieve individualized goals for children with unilateral CP.^10^ Bimanual therapy (BiM) and modified constraint‐induced movement therapy (mCIMT) provide modest to strong treatment effects on upper‐limb movement quality and efficiency compared with usual care, although there is some debate about dosage required.^10^, ^11^, ^12^ Evidence supports the use of occupational therapy home programmes to provide a ‘dose boost’ or alternative method of delivery for evidence‐based interventions.^10^ A systematic review of action observation training suggests that action observation is a promising intervention for upper‐limb rehabilitation; however, a meta‐analysis was not performed.^13^ Mirror therapy was found to have a strong treatment effect in a review; however, it is noted only one study involving the intervention was included.^14^ A recent systematic review of interventions for children with bilateral CP identified those such as hand arm bimanual intensive training (HABIT), HABIT including lower extremity, goal‐directed therapy (GDT), and task‐specific training; however, the review did not perform a meta‐analysis.^9^ Given the expansion of knowledge in this area, it is timely to perform a systematic review of upper‐limb therapies with appropriate analyses to compare therapies and summarize the current evidence. Owing to the large increase in studies, this systematic review focused on functional therapy approaches^15^ where the child is an active participant, on the basis of International Clinical Practice Guidelines^16^ and current theoretical frameworks.^17^, ^18^, ^19^

The aim of this review was to determine the efficacy of all non‐surgical, active, functional upper‐limb therapies for children and young people with CP, with a focus on upper‐limb performance, achievement of individualized goals, and self‐care skills. Therapy approaches were classified as functional or non‐functional in line with previously published definitions.^15^ Functional interventions involve age‐appropriate tasks and movements that aim to improve functional goal performance using task‐specific practice or practice of movements that contribute to the goal.^15^ This review included children with unilateral and bilateral CP, with the aim of enabling a better understanding of effective upper‐limb rehabilitation therapies that can be offered to all children with CP.

Owing to the numerous outcome measures used in studies, we chose to focus on those most frequently used. These were performance‐based^16^ measures with constructs of upper‐limb activity performance, self‐care skills, and achievement of individualized goals. There is a strong relation between self‐care and bimanual performance,^20^ and the measures chosen align with the aim of understanding the efficacy of upper‐limb therapies with a focus on activity performance.^16^

In the past decade, network meta‐analysis (NMA) has evolved as a method for comparing multiple interventions simultaneously in a single analysis.^21^ NMA provides information on the relative effects when there are three or more competing interventions by combining direct and indirect evidence across a network of randomized controlled trials (RCTs). The method is based on the simultaneous synthesis of direct evidence from comparison of RCTs of interest, as well as the indirect evidence derived from comparing studies of interest that have a common treatment comparator.^21^ The benefit of using NMA is that all interventions of interest may be included in an analysis.

## METHOD

This review was registered with PROSPERO (registration number CRD42020204880).

### Search strategy

Five databases were searched from inception until 2nd September 2024 (MEDLINE, CINAHL [Cumulative Index to Nursing and Allied Health Literature], Embase, Cochrane Central Register of Controlled Trials, and Web of Science). The search strategy from the previous systematic review^10^, ^22^ was revised, including Medical Subject Headings (MeSH) terms and key words focused on ‘cerebral palsy’ AND ‘therapy/intervention’ AND ‘upper limb’ AND ‘randomized controlled trial’ OR ‘quasi‐randomized trial’. The full search strategies are provided in Appendix S1. No exclusion restrictions based on language were applied.

### Study selection, inclusion criteria, and exclusion criteria

Two authors (LS, AB) independently assessed titles and abstracts against eligibility criteria. Inclusion criteria were: (1) the population comprised children 0 to 18 years with CP; (2) the study evaluated the efficacy of an activity‐based, functional, upper‐limb therapy (where interventions combined lower extremity or whole‐body training, more than 50% of the intervention needed to target the upper‐limbs); (3) the study was an RCT, and in addition quasi‐RCTs were included for children with bilateral CP; and (4) outcome measures assessed upper‐limb unimanual or bimanual capacity and/or performance, achievement of individualized goals, or self‐care skills. Articles were excluded if interventions were not specific to the upper‐limb (e.g. whole‐body interventions), involved the child in a passive role (e.g. surgery, botulinum neurotoxin A, stretching, positioning, splinting, taping, acupuncture), or were not functional in their approach (e.g. computer gaming, virtual reality), robot‐assisted interventions, or strengthening where the movements were not connected to real‐life functional activities. Adjunctive treatments used in combination with an activity‐based upper‐limb therapy were not included. Global activity measures unable to provide data specific to the upper‐limb were excluded. Conference abstracts were excluded. Outcome measures were excluded if they did not have published validity or reliability for children with CP or were invalidated owing to the adaptation of administration or scoring.

### Outcome measures

There were 24 outcome measures with evidence for validity and reliability. Eligible and ineligible tools are listed in Appendix S1. To overcome the presence of multiple outcome measurement tools, outcomes were chosen based on: (1) those that represented constructs of interest (i.e. upper‐limb performance, goal attainment, self‐care), (2) were valid, and (3) most frequently used. The two most common measures were used as primary outcomes: the Assisting Hand Assessment (AHA), which measures bimanual upper‐limb performance for children with unilateral CP, and the Canadian Occupational Performance Measure (COPM) performance scale, which measures functional goal achievement for children with bilateral and unilateral CP. A change of 5 AHA units (or 4 raw score points) is regarded as the smallest detectable change beyond measurement error on the AHA for children aged between 18 months and 18 years who have unilateral CP.^23^, ^24^ A change of more than 2 COPM units is commonly considered as indicative of real change,^25^ although this has not been empirically supported.^26^ Secondary outcome measures evaluating self‐care performance included the Pediatric Evaluation of Disability Inventory (PEDI) self‐care domain, PEDI ‐ Computer Adaptive Test (PEDI‐CAT) daily activities domain, the ABILHAND‐Kids, and the Functional Independence Measure for Children (WeeFIM).

The AHA, the COPM performance domain, and the PEDI, PEDI‐CAT, ABILHAND‐Kids, and Functional Independence Measure for Children represent different performance constructs of hand function, individual goal achievement, and self‐care respectively. Analysis of each construct was considered beneficial as different interventions may produce different treatment effects, according to the outcome measure that analyses are based upon. The AHA measures upper‐limb performance directly, while the COPM and self‐care measurement tools indirectly assess hand function (e.g. activity goals relating to upper‐limb function or self‐care activities).

### Data extraction

Structured data extraction forms were used by two authors (AB and LS) independently. Where data were missing, authors were contacted to request relevant information. Study methodology, number of participants, and intervention and control group details were extracted. Numerical data were cross checked. For studies where data were duplicated in more than one publication, the most recent and complete data set was included. Where data were not published, they were sourced from previously published systematic reviews or directly requested from the author.

### Risk of bias

Risk of bias of individual included studies was rated independently by at least two reviewers (AB, MT, MJ, SR, MDC, LS) using the revised Cochrane Risk of Bias 2.0 tool.^27^ Conflicts were resolved through discussion or a third reviewer (AB or LS).

### Grouping interventions

Two authors (AB and LS) allocated the various intervention types into categories. When consensus could not be reached, a third author (MT or MJ) supported the final decision. Category definitions were as follows: action observation, BiM, mCIMT, cognitive orientation to daily occupational performance (CO‐OP), GDT, home‐based early intervention (HEI), mirror therapy, neurodevelopmental treatment (NDT), sensory stimulation reminder, sensory and GDT (sensory + GDT), mCIMT and intensive (mCIMT + intensive), mCIMT and GDT (mCIMT + GDT), action observation and mCIMT (action observation + mCIMT), HEI and sensory stimulation reminder (HEI + sensory stimulation reminder), and control group. Definitions of intervention group categories are found in Appendix S1. For example, the intervention of HABIT was allocated to the BiM category. Approaches such as GDT and CO‐OP, which focus on individualized goals and self‐care activities, indirectly influence manual abilities, whereas interventions such as mCIMT, BiM, and mirror therapy focus directly on improving hand function. These approaches all involve the child as an active participant and are complementary to one another.

### Statistical analyses

Continuous outcomes for each study were summarized using means and standard deviation. For AHA and COPM, results were analysed using change from baseline scores, which accounts for differences in baseline performance of intervention and control groups and provides greater precision in estimating effect sizes. When the mean and standard deviation of the change were not available, the post‐intervention mean and standard deviation were used except when there were clinically significant between‐group differences at baseline (e.g. >5 AHA units or >2 COPM units). Changes from baseline data were combined with post‐intervention measurement data in analysis as necessary.^28^ The analysis of self‐care used multiple measurement tools and/or different measurement scales, so standardized mean differences were calculated.^28^ We considered a standardized mean difference of 0.2 as small, 0.5 moderate, and 0.8 large.^29^ The 95% confidence interval (CI) for the differences between two treatment categories was interpreted as a plausible range of the average treatment effect.^30^ We followed recommendations in the Cochrane Handbook for Systematic Reviews of Interventions^31^ for cross‐over trials and cluster RCTs. Data from all arms of multi‐arm trials were included as appropriate. Dosage of interventions and whether interventions and control/comparators were matched in dosage was recorded. It should be noted that dosage comparison studies cannot be included in NMA. Studies that had clinically significant between‐group differences at baseline for which the mean and standard deviation of change were not available were not included. If a study reported more than one measure of self‐care, priority was given to the PEDI and PEDI‐CAT for the NMA. Data pertaining to interventions for the subgroup of infants and toddlers (<3 years) were inspected.

#### Statistical methods for network meta‐analyses

Three separate sets of NMAs were conducted: (1) bimanual upper‐limb performance for children with unilateral CP; (2) functional goal achievement; and (3) self‐care. For NMA to be valid, assumptions of transitivity and consistency must be examined.^21^, ^32^ The review authors (who were content area experts) judged the clinical characteristics of the population in the included studies were similar (children with CP, predominantly Manual Ability Classification System levels I–III). To assess consistency, statistical assessment of disagreement between direct and indirect evidence was performed using local and global approaches.^33^ If inconsistency was observed, the presence of effect modifiers influencing treatment effect was considered. Known effect modifiers such as dosage of intervention and risk of bias were considered, as well as age of participants. Sensitivity analyses were performed using: (1) only studies that had matched intervention dosage (all levels of risk of bias), (2) only studies categorized as having ‘low’ or ‘some’ risk of bias (studies with ‘high’ risk of bias were excluded), and (3) only studies with ‘low’ or ‘some’ risk of bias and matched dosage. A summary of findings for each of the three outcomes is presented, guided by the sensitivity analyses wherein the analysis that yielded the highest number of studies while maintaining ‘consistency’ was used. Additional analyses for the outcome relating to achievement of individual goals (COPM) used a threshold of more or less than 25 hours of intervention,^15^ and studies that pertained to children under the age of 3 years were inspected. Analyses were performed in Stata version 18 (StatCorp, College Station, TX, USA) using a multivariate meta‐analysis approach.^33^

## RESULTS

### Search results and study characteristics

A total of 4908 records were screened (after removal of duplicates), 153 full‐text articles were assessed for eligibility, 101 studies were included in the qualitative analysis, and data from 48 trials investigating 15 categories of interventions were used in the quantitative analysis (*n* = 1629). The PRISMA flowchart is given in Figure S1. Study characteristics and methods of the included RCTs are summarized in Tables S1 and S2. Nine studies were classified as having ‘low risk of bias’, 36 as ‘some concerns’, and 22 as ‘high risk of bias’. Risk of bias ratings for each study used in quantitative analyses are given in Appendices S2 to S5. Risk of bias for all studies (including those not used in quantitative analyses) are in Appendix S6.

Thirty‐nine studies included children with unilateral CP only (*n* = 1330), three studies^34^, ^35^, ^36^ included children with bilateral CP only (*n* = 82), and six studies^37^, ^38^, ^39^, ^40^, ^41^, ^42^ included children with both unilateral and bilateral CP (*n* = 216). Ages of participants ranged from 6 months to 16 years, with most being older than 3 years. Five studies^43^, ^44^, ^45^, ^46^, ^47^ reported outcomes for children younger than 3 years. Adverse events, adherence, loss to follow‐up, and financial disclosure are provided in Table S3. Reasons for study or data exclusion are given in Table S4. Data from three studies were excluded from NMA because of baseline differences between intervention and control group which could have confounded treatment effects by reflecting pre‐existing differences rather than the true impact of the intervention (Appendix S4).

Therapy approaches included action observation (five trials, *n* = 74), action observation + GDT (one trial, *n* = 15), action observation + mCIMT (one trial, *n* = 22), BiM (19 trials, *n* = 347), mCIMT (41 trials, *n* = 694), mCIMT + intensive (one trial, *n* = 25), CO‐OP (two trials, *n* = 15), GDT (10 trials, *n* = 181), HEI (one trial, *n* = 8), HEI sensory stimulation reminder (one trial, *n* = 8), mirror (six trials, *n* = 99), NDT (four trials, *n* = 99), sensory + GDT (one trial, *n* = 7), and control (41 trials, *n* = 632).

### NMA results

**FIGURE 1:**
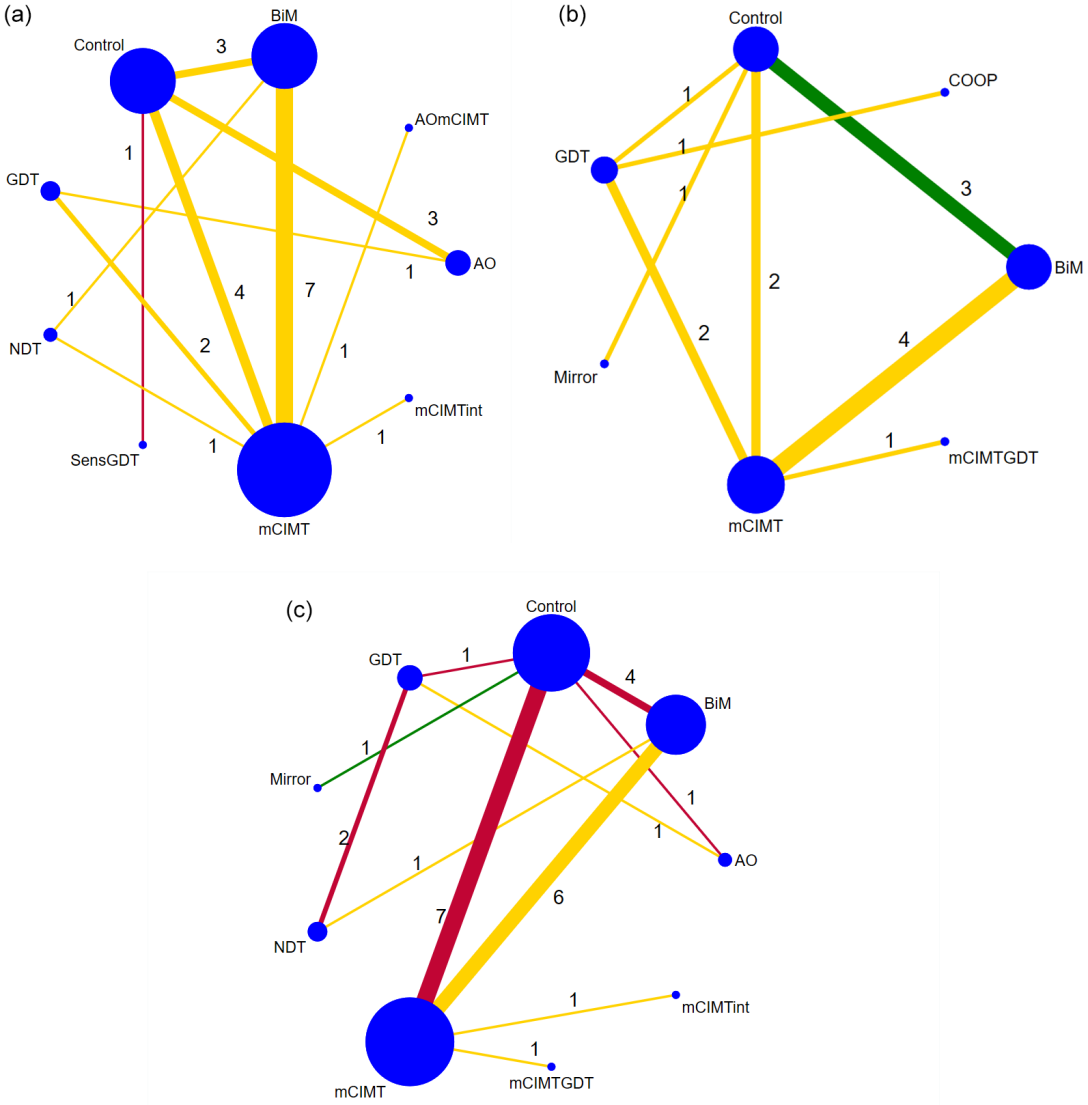
Network for (a) bimanual upper‐limb performance for children with unilateral CP using AHA data, (b) functional goal achievement for children with bilateral and unilateral CP using the COPM performance scale, and (c) self‐care (measurement tools: PEDI self‐care domain, PEDI‐CAT, ABILHAND‐Kids, and the Functional Independence Measure for Children [WeeFIM]). Node (circle) size indicates the number of studies reporting each intervention (larger circle indicates more studies), the width of lines is proportional to the number of trials comparing every pair of interventions, and the colour of lines indicates the most prevalent bias level (low, green; some concerns, yellow; high, red) for each direct comparison (with the worst category chosen when there was a tie). Total numbers of studies for each direct comparison are notated. Density (measure of connectedness) of each network graph was sparse (*a* = 0.36, *b* = 0.35, *c* = 0.31). Note for NMA of COPM performance that studies classified as being at high risk of bias were excluded. Abbreviations: AHA, Assisting Hand Assessment; AO, action observation; BiM, bimanual therapy; COPM, Canadian Occupational Performance Measure; CP, cerebral palsy; GDT, goal‐directed therapy; mCIMT, modified constraint‐induced movement therapy; mCIMTint, modified constraint‐induced movement therapy and intensive; NDT, neurodevelopmental treatment; NMA, network meta‐analysis; PEDI, Pediatric Evaluation of Disability Inventory; PEDI‐CAT, Pediatric Evaluation of Disability Inventory ‐ Computer Adaptive Test; SensGDT, sensory and goal‐directed therapy.

The network of comparisons for the AHA, COPM, and self‐care measures are presented in Figure 1. They show which interventions are involved and how many trials compare two particular interventions. Density (measure of connectedness) of each network graph was sparse (*a* = 0.36, *b* = 0.35, *c* = 0.31). A summary for each outcome was performed because conflict could arise for which interventions work best according to the outcome measure that the NMA is based upon. All sensitivity analyses are reported in Appendices S2 to S5.

### Primary outcomes

#### Bimanual upper‐limb performance: AHA

**FIGURE 2:**
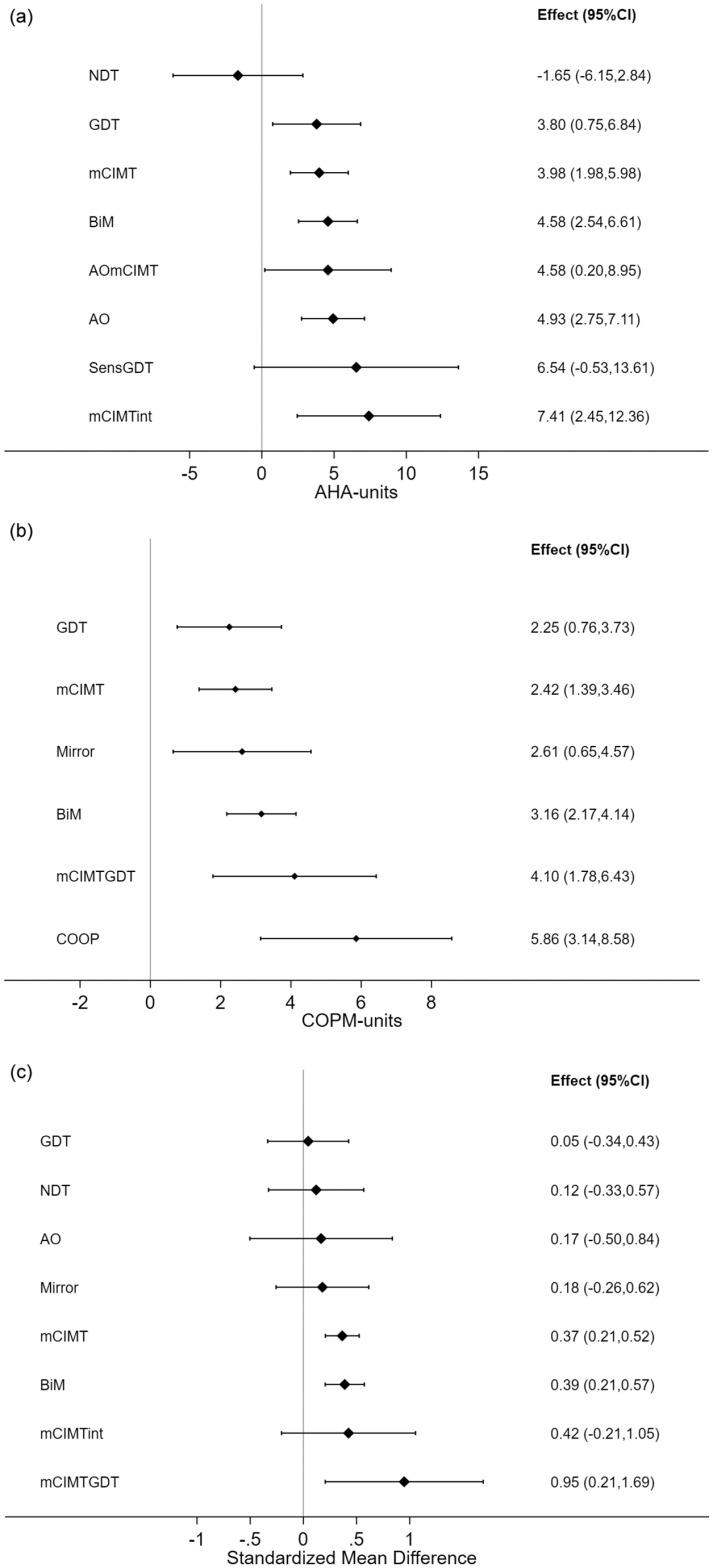
Efficacy of interventions for (a) bimanual upper‐limb performance for children with unilateral CP using AHA data, (b) functional goal achievement for children with bilateral and unilateral CP using the COPM performance scale, and (c) self‐care for children with bilateral and unilateral CP using standardized mean differences. Plots show treatment effect estimates for each intervention compared with control. Note for NMA of COPM performance that studies classified as being at high risk of bias were excluded. Abbreviations: AHA, Assisting Hand Assessment; AO, action observation; BiM, bimanual therapy; COPM, Canadian Occupational Performance Measure; CP, cerebral palsy; GDT, goal‐directed therapy; mCIMT, modified constraint‐induced movement therapy; mCIMTint, modified constraint‐induced movement therapy and intensive; NDT, neurodevelopmental treatment; NMA, network meta‐analysis; SensGDT, sensory and goal‐directed therapy.

Summary data for 25 studies reporting AHA data are provided in Appendix S2. There was no evidence of inconsistency between direct and indirect evidence in the treatment network when all studies were used in the analysis (*p* = 0.91) (Figure S2.1.2 in Appendix S2). Risk of bias was classified as ‘high’ for five studies, ‘low’ for five, and of ‘some concern’ for 15 (Table S2.1 in Appendix S2). There were 15 trials with matched dosage. There were eight intervention categories and one control category in the network comparison: mCIMT (16 trials, *n* = 305), BiM (11 trials, *n* = 194), action observation (four trials, *n* = 59), GDT (three trials, *n* = 74), NDT (two trials, *n* = 19), action observation + mCIMT (one trial, *n* = 22), sensory + GDT (one trial, *n* = 7), mCIMT + intensive (one trial, *n* = 25), and control (11 trials, *n* = 160). Compared with the control group, we estimated the average effect (mean difference in AHA units, standard error, 95% CI) was greater for BiM (4.6, 1.02, 2.5–6.6), action observation (4.9, 1.1, 2.8–7.1), mCIMT (4.0, 1.0, 2.0–6.0), and GDT (3.8, 1.6, 0.8–6.8) (Figure 2a and Figure S2.1.3 in Appendix S2). Compared with the control group, the estimated average treatment effect was greater for combinations of interventions: action observation + mCIMT (4.6, 2.2, 0.2–9.0), sensory + GDT (6.5, 3.6, −0.5 to 13.6), and mCIMT + intensive (7.4, 2.5, 2.5–12.4). The treatment effect for NDT was 1.7 AHA units (standard error = 2.3, 95% CI −6.2 to 2.8) less than control (Figure 2a and Table S2.1.1 in Appendix S2). Direct comparison of intervention categories shows there is no statistical difference between BiM and mCIMT, or BiM and action observation (Figure S2.1.3 in Appendix S2). Estimated probabilities of each intervention being the best and other ranking (the surface under the cumulative ranking curve) are presented in Figure S2.1.4 and Table S2.1.2 in Appendix S2.

#### Achievement of individualized goals: COPM performance

There were 21 studies available for quantitative analyses (Appendix S3). Sensitivity analysis of the COPM performance indicated that removing studies at high risk of bias provided larger and clinically meaningful differences in COPM performance between interventions and control groups. There was no evidence of inconsistency between direct and indirect evidence in the treatment network when studies with high risk of bias were removed (*p* = 0.172, Figure S3.3.2 in Appendix S3). Fifteen studies were included, six of which included children with bilateral CP.^34^, ^35^, ^36^, ^37^, ^41^, ^42^ Six intervention categories and the control were included: mCIMT (nine trials, *n* = 197), BiM (seven trials, *n* = 143), CO‐OP (one trial, *n* = 6), mirror (one trial, *n* = 15), GDT (four trials, *n* = 59), mCIMT + GDT (one trial, *n* = 10), and control (seven trials, *n* = 108) (Figure 1 and Appendix S3). Children who received CO‐OP performed on average 5.9 COPM units (standard error = 1.3, 95% CI 3.1–8.6) greater than control. Children who received a combination of mCIMT and GDT performed on average 4.2 COPM units (standard error = 1.1, 95% CI 2.0–6.4) greater than control. Compared with control, we estimated the average effect (mean difference in COPM units, standard error, 95% CI) was greater than 2 COPM units for all other interventions in the network: BiM (3.3, 0.4, 2.5–4.2), mCIMT (2.5, 0.5, 1.6–3.5), GDT (2.3, 0.7, 0.9–3.75), and mirror (2.6, 1.0, 0.7–4.5) (Figure 2 and Table S3.3.1 in Appendix S3). Estimated effect sizes and their uncertainties for all pairwise comparisons are provided (Figure 2b and Figure S3.3.3 in Appendix S3). Estimated probabilities of being the best and the surface under the cumulative ranking curve are shown in Figure S3.3.4 and Table S3.3.1 in Appendix S3.

There were eight trials involving three interventions which incorporated a matched dosage of more than 25 hours. All three interventions, GDT, BiM, and mCIMT, showed a positive mean difference (standard error) of 2.6 (0.8), 2.4 (0.4), and 1.9 (0.4) COPM units compared with control (Table S3.5.1 in Appendix S3).

### Secondary outcome

#### Self‐care outcomes

Twenty‐six studies were included in the quantitative analyses (Appendix S4). There was no evidence of inconsistency between direct and indirect evidence in the treatment network when all studies were included (*p* = 0.46; Figure S4.1.2 in Appendix S4). Risk of bias was classified as ‘high’ for nine studies, ‘some concern’ for 13, and ‘low’ for four. Three studies contained more than one self‐care outcome measure; the PEDI self‐care data were preferentially used in the analyses.^34^, ^48^, ^49^ Five of the 26 studies contained children with bilateral CP.^34^, ^35^, ^38^, ^39^, ^42^ There were 15 trials with matched dosage. Eight intervention categories and a control category were included: mCIMT (15 trials, *n* = 279), BiM (11 trials, *n* = 198), action observation (two trials, *n* = 23), GDT (four trials, *n* = 93), mirror (one trial, *n* = 38), NDT (three trials, *n* = 92), mCIMT + GDT (one trial, *n* = 10), mCIMT + intensive (one trial, *n* = 25), and control (14 trials, *n* = 247) (Table S4.1 in Appendix S4). Compared with control, the treatment effect as a standardized mean difference (standard error, 95% CI) was 0.95 (0.32, 0.21–1.69) for children who received mCIMT + GDT, 0.39 (0.09, 0.21–0.57) for children who received BiM, and 0.37 (0.08, 0.21–0.52) for children who received mCIMT (Figure 2c). The 95% CIs of mCIMT + intensive (−0.21 to 1.05), action observation (−0.5 to 0.84), mirror therapy (−0.26 to 0.62), GDT (−0.34 to 0.43), and NDT (−0.33 to 0.57) indicated such variability that they were not statistically significant. The point estimates of NDT (0.12), GDT (0.05), action observation (0.17), and mirror therapy (0.18) suggest negligible effect compared with control; however, the variation in the estimates prevents any conclusion (Table S4.1.1 in Appendix S4 and Figure 2c). Direct comparison showed there was no significant difference between the effects of BiM and mCIMT (Table S4.1.1 and Figure S4.1.3 in Appendix S4). Estimated probabilities of being the best and the surface under the cumulative ranking curve are shown in Appendix S4.

### Studies involving young children

There were four early intervention studies for children aged 0 to 3 years; all involved children with unilateral CP.^44^, ^45^, ^47^, ^50^ A fifth study included and reported separate results for children younger than 3 years.^43^ The changes and standard deviations for the studies involving infants are summarized in Appendix S5. NMA was performed on the subgroup of studies that measured the effects of mCIMT (two trials, *n* = 35) and BiM (two trials, *n* = 28) compared with control (two trials, *n* = 25) on hand function (AHA, mini‐AHA, HAI).^43^, ^44^, ^45^ Compared with control, both BiM and mCIMT had a large treatment effect (standardized mean difference, standard error) (0.88, 0.24; and 0.78, 0.30 respectively). One study could not be included as the outcome measure was too dissimilar, and the study involving HEI and HEI + sensory stimulation reminder could not be included in the NMA because of lack of common comparators.

## DISCUSSION

This systematic review examined the effectiveness of upper‐limb functional therapies for children with CP, where the child was an active participant. Modified CIMT followed by BiM comprised the largest number of studies and both approaches consistently demonstrated statistically significant effectiveness across all three outcomes (AHA, COPM performance, and self‐care measures). Action observation and GDT were found to improve bimanual hand function for children with unilateral CP. Mirror, CO‐OP, and interventions that combined different upper‐limb approaches (mCIMT + GDT, mCIMT + intensive, sensory + GDT) often yielded large effect sizes across outcomes; however, these results should be viewed with caution as they were determined from single studies with small sample sizes and high risk of bias.

For bimanual hand performance, mCIMT, BiM, action observation, GDT, mCIMT + intensive, and action observation + mCIMT were effective interventions compared with control, which concurs and builds upon previous findings of systematic reviews.^9^, ^11^, ^12^, ^13^, ^51^, ^52^ It should be noted, however, that while the treatment effects were statistically significant, they may or may not be clinically relevant. Uniquely, we were able to show no clear hierarchy in efficacy between these interventions, meaning that any of these therapies would be useful in practice depending on the child, family, and clinical preferences. Our findings suggest that NDT has similar efficacy as control, which concurs with previous systematic reviews.^53^ Similarly, the results for sensory and GDT imply that it can range from having no effect to a large effect. Our results differ from those of a systematic review and NMA published in 2022 which found that mirror therapy was the best intervention to improve upper‐limb function.^14^ The primary reason for this difference is likely to be that there were many RCTs included in our analyses which were not included in the 2022 review (e.g. 17 extra studies with AHA data included in our review). Other methodological differences include our use of change data and Cochrane methods for analysis of cross‐over trials.

The greatest gains in achievement of individual goals (COPM performance) for children with unilateral and bilateral CP resulted from CO‐OP, mCIMT + GDT, BiM, mirror, mCIMT, and GDT. It should be noted that the data involving CO‐OP, mCIMT + GDT, and mirror which contributed to these results were from single studies that had small sample sizes and were at high risk of bias. Our results point to BiM, mCIMT, or GDT as the preferred approaches to improve achievement of individualized goals until further studies on CO‐OP, mirror, and mCIMT + GDT are performed.

There was a small to moderate effect for mCIMT and BiM to improve self‐care outcomes for children with unilateral and bilateral CP compared with control, and small to large effect for mCIMT + GDT. Action observation, GDT, mirror, mCIMT + intensive, and NDT showed large variation in self‐care outcomes and were not statistically significant. These modest results for self‐care may be a consequence of the large range of activities that self‐care measures evaluate. For example, the PEDI assesses items that cover eating, use of utensils, hygiene, dressing, and toileting. It is probably harder to effect change on a broad measure of self‐care than an individual activity goal. The large effect of mCIMT + GDT to improve self‐care requires caution as it comes from a single study.

This review highlighted the paucity of studies for children with bilateral CP^9^ compared with those with unilateral CP: only three quasi/RCTs focused solely on this group and six studies included a combination of children with both unilateral and bilateral CP. Only one study included an outcome measure to assess bimanual performance of children with bilateral CP.^35^ Similarly, there were few studies of upper‐limb therapy for children with CP that started under 3 years of age.^44^, ^45^, ^47^, ^50^ Studies that focused on young children examined mCIMT, BiM, and HEI and only included children with unilateral CP. The lack of consistent outcome measurement tools across these studies precluded meta‐analysis with all the studies. Summary of three of the studies showed BiM and mCIMT were both effective in improving hand function in children under 3 years of age. HEI and HEI + sensory stimulation reminder were also shown to be effective interventions for young children, but could not be included in the NMA.^50^

Summarizing data from RCTs is an essential step in the process of translating research into practice. Using NMA allows comparison of all possible pairs of interventions. Using measures of effect that can be readily understood by clinicians, such as the AHA unit or COPM performance unit, assists meaningful interpretation. Reviews of interventions should not only ask whether an intervention is statistically significant but also if an intervention has a clinically worthwhile effect. Presenting results for hand function, goal achievement, and self‐care provides a more informed understanding of the different intervention effects captured by different outcome measurement constructs.

Several challenges were faced in this review. Studies varied in descriptive detail of the interventions and the authors used their best judgement to assign interventions to a category. The grouping of interventions together was necessary for performing NMA. Grouping of similar interventions together may, however, dilute the effects of an intervention. Dosage studies, where treatment groups received the same intervention but at different doses, were excluded from analyses. Similarly, studies that examined the effect of the environment (home vs clinic) on the same treatment approach were excluded. A limitation is that while valid outcomes were specified a priori, the vast number of outcome measures (24 measures, 55 different scales) rendered it impractical to use all of them. Thus, the most common and meaningful outcome measures were chosen a posteriori.

Caution is required in generalizing findings about interventions supported by only a single study as was the case with sensory + GDT, mCIMT + GDT, mCIMT + intensive, and CO‐OP. Effect estimates are a combination of direct and indirect comparisons. Indirect comparisons are required to estimate the relative effect of two interventions when no trial has compared them directly. For those interventions for which there is only one study, effect estimates are more dependent on indirect comparisons.

Both mCIMT and BiM consistently demonstrated statistically significant effectiveness for the three outcomes examined in this review (improved hand use for children with unilateral CP, and achievement of individual goals and self‐care for children with unilateral and bilateral CP). If focused on improving hand use (in children with unilateral CP), clinicians may confidently choose between mCIMT, BiM, action observation, and GDT, fitting in with the child, family, and clinical preferences.

We recommend development of an agreed set of standardized outcome measurement tools, or core outcome sets, to ensure that all trials provide usable information for reviews and meta‐analyses, allowing results to be compared and combined.^54^ For trials using continuous outcomes, we recommend reporting change from baseline^28^ as it gives a more precise estimate of treatment effect. We recommend further research for children younger than 3 years of age, those with bilateral CP, as well as those with more severe hand impairment (Manual Ability Classification System levels IV and V). Further research is also required on the interventions that showed large effects but for which there was only one trial. This review shows that no more studies are required to compare mCIMT and BiM with each other or with control for children with unilateral CP aged 3 years or older.

## CONCLUSION

This updated systematic review and network meta‐analyses confirmed mCIMT and BiM (including HABIT and HABIT including lower extremity) as effective interventions to improve bimanual hand performance, goal attainment, and self‐care. It confirmed that action observation also leads to improved bimanual hand performance but could not confirm the effectiveness of action observation on self‐care outcomes. Mirror therapy, CO‐OP, and the combination approaches mCIMT + GDT and mCIMT + intensive seem promising but require further clinical trials to confirm efficacy. This update confirms previous systematic review findings^9^, ^10^, ^11^, ^12^, ^53^ that there is flexibility in the choice of intervention approaches.

## FUNDING INFORMATION

This work was supported by the following: Leanne Sakzewski—National Health and Medical Research Council Career Development Fellowship (1160694) and a Children's Hospital Foundation Mary McConnel Career Boost for Women; Roslyn Boyd— National Health and Medical Research Council (NHMRC); Sarah Reedman—Medical Research Future Fund Early to Mid‐Career Researcher Grant (2022624); Andrea Burgess—University of Queensland Research Stimulus and Ramaciotti Health Investment Grant. The other authors received no additional funding.

## CONFLICT OF INTEREST STATEMENT

The authors have stated that they had no interests that might be perceived as posing a conflict or bias.

## Supporting information


**Table S1:** Study characteristics

**Table S2:** Structure/content of intervention programs

**Table S3:** Adverse events, adherence, loss to follow‐up

**Table S4:** Reason for exclusion from quantitative analysis

**Appendix S1:** Outcome measures and search strategy

**Appendix S2:** NMA and sensitivity analyses for AHA.

**Appendix S3:** NMA and sensitivity analyses for COPM performance.

**Appendix S4:** NMA and sensitivity analyses for Self‐care.

**Appendix S5:** Infant and Toddler studies.

**Appendix S6:** Risk of Bias for all studies.

**Figure S1:** PRISMA flowchart

## Data Availability

Data available on request from the authors.
