# Supplementary material for: Active upper‐limb therapies for hand function, individual goal achievement, and self‐care in children with cerebral palsy: A network meta‐analysis
Source: Dev Med Child Neurol. 2025 Sep 5;67(12):1543–53. doi: 10.1111/dmcn.16476 (PMC12618955; doi:10.1111/dmcn.16476)
Supplement: Supplementary file 2 — Table S2: Structure/content of intervention programs [file DMCN-67-1543-s003.docx]

## Table S2 Structure/Content of Intervention programs

| **Study** | **Content of intervention program** | **Duration**  **wks** | **Frequency**  **sessions/wk** | **Intensity**  **(hours)** | **Home Program (hr/day)** | **Content of control group** | **Duration**  **wks** | **Frequency**  **sessions/wk** | **Intensity**  **(hours)** | **Home Program (hr/day)** |  |
| --- | --- | --- | --- | --- | --- | --- | --- | --- | --- | --- | --- |
| ***Action Observation Training*** | | | | | | | | | | |  |
| Beani 2023[^22^](#_ENREF_22) | Action observation (video) | 3 | 5 |  | 1 | Standard care | 3 | NR | NR | NR |  |
| Buccino 2012 [^23^](#_ENREF_23) | Action observation + usual care | 3 | 5 | NR | NR | Sham video + usual care | 3 | 5 | NR | NR |  |
| Buccino 2018[^24^](#_ENREF_24) | Action observation + usual care | 3 | 5 | 0.5 | NR | Sham video + usual care | 3 | 5 | 0.5 | NR |  |
| Elbagoury 2022[^25^](#_ENREF_25) | Action observation + Goal Directed Training (functional training on specific ADL) | 4 | 3 | 0.5 | NR | Goal Directed Training (functional training on specific ADL) – verbal cues only | 4 | 3 | 0.5 | NR |  |
| Kim 2018[^26^](#_ENREF_26) | Action observation with live therapist demonstration: 3 min observation, 3 min instruction, 3 min repeated practice | 4 | 5 | 0.5 | NR | Action observation with videoed demonstration: 3 min observation, 3 min instruction, 3 min repeated practice | 4 | 5 | 0.5 | NR |  |
| Kim 2020[^27^](#_ENREF_27) | Action observation (3min video) + perform action (30 mins) with therapist verbal guidance | 4 | 3 | 0.5 | NR | Action observation (3min video) + perform action (60 mins) with therapist verbal guidance | 4 | 3 | 1.0 | NR |  |
| Kirkpatrick 2016[^28^](#_ENREF_28) | Action observation + repeated practice | 12 | 5 |  | 0.25 | Repeated practice | 12 | 5 |  | 0.25 |  |
| Palomo-Carrion 2023[^29^](#_ENREF_29) | Action observation (watch video then carry out observed activity for 4 mins), parent give verbal encouragement only | 4 | 5 |  | 1 | Action observation + Mirror therapy. (15m of mirror, then 45 m AOT same as AOT group) | 4 | 5 |  | 1 |  |
| Quadrelli 2019 [^30^](#_ENREF_30) | Action Observation (1 min video followed by 2 min practice x 6) used pre-set actions | 6 | 3 | 0.3 | NR | Watched videogame of cars. Children then asked to perform same actions as AO group. | 6 w | 3 | 0.3 | NR |  |
| Sgandurra 2013[^31^](#_ENREF_31) | Action observation | 3 | 5 | 1 | NR | Sham video | 3 | 5 | 1 | NR |  |
| Simon-Martinez, 2020 [^20^](#_ENREF_20) | CIMT + AO (9-day camp)  Restraint: hand splint  1 x 15hr AO (0.3 observation, 0.7 practice x 6 times).  9hr individual therapy: shaping, repetitive task practice.  30hr group activities: craft, outdoor play.  Total therapy=54h | 1.3 | 6-7 | 6 (1 individual, 2 AO, 3 group activity) | NR | CIMT + placebo  (9-day camp).  Restraint: hand splint  1 x 15hr (0.3 videogames no actions, 0.7 task practice)  9hr individual therapy  30h group activities  Total therapy=54hr | 1.3 | 6-7 | 6 (1 individual, 2 AO, 3 group activity) | NR |  |
| ***Bimanual, HABIT, HABIT-ILE*** | | | | | | | | | | |  |
| Araneda 2022[^4^](#_ENREF_4) | HABIT-ILE:Context: 2-week camp. Content: goal directed, bimanual activities with concomitant lower extremity and postural challenges | 2 | 5 | 9 | NR | Control: PT based on NDT or OT (1-5 h/wk) | NR | NR | NR | NR |  |
| Araneda 2024[^32^](#_ENREF_32) | HABIT-ILE:Context: 2-week camp. Content: goal directed, bimanual activities with concomitant lower extremity and postural challenges | 2 | 5 | 5 | NR | Control: Usual therapy and activities | 2 | 3 (PT/OT/Psych) | 0.75 | NR |  |
| Bleyenheuft 2015[^33^](#_ENREF_33) | HABIT-ILE: Context: groups at sleep over camp. Content: goal directed, bimanual activities with concomitant lower extremity and postural challenges | 2 | 5 | 9 | NR | Waitlist usual care (NDT) (mean total=80hr) | 20 | 1 | 1-5 | NR |  |
| Bleyenheuft 2017[^5^](#_ENREF_5) | HABIT-ILE: Context: groups 4-6. Content: age appropriate fine and gross motor bimanual activities, active problem solving, whole and part task practice, goal training | 2 | 6-7 (13 d total) | 6.5 | NR | Usual care: NDT | 2 | 1 | NR | NR |  |
| Brandao 2014[^34^](#_ENREF_34) | HABIT; Context: groups of 6-8. Content: Structured practice; age appropriate fine and gross motor bimanual activities, active problem solving, whole and part task practice, goal training (30 min/d). | 3 | 5 | 6 | NR | Unstructured bimanual practice: activities requiring two hands, no progression of complexity | 3 | 5 | 6 | NR |  |
| Brandao 2018[^35^](#_ENREF_35) | HABIT high dose: Context groups. Content: age appropriate fine and gross motor bimanual activities, active problem solving, whole and part task practice, goal training | 3 | 5 | 6 | 1 | HABIT low dose: Context groups. Training: age appropriate fine and gross motor bimanual activities, active problem solving, whole and part task practice, goal training | 1.5 | 5 | 6 | 1 |  |
| Facchin et al [^36^](#_ENREF_36) & Fedrizzi [^37^](#_ENREF_37)(a) *(see CIMT for other trial arms) | Bimanual training. Context: home, clinic; Content: bimanual play and ADL activities | 10 | 3 | 3 | 3 x 4d/wk | Usual care. Context: NR; Training: NR | 10 | 1-2 | 1 | NR |  |
| Ferre 2017[^38^](#_ENREF_38) | HABIT; Context: home. Content: bimanual tasks for reach, grasp, release and manipulation. Monitored via webcam by therapist. Supervision 1 hr/wk | 9 | 1 | 1 | 2 x 5d/wk | Lower limb intensive functional training | 9 | 1 | 1 | 2 x 5d/wk |  |
| Figueiredo 2020[^3^](#_ENREF_3) | HABIT in small groups (in Brazil). | 3 | 5 | 6 | Parent instructed to engage child in bimanual activities in home setting | Customary care | 3 | 2 | 0.75 | Parents instructed to promote child independence at home without any specific home program. |  |
| Friel 2021[^100^](#_ENREF_100) | CIMT  Restraint: cotton sling  Context: clinic  Content: Unimanual activities targeting hand impairments | 3 | 5 | 6 | NR | HABIT  Context: clinic  Content: bimanual activities using part and whole task practice | 3 | 5 | 6 | NR |  |
| Gordon 2007[^2^](#_ENREF_2) | HABIT; Context: groups of 4. Content: bilateral fine motor, manipulative gross motor activities. | 2 | 5 | 6 | 1 during study  2 post treatment x 4wks | Control group – customary care. Control received HABIT treatment after participation in trial. | 2 | NR | NR | NR |  |
| Kuo 2016[^21^](#_ENREF_21) | BiT (HABIT) with tactile training.  Activities using tactile stimulating materials with vision occluded included exploration, identification, matching of objects | 3 | 5 HABIT  +  8 x 1 hr sessions tactile training (vision occluded) | 5.7 | NR | BiT (HABIT). Used same tactile materials but without tactile training. | 3 | 5 HABIT  +  8 x 1 hr sessions with same materials with full vision used in context of play/function. | 5.7 | NR |  |
| ***Constraint Induced Movement Therapy (CIMT)*** | | |  |  |  |  |  |  |  |  |  |
| Aarts 2010[^39^](#_ENREF_39) | hCIMT-BIT  Restraint: sling; Context: rehab centre in groups of 6; Content: mCIMT shaping & repetitive task practice, goal directed bimanual training | 8 (6 wks mCIMT,  2wks BIM) | 3 | 3 | Expected duration NR | Usual care. Context: rehab centres; Content: stretch, weight bearing, bimanual therapy | 8 | 2 | 1.5 | 11/wk |  |
| Abd El-Kafy 2014[^41^](#_ENREF_41) | mCIMT  Restraint: sling  Context: clinic and home  Content: repetitive task practice with shaping. Home practice: reaching, weight bearing, strengthening, manipulation, self-care | 4 | 5 | 4 | 2 x 5 d/wk | Conventional therapy  Context: clinic and home. Content: PT and OT not specified  Home program: as per mCIMT group | 4 | 5 | 4 | 2 x 5d/wk |  |
| Afzal 2022[^101^](#_ENREF_101) | CCIMT | 3 | 5 | 6 | NR | MCIMT | 2 | 5 | 2 | NR |  |
| Al Oraibi [^42^](#_ENREF_42) | mCIMT  Restraint: glove; Context: home and centre based; Content: fine motor tasks | 8 | 1 | NR | 2 x 6 d/wk | NDT: weight bearing, facilitation arm movement; Context, home | 8 | 1 | 1-2 | NR |  |
| Bingol 2022[^102^](#_ENREF_102) | mCIMT across multiple environments (school, home, clinic) using individual sessions at school, group session at clinic and home practice. (Total therapy =75h) | 10 | 3 | 2.5 | 1 during study; 2 in follow-up period (total home practice= 294 h) | BiT  Context: individual sessions at school, group session at clinic and home practice. (Total therapy=75h) | 10 | 3 | 2.5 | 1 during study; 2 in follow-up period (total home practice= 294 h) |  |
| De Brito Brandao 2010[^43^](#_ENREF_43) | hCIMT  Restraint: resting splint and sling; Context: clinic; Content: shaping of fine motor, ADLs | 3  (2 mCIMT  1 BIM) | 5  3 | 4  0.75 | Restraint: 10 | Usual care. Context: NR; Content: bimanual activities, sensory stimulation | 3 | 1 | 0.75 | NR |  |
| Case-Smith et al 2012[^44^](#_ENREF_44) and DeLuca et al 2012[^45^](#_ENREF_45) | hCIMT  Restraint: full arm cast 24h/day; Context: home/community; Therapist directed. Content: Shaping and repetitive task practice using movement, reinforcement, repetition and refinement (MR3 cycle), followed by 3 days BiT | 3.7 (18 d cCIMT, 3 d bimanual) | 5-6 | 6 | NR | hCIMT  Restraint: full arm cast 24h/day; Context: home/community; Therapist directed. Content: Shaping and repetitive task practice using movement, reinforcement, repetition and refinement (MR3 cycle) followed by 3 days BiT | 3.7 (18 d cCIMT, 3 d bimanual | 5-6 | 3 | NR |  |
| Chamudot 2018[^46^](#_ENREF_46) | mCIMT  Restraint: mitt; Context: home; Content: activities promoted unilateral hand use | 8 | 1 (OT visit) | NR | 1 x 7/wk | Bimanual  Context: home  Content: activities to encourage symmetrical and asymmetrical bimanual hand use | 8 | 1 (OT visit) | NR | 1 x 7/wk |  |
| Charles 2006[^47^](#_ENREF_47) | mCIMT  Restraint: sling; Context: clinic based groups of 2 to 4; Content: movement training, play, functional therapy | 2 | 5 | 6 | 1 during study  2 x 26 wks post | Maintained previous levels | 2 | NR | NR | NR |  |
| Chen 2013[^48^](#_ENREF_48) , Chen 2014[^49^](#_ENREF_49) , Hsin 2012[^50^](#_ENREF_50) | mCIMT  Restraint: elastic bandage and glove; Context: home; Content: shaping and repetitive task practice | 4 | 2 | 3.5-4 | Restraint: 3.5-4 x 7d/w | Traditional rehab. Context: home: Content: NDT, task training | 4 | 2 | 3.5-4 | Time not specified |  |
| Choudhary 2013[^52^](#_ENREF_52) | mCIMT  Restraint: arm sling  Context: groups of 4; Content: repetitive task practice, shaping | 4 | 2-3 | 2 | 1 x10 days  2 x 30 days | Regular therapy. Context: home; Content: stretch, strength, bilateral hand tasks | 4 | 5 |  | 0.3 h/d |  |
| Christmas 2018[^51^](#_ENREF_51) | mCIMT  Restraint: semi-rigid cast  Context: home/preschool  Content: caregiver directed therapy: repetitive task practice grasp, release, reach, manipulation | 10  3 x 2 wk blocks with 2 wks in between | 7 | 1 | Restraint: 24  mCIMT: 1 | mCIMT  Restraint: manual  Context: home/preschool  Content: caregiver directed therapy: repetitive task practice grasp, release, reach, manipulation | 10  3 x 2 wk blocks with 2 wks in between | 7 | 1 | Restraint: 1 mCIMT 1 |  |
| Deppe 2013[^53^](#_ENREF_53) | Hybrid: mCIMT + BiT;  Restraint: elastic bandage; Context: clinic; Content: shaping and repetitive task practice, sensory tactile stimulation | 4 (3 mCIMT, 1 Bimanual) | 4 | 1 | NR | Bimanual training. Context: Clinic; Clinic; Content: Shaping and repetitive task practice of bimanual activities, sensory tactile stimulation | 4 | 5 | 4 (4 x 1 hr sessions) | NR |  |
| Dong (a)[^17^](#_ENREF_17) | mCIMT. Restraint: resting hand splint; Context: school; Content: Structured fine and gross motor, self-care and play activities + regular school routine | 3 | 5 | 5 (1 shaping, 4 unstructured practice) | NR | Remind to move. Context: school; Content: sensory cuing watch vibrating every 15 mins | 3 | 5 | 5 | NR |  |
| Dong (b)[^103^](#_ENREF_103) | mCIMT. Restraint: resting hand splint; Context: school; Content: Structured fine and gross motor, self-care and play activities + regular school routine | 3 | 5 | 5 (1 shaping, 4 unstructured practice) | NR | Conventional rehab. Context: school; Content: hand splint, strengthening, stretching, NDT | 3 | 2-3 | 1 | NR |  |
| Eliasson et al 2011 (a)[^11^](#_ENREF_11)  Eliasson et al 2011 (b)[^11^](#_ENREF_11) | Eco-CIMT first;  Restraint: glove; Context: individual home or community based; Content: based on AHA assessment, repetitive whole task practice.  Eco-CIMT second | 8  8 | 7  7 |  | 2 with weekly supervision by therapist  2 as above | Usual care. Context: NR; Content: ADL, fine motor training, functional activity-based  Usual care: a/a | 8  8 | 0.25 OT  0.5 PT  0.25 OT  0.5 PT | NR  NR | NR  NR |  |
| Eliasson 2018[^54^](#_ENREF_54) | mCIMT. Restraint: mitten; Context: home; Content: grasping, toy exploration according to ability level | 18 (6 wks mCIMT, 6 wk break, 6 wks mCIMT) | 6 |  | 0.5 | Baby Massage. Context: home; Content: parents massaged body parts. | 18 (6 wks massage, 6 wk break, 6 wks massage) | 6 |  | 0.1-0.5 |  |
| Eugster-Buesch 2012[^55^](#_ENREF_55) | Forced use: Restraint: removable cast; Context: home, clinic: Content: regular therapy | 2 | 7 restraint  1 therapy | 6  NR | 2 | Control group | 2 | 1 | NR | NR |  |
| Facchin et al (b)[^36^](#_ENREF_36) and Fedrizzi et al[^37^](#_ENREF_37) | mCIMT  Restraint: glove; Context: home, clinic: Content: holding, manipulation, ADLs | 10 | 3 | 3 | 3 x 4d/wk | Bimanual training. Context: home, clinic; Content: bimanual play and ADL activities | 10 | 3 | 3 | 3 x 4d/wk |  |
| Facchin 2011[^36^](#_ENREF_36) (c) and Fedrizzi et al[^37^](#_ENREF_37) | mCIMT  Restraint: glove; Context: individual; Content: as above | 10 | 3 | 3 | 3 x 4d/wk | Usual care. Context: NR; Content: NR | 10 | 1-2 | 1 | NR |  |
| Gelkop 2015[^56^](#_ENREF_56) | mCIMT  Restraint: mitt  Context: preschool, kindergarten  Content: repetitive whole and part task practice, ADLs and play | 8 | 6 | 2 | NR | Bimanual therapy. Context: preschool/kindergarten: Content: age appropriate fine and gross motor activities. | 8 | 6 | 2 | NR |  |
| Gordon 2011[^57^](#_ENREF_57) and Brandao 2012 [^58^](#_ENREF_58) | mCIMT  Restraint: sling; Context: clinic day camps, groups of 2 to 5; Content: Shaping and repetitive practice unimanual functional activities and play. | 3 | 5 | 6 | 1 x 26 wks | Bimanual training: Context: clinic day camp; Content: shaping and repetitive practice of goal-directed, symmetrical and asymmetrical activities | 3 | 5 | 6 | 1 x 26 wks |  |
| Hoare 2013[^59^](#_ENREF_59) | mCIMT + BoNT-A; Restraint: glove; Context: individual hospital based and home; Content: BoNT-A injections: maximum dose, 15 U/kg; dilution, 100 U/mL; repetitive task practice. | 8 | 2 | 1 | Glove and home practice  21  Total=98.5h | Bimanual OT +BoNT-A: Context: individual hospital based and home; Content: motor learning and cognitive based repetitive practice bimanual activities | 8 | 2 | 1 | Time not specified.  Total dose = 31.6 hours |  |
| Hwang 2020[^60^](#_ENREF_60) | mCIMT. Constinuous restraint using removeable splint (removed for sleeping and bathing) & CIMT standardized protocol. | 3 | 5 | 2 | NR | Conventional therapy | NR | NR | NR | NR |  |
| Kirton 2016[^61^](#_ENREF_61) & Kuo 2018[^62^](#_ENREF_62) (2/4 trial arms relevant) | hCIMT  Restraint: bivalved cast worn 90% waking hours for 2 weeks of intervention.  Context: clinic, home  Content: Shaping and repetitive task practice unimanual activities | 2 | 5 | 8 | 1 x 10d  0.25 x 22 wks after study | Intensive motor learning  Context: clinic  Content: goal directed UL activities, ADLs, sports, video games, virtual reality | 2 | 5 | 8 | 1 x 10d  0.25 x 22 wks after study |  |
| Klingels 2013[^19^](#_ENREF_19) | mCIMT + Intensive strengthening  Restraint: rigid orthosis; Context: home; Content: list of fine, gross motor, ADLs for unimanual practice and 2 bimanual ADLs + distal strengthening program.  Intensive: Unimanual and bimanual approach focused on hand strength and function. | 10 | 5 mCIMT  3 Int Th | 0.75 (IT) with therapist | 1 h (CIMT)  With caregiver | mCIMT  Restraint: rigid orthosis; Context: home; Content: list of fine, gross motor, ADLs for unimanual practice and 2 bimanual ADLs | 10 | 5 |  | 1. |  |
| Liang 2023[^63^](#_ENREF_63) | mCIMT: Restraint: verbal instructions and physical guidance (holding child’s hand) Context: half day school and half day at home | 8 | 2 | 2.25 |  | BiM: Bimanual activities. Context: half day school and half day at home | 8 | 2 | 2.25 |  |  |
| Lin 2011 [^64^](#_ENREF_64) | mCIMT  Context: home; 1:1 therapist:child.  Content: shaping and repetitive task practice  Restraint when not in therapy: elastic bandage; | 4 | 2 | 4 | Restraint: 3.5-4 | Therapy  Context: home; 1:1 therapist:child. Content: functional unilateral and bilateral activities, NDT techniques, motor learning. | 4 | 2 | 4 | Restraint: 3.5-4 |  |
| Maitre 2020[^65^](#_ENREF_65) | Therapist directed, parent administered bimanual play, soft-constraint 6h/day, reach training of affected UE, graduated motor-senory training, parent education | 4 | 7 |  | Activities: 0.6. Restraint: 6 | Bimanual play with suggested toys encourage activity btn parents and children. | 4 | 7 |  | 0.3 |  |
| Maring 2019[^66^](#_ENREF_66) | mCIMT  Restraint: bivalve cast for 113 h  Context: clinic  mCIMT with 1:1 therapist + home program | 10 | 3 | 2 | ? | Restraint: bivalve cast 113h over period.  mCIMT provided by parents in home, using home program given by therapist | 10 |  |  | 33 |  |
| Mohamed 2021[^104^](#_ENREF_104) | mCIMT + UC  Restraint: sling  Context: Clinic  Content: Exercises for UL movement, grasp. | 12 | 5 | 1 + 1 UC | NR | Mirror Therapy  Context: clinic  Content: 30 x 20 inch mirror. Exercises performed bilaterally and symmetrically | 12 | 5 | 1+ 1 UC | NR |  |
| Ostadzadeh 2023[^67^](#_ENREF_67) | mCIMT. Context: home, therapist coach families.  Restraint: splint. Content: Fine motor | 4 | 3 |  | 1 | mCIMT + GDT . Context: home, therapist coach families. Restraint: splint. Content: occupation-based activity | 4 | 3 |  | 1 |  |
| Palomo-Carrion 2020[^68^](#_ENREF_68) | mCIMT  Restraint: NR  Context: home  Content: Activities targeting movement components and grasp | 5 | 5 |  | 2 (wkly fu by therapist) | Unimanual training without constraint  Context: home  Content: Activities targeting movement components and grasp | 5 | 5 |  | 2 (wkly fu by therapist) |  |
| Palomo-Carrion 2021[^69^](#_ENREF_69) | mCIMT-BIT  Restraint: Glove Context: home  Content: 12 tasks (6/hr)/wk for quantity and quality of movement | 10 (8 mCIMT  2 BIT) | 5 |  | 2 (wkly fu by therapist) | BIT-mCIMT  Restraint: Glove  Context: home  Content: 12 tasks (6/hr)/wk for quantity and quality of movement | 10 (8 Bit 2 mCIMT) | 5 |  | 2 (wkly fu by therapist) |  |
| Ramey 2021[^70^](#_ENREF_70) | a) CIMT_60hr+cast  Restraint: full arm cast  Context: home  Content: 17d CIMT, 3d bimanual, massed practice, shaping  b) CIMT_60hr+mitt  c) CIMT_30hr+cast  d) CIMT_30hr+mitt | 4  4  4  4 | 5  5  5  5 | 6  6  3  3 | NR  NR  NR  NR | UC – content NR | NR | NR | Mean 4.5hrs | NR |  |
| Rostami_a 2012_Dis & Rehab (a)[^71^](#_ENREF_71) | mCIMT  Restraint: splint; Context: home based individual; Content: reach, grasp, manipulate, fine motor, ADL | 3 | 3 | 1.5 | 1 | mCIMT: Restraint: splint; Context: clinic based individual; Content as per intervention group | 3 | 3/wk | 1.5 h | 1 h/d |  |
| Rostami et al., 2012 (Neuro)[^105^](#_ENREF_105) | mCIMT  Restraint: splint; Context: home based individual; Content: reach, grasp, manipulate, fine motor, ADL | 4 | 3 | 1.5 | Restraint: 5 | Control group. Context: NR; Content: NDT, stretching, ROM | 4 | 2/wk | 0.5 h | NR |  |
| Sakzewski et al., 2011 (DMCN) [^72^](#_ENREF_72) Sakzewski et al., 2011 (APMR)[^74^](#_ENREF_74)  Sakzewski et al., 2011 (NNR)[^73^](#_ENREF_73) | mCIMT  Restraint: mitt;  Context: community groups of 8 to13; Content: repetitive task practice, circus themed | 2 | 5 | 6 h | NR | Bimanual training Context: community groups of 8-13; Content: repetitive practice bimanual activities | 2 | 5/wk | 6 h | Nil |  |
| Sakzewski 2015_RIDD[^75^](#_ENREF_75) | mCIMT  Restraint: mitt; Context: community day camp; Content: repetitive unimanual activity based, practice, circus themed | 1 | 5 | 6 h | NR | Bimanual training  Context: community groups of 8-13; Content: goal directed repetitive practice bimanual activities | 1 | 5/wk | 6 h | Nil |  |
| Sakzewski 2015[^76^](#_ENREF_76) | Therapist directed in a camp format. mCIMT first week, Bimanual therapy seconds week. | 2 | 5 | 6  (Total direct therapy 45 h, indirect therapy 10h) | No | Occupational Therapy + home program | OT: 6  HP: 12 | OT: 1  HP: 6 | OT: 1h direct therapy, 0.5h HP develop- ment and demonstrationHP: 0.5h | Total dose 45h (9h direct and 36 h HP) |  |
| Smania et al., 2009[^77^](#_ENREF_77) | mCIMT  Restraint: mitt; Context: individual; Content: repetitive practice, play | 5 | 2 | 1 | Restraint: 8 | Physiotherapy  Context: NR  Content: motor learning based. | 5 | 2 | 1 | NR |  |
| Sung et al., 2005[^78^](#_ENREF_78) | Forced use therapy+ OT (categorised mCIMT)  Restraint: short arm cast worn 6 weeks.  Context: outpatient clinic; Content: stretch, reach, grasp, manipulate, functional training | 6 therapy & 6 cast | 2 | 0.5 | Restraint: 24 | OT  Context: outpatient clinic; Content: as per intervention group | 6 | 2 | 0.5 | NR |  |
| Taub 2004[^79^](#_ENREF_79) and Deluca 2006[^80^](#_ENREF_80) | sCIMT  Restraint: long arm bivalve cast; Context: individual clinic based; Content: repetitive task practice, shaping reach, grasp, weight bearing, manipulate, ADLs | 3 | 7 | 6 | NR | Standard OT/PT  Context: NR  Content: NR | 3 | 1-4 | 2.2 (mean) | NR |  |
| Taub et al., 2011[^81^](#_ENREF_81) | hCIMT  Restraint: long arm cast 24h/day; Context: home & community based; Content: shaping and repetitive task practice in play and ADLs. Bimanual transfer package with bilateral activities. | 3  (13d CIMT, 2d BIM) | 7  (5 therapist; 2 parent) | 6 | Not reported in detail | Usual care  Context: NR; Content: NR | 3 | 1 | 1 | NR |  |
| Vaghela 2014[^82^](#_ENREF_82) | CIMT - function and play based & passive stretching, Details /time unclear. | 12 | ? | 6 | Restraint with bivalve cast 90% waking hours | mCIMT – play based & passive stretching. Details/ time unclear. | 12 | ? | 1 | Restraint 1 hour per day. |  |
| Wallen 2011[^83^](#_ENREF_83) | mCIMT  Restraint: mitt; Context: home, school, preschool; Content: practice of movements required for ADL goals, repetitive movements in play | 8 | 1 | 1 | 2 x 7 d/wk | Occupational therapy  Context: home, clinic; Content: goal-directed, stretch, cast, splint, motor training, environmental modification | 8 | 1 | 1 | 0.3 |  |
| Xu 2012 [^84^](#_ENREF_84) | mCIMT  Restraint: splint worn all day (time out of splint max 30min).  Context: hospital in groups of 2 to 4 and home. Content: structured play and functional activities targeting specific movements | 2 | 5 | 3 | 1 during study  2 for 26wks after CIMT | Occupational Therapy  Context: home, hospital; Content: NDT, task specific training, strength, stretch | 2 | 5 | 3 | 1 during study  2 for 26wks after study |  |
| Yu 2012 [^85^](#_ENREF_85) | mCIMT + traditional rehab  Restraint: sling and splint  Context: Clinic, group.  Content: specific fine motor tasks carried out according to protocol | mCIMT:10  rehab: 10 | mCIMT: 2  rehab: 2 | mCIMT:1  rehab: 0.5 | NR | Traditional rehabilitation  Context: clinic, group.  Content: ‘traditional rehabilitation’, no detail provided | 10 | 2 | 0.5 | NR |  |
| Zafer 2016 [^86^](#_ENREF_86) | mCIMT  Restraint: mitt and sling; Context: home; parent led. Content: unilateral reach, grasp, manipulation and release, weightbearing. | 2 | 6 |  | Training 2  Restraint 6 | Bimanual  Context: home; parent led. Content: ADL practice of bimanual daily activities | 2 | 6 |  | 2 |  |
| ***Mirror Therapy*** | |  |  |  |  |  |  |  |  |  |  |
| Bruchez 2016[^87^](#_ENREF_87) | Used mirror to look at reflection of non-paretic limb. Did 3 x 10 reps of 7 symmetrical, simultaneous hand and upper limb movements in front of mirror | 5 | 5 |  | 0.25 | 3 x 10 reps of 7 symmetrical, simultaneous hand and upper limb movements, no mirror (looked at paretic limb) | 5 | 5 |  | 0.25 |  |
| Elsepaee 2016[^88^](#_ENREF_88) | Physical therapy: gross and fine motor using functional fine motor activities. Mirror therapy with fine motor activities | 4 | 7/wk (“daily”, “28 days application” | 1 hour therapy + 0.5hr mirror thpy |  | Physical therapy: gross and fine motor using functional fine motor activities | 4 | 7/wk (“daily”, “28 days application” | 1 |  |  |
| Gygax et al 2011 [^14^](#_ENREF_14) | Mirror therapy and bimanual activities first: bilateral thumb-fingers pinch and grasp and pronation supination; Context: home | 3 | 7 |  | 0.25 | Bimanual activities as per treatment group, no mirror; context, home | 3 | 7 |  | 0.25 |  |
| Kara 2020[^89^](#_ENREF_89) | Mirror therapy (grasp and release activities) and bilateral UL power/strength exercises | 12 | 3 | 30min mirror + 15 min strength exercises | No | OT (fine motor skill activities of writing cutting, play with cubes) + bilateral UL power/strength exercises | 12 | 3 | 0.5 | No |  |
| Narimani 2019 [^90^](#_ENREF_90) | Mirror therapy and usual care. Mirror therapy: bilateral and symmetrical exercise and tasks | 6 | 3 | 0.5 h + mirror activities (time not specified – 10 reps of each activity) | NR | Usual care (NDT based) | NR | NR | NR | NR |  |
| ***Other UL interventions*** | | | | | | | | | | | |
| Cameron 2017[^91^](#_ENREF_91) | CO-OP: Task specific training combining motor learning theories and cognitive approach. Problem solving strategy “goal-plan-do-check”.  Context: individual, mainly home based | 10 | 1 | 1 | NR | Care as usual: 62% direct skill acquisition; 38% remediating underlying impairments  Context: individual, mainly home based | 10 | 1 | 1 | NR |  |
| Holmstrom 2019[^92^](#_ENREF_92) | Small Step Program: 3 foci – hand use, mobility and communication. Each foci last 6 weeks. Hand use and mobility steps repeated twice. Parent coaching to do daily activities. Child-active, goal orientated, intensive practice principles. | 30 | 1 home visit (apart from communication foci which had 4 home visits over 6 wk block. | NR | Daily intervention by parents | Standard care | 30 | 0.25 | NR | NR |  |
| Jackman 2018[^93^](#_ENREF_93) | CO-OP: Task specific training combining motor learning theories and cognitive approach. Problem solving strategy “goal-plan-do-check”.  Splint: wrist cock-up  Context: clinic/based  in groups of 2-5. | 2 | 5 | 1 CO-OP  Splint wear during CO-OP | Total mean home practice CO-OP +splint 7.1hrs; splint only group=5.5h | CO-OP: Task specific training combining motor learning theories and cognitive approach. Problem solving strategy “goal-plan-do-check”.  Context: clinic/based  in groups of 2-5. | 2 | 5 | 1 CO-OP  Splint wear during CO-OP | Total mean home practice CO-OP + splint= 6.2h |  |
| Ko 2020[^94^](#_ENREF_94) | Task /goal-directed training. Practice specific ADL. Group setting | 8 | 2/wk | 1 |  | Traditional therapy (PT & OT) based on normalisation of quality of movement. Individual | 8 | 2 | 1 (0.5 PT, 0.5 OT) |  |  |
| McLean 2017[^18^](#_ENREF_18) | Emphasise/explore somatosensory features during meaningful activities. Graded. Usual care continued | 6 | 3 | 1 |  | Usual care |  |  |  | NR |  |
| Law 2011[^95^](#_ENREF_95) | Child focused:  Remediation of impairments using stretch, cast, strength, weight bearing and facilitation of normal movement | 26 | 0.7-0.9 | NR | NR | Context focused: Goal-directed, task and environment adaptation | 26 | 0.7-0.9 | NR | NR |  |
| Moon 2017[^96^](#_ENREF_96) | Task oriented training: repetitive reaching, ring activity, stacking cup, therapist feedback. Usual care: occupational therapy not described | 4 | 2 | 0.3 TOT, 0.7 UC | NR | Usual care occupational therapy: not described | 4 | 2 | 1 | NR |  |
| Novak 2009a[^12^](#_ENREF_12)  Novak 2009b[^12^](#_ENREF_12) | OT home program: goal-directed, parent education, handwriting, strength, play  OT home program: details as above | 8  4 | 4.5  4.25 |  | 0.25 (mean)  0.25 (mean) | Control group  Control group | 8  8 | NR  NR | NR  NR | NR  NR |  |
| Sousa 2021[^97^](#_ENREF_97) | CO-OP  Context: Individual, clinic setting  Content: Task specific training combining motor learning theories and cognitive approach. Problem solving strategy “goal-plan-do-check”. | 5 | 2 | 0.75 | NR | OT:  Context: Individual, clinic setting  Content: ADL training, school-related skills, social, motor and perceptual skills, strengthening, stretching and assistive devices (no cognitive strategies) | 6 | 2 | .75 | NR |  |
| Verghaegh 2023[^13^](#_ENREF_13) | HEI- Home based intervention with video coaching of parents. Goals set by parents together with therapist. Intervention consists of unimanual and bimanual training, sensory-motor experiences, just right challenge. | 8 | 7 |  | 0.5 | HEI + SSR: HEI same as described. SSR involved multisensory tactile (vibration), visual (lights), auditory (song) input on wristband worn on affected arm | 8 | 7 |  | 0.5  + SSR- 20-40 sec at random for 10 mins, 3x/day. |  |
| Yuan 2023[^98^](#_ENREF_98) | HEI – Context: home and hospital based. Content:’GAME’ principles of goal directed, enriched environment, motivation. | 12 | 5 | 1 |  | Usual care. Context: hospital based. | 12 | 5 | 2 |  |  |
| Wallen 2007[^99^](#_ENREF_99) | OT intervention (at hospital) | 12 | 1 | 1 |  | Usual care |  |  |  |  |  |

Wk indicates week; h, hour; d, day; mo, month; a/a, as above; ADLs, activities of daily living; BIM, bimanual; CIMT, constraint induced movement therapy; m, modified; HABIT, Hand Arm Bimanual Intensive Training; HEI Home-based Early Intervention; NDT, Neurodevelopmental Treatment; NR, not reported; OT, occupational therapy; PT, physiotherapy; kg, kilogram; rehab, rehabilitation; ROM, range of motion; RAT, robot-assisted therapy; SSR, Sensory Stimulation Reminder.

# References

1. Sgandurra G, Ferrari, Adriano, , Cossu G, Guzzetta, Andrea, , Biagi L, Tosetti M, Fogassi L, Cioni G. Upper Limb Children Action-observation Training (UP-CAT): A Randomised Controlled Trial in Hemiplegic Cerebral Palsy. *BMC Neurology.* 2011;11(1).

2. Gordon AM, Schneider JA, Chinnan A, Charles JR. Efficacy of a hand-arm bimanual intensive therapy (HABIT) in children with hemiplegic cerebral palsy: a randomized control trial. *Developmental Medicine & Child Neurology.* 2007;49(11):830-838.

3. Figueiredo PRP, Mancini MC, Feitosa AM, et al. Hand-arm bimanual intensive therapy and daily functioning of children with bilateral cerebral palsy: a randomized controlled trial. *Developmental Medicine and Child Neurology.* 2020.

4. Araneda R, Herman E, Delcour L, et al. Mirror movements after bimanual intensive therapy in children with unilateral cerebral palsy: A randomized controlled trial. *Dev Med Child Neurol.* 2022.

5. Bleyenheuft Y, Ebner-Karestinos D, Surana B, et al. Intensive upper- and lower-extremity training for children with bilateral cerebral palsy: a quasi-randomized trial. *Developmental Medicine & Child Neurology.* 2017;59(6):625-633.

6. Taub E, Miller NE, Novack TA, et al. Technique to improve chronic motor deficit after stroke. . *Arch Phys Med Rehabil.* 1993;74:347-354.

7. Eliasson AC, Krumlinde-Sundholm L, Gordon AM, et al. Guidelines for future research in constraint-induced movement therapy for children with unilateral cerebral palsy: an expert consensus. *Dev Med Child Neurol.* 2014;56(2):125-137.

8. Hoare BJ, Wallen MA, Thorley MN, Jackman ML, Carey LM, Imms C. Constraint-induced movement therapy in children with unilateral cerebral palsy. *Cochrane Database Syst Rev.* 2019;4:CD004149.

9. Gimeno H, Polatajko H. The Cognitive Orientation to daily Occupational Performance approach in childhood-onset disabilities. *Dev Med Child Neurol.* 2025;67(8):977-985.

10. Polatajko HJ, Mandich A. *Enabling occupation in children : the cognitive orientation to daily occupational performance (CO-OP) approach.* Ontario, Ottowa: CAOT Publications ACE.; 2004.

11. Eliasson AC, Shaw K, Berg E, Krumlinde-Sundholm L. An ecological approach of Constraint Induced Movement Therapy for 2-3-year-old children: a randomized control trial. *Research in Developmental Disabilities.* 2011;32(6):2820-2828.

12. Novak I, Cusick A, Lannin N. Occupational therapy home programs for cerebral palsy: double-blind, randomized, controlled trial. *Pediatrics.* 2009;124(4):e606-614.

13. Verhaegh APM, Groen BE, Aarts PBM, et al. Multisensory Stimulation and Priming (MuSSAP) in 4-10 Months Old Infants with a Unilateral Brain Lesion: A Pilot Randomised Clinical Trial. *Occup Ther Int.* 2023;2023:8128407.

14. Gygax MJ, Schneider P, Newman CJ. Mirror therapy in children with hemiplegia: a pilot study. *Developmental Medicine and Child Neurology.* 2011;53(5):473-476.

15. Te Velde A, Morgan C, Finch-Edmondson M, et al. Neurodevelopmental Therapy for Cerebral Palsy: A Meta-analysis. *Pediatrics.* 2022;149(6).

16. Vaughan-Graham J, C. C. Defining a Bobath clinical framework - a modified e-Delphi study. *Physiother Theory Pract.* 2016;32:612-627.

17. Dong VA, Fong KN, Chen YF, Tseng SS, Wong LM. 'Remind-to-move' treatment versus constraint-induced movement therapy for children with hemiplegic cerebral palsy: a randomized controlled trial. *Developmental Medicine & Child Neurology.* 2017;59(2):160-167.

18. McLean B, Taylor S, Blair E, Valentine J, Carey L, Elliott C. Somatosensory Discrimination Intervention Improves Body Position Sense and Motor Performance in Children With Hemiplegic Cerebral Palsy. *American Journal of Occupational Therapy.* 2017;71(3):1-9.

19. Klingels K, Feys H, Molenaers G, et al. Randomized trial of modified constraint-induced movement therapy with and without an intensive therapy program in children with unilateral cerebral palsy. *Neurorehabilitation & Neural Repair.* 2013;27(9):799-807.

20. Simon-Martinez C, Mailleux L, Hoskens J, et al. Randomized controlled trial combining constraint-induced movement therapy and action-observation training in unilateral cerebral palsy: clinical effects and influencing factors of treatment response. *Therapeutic Advances in Neurological Disorders.* 2020;13.

21. Kuo HC, Gordon AM, Henrionnet A, Hautfenne S, Friel KM, Bleyenheuft Y. The effects of intensive bimanual training with and without tactile training on tactile function in children with unilateral spastic cerebral palsy: A pilot study. *Res Dev Disabil.* 2016;49-50:129-139.

22. Beani E, Menici V, Sicola E, et al. Effectiveness of the home-based training program Tele-UPCAT (Tele-monitored UPper Limb Children Action Observation Training) in unilateral cerebral palsy: a randomized controlled trial. *Eur J Phys Rehabil Med.* 2023.

23. Buccino G, Arisi D, Gough P, et al. Improving upper limb motor functions through action observation treatment: a pilot study in children with cerebral palsy. *Developmental Medicine & Child Neurology.* 2012;54(9):822-828.

24. Buccino G, Molinaro A, Ambrosi C, et al. Action Observation Treatment Improves Upper Limb Motor Functions in Children with Cerebral Palsy: A Combined Clinical and Brain Imaging Study. *Neural Plasticity.* 2018;2018:4843985.

25. Elbagoury WS, El-Saeed TM, Olama KA, Kamel MI. Functional-outcomes-of-verbaldirected-training-versus-visualdirected-training-in-children-with-unilateral-cerebral-palsy. *26.* 2022;1:1205-1210.

26. Kim DH, An DH, Yoo WG. Effects of live and video form action observation training on upper limb function in children with hemiparetic cerebral palsy. *Technology & Health Care.* 2018;26(3):437-443.

27. Kim DH. Comparison of short- and long-time action observation training (AOT) on upper limb function in children with cerebral palsy. *Physiotherapy Practice & Research.* 2020;41(1):53-58.

28. Kirkpatrick E, Pearse J, James P, Basu A. Effect of parent-delivered action observation therapy on upper limb function in unilateral cerebral palsy: a randomized controlled trial. *Developmental Medicine & Child Neurology.* 2016;58(10):1049-1056.

29. Palomo-Carrion R, Zuil-Escobar JC, Cabrera-Guerra M, Barreda-Martinez P, Martinez-Cepa CB. Mirror and action observation therapy in children with unilateral spastic cerebral palsy: a feasibility study. *Revista de Neurologia.* 2022;75(11):325-332.

30. Quadrelli E, Anzani A, Ferri M, et al. Electrophysiological correlates of action observation treatment in children with cerebral palsy: A pilot study. *Developmental Neurobiology.* 2019;79(11-12):934-948.

31. Sgandurra G, Ferrari A, Cossu G, Guzzetta A, Fogassi L, Cioni G. Randomized trial of observation and execution of upper extremity actions versus action alone in children with unilateral cerebral palsy. *Neurorehabilitation & Neural Repair.* 2013;27(9):808-815.

32. Araneda R, Ebner-Karestinos D, Paradis J, et al. Changes Induced by Early Hand-Arm Bimanual Intensive Therapy Including Lower Extremities in Young Children With Unilateral Cerebral Palsy: A Randomized Clinical Trial. *JAMA Pediatrics.* 2024;178(1):19-28.

33. Bleyenheuft Y, Arnould C, Brandao MB, Bleyenheuft C, Gordon AM. Hand and Arm Bimanual Intensive Therapy Including Lower Extremity (HABIT-ILE) in Children With Unilateral Spastic Cerebral Palsy: A Randomized Trial. *Neurorehabilitation & Neural Repair.* 2015;29(7):645-657.

34. Brandao MB, Ferre C, Kuo HC, et al. Comparison of Structured Skill and Unstructured Practice During Intensive Bimanual Training in Children With Unilateral Spastic Cerebral Palsy. *Neurorehabilitation & Neural Repair.* 2014;28(5):452-461.

35. Brandao MB, Mancini MC, Ferre CL, et al. Does Dosage Matter? A Pilot Study of Hand-Arm Bimanual Intensive Training (HABIT) Dose and Dosing Schedule in Children with Unilateral Cerebral Palsy. *Physical & Occupational Therapy in Pediatrics.* 2018;38(3):227-242.

36. Facchin P, Rosa-Rizzotto M, Pozza LVD, et al. Multisite Trial Comparing the Efficacy of Constraint-Induced Movement Therapy with that of Bimanual Intensive Training in Children with Hemiplegic Cerebral Palsy. *American Journal of Physical Medicine & Rehabilitation.* 2011;90(7):539-553.

37. Fedrizzi E, Rosa-Rizzotto M, Turconi AC, et al. Unimanual and bimanual intensive training in children with hemiplegic cerebral palsy and persistence in time of hand function improvement: 6-month follow-up results of a multisite clinical trial. *Journal of Child Neurology.* 2013;28(2):161-175.

38. Ferre CL, Brandao M, Surana B, Dew AP, Moreau NG, Gordon AM. Caregiver-directed home-based intensive bimanual training in young children with unilateral spastic cerebral palsy: a randomized trial. *Developmental Medicine & Child Neurology.* 2017;59(5):497-504.

39. Aarts PB, Jongerius PH, Geerdink YA, van Limbeek J, Geurts AC. Effectiveness of modified constraint-induced movement therapy in children with unilateral spastic cerebral palsy: a randomized controlled trial. *Neurorehabilitation & Neural Repair.* 2010;24(6):509-518.

40. Geerdink Y, Aarts P, Geurts AC. Motor learning curve and long-term effectiveness of modified constraint-induced movement therapy in children with unilateral cerebral palsy: a randomized controlled trial. *Research in Developmental Disabilities.* 2013;34(3):923-931.

41. Abd El-Kafy EM, Elshemy SA, Alghamdi MS. Effect of constraint-induced therapy on upper limb functions: a randomized control trial. *Scandinavian Journal of Occupational Therapy.* 2014;21(1):11-23.

42. Al-Oraibi S, Eliasson AC. Implementation of constraint-induced movement therapy for young children with unilateral cerebral palsy in Jordan: a home-based model. *Disability & Rehabilitation.* 2011;33(21-22):2006-2012.

43. de Brito Brandao M, Mancini MC, Vaz DV, Pereira de Melo AP, Fonseca ST. Adapted version of constraint-induced movement therapy promotes functioning in children with cerebral palsy: a randomized controlled trial. *Clinical Rehabilitation.* 2010;24(7):639-647.

44. Case-Smith J, DeLuca SC, Stevenson R, Ramey SL. Multicenter randomized controlled trial of pediatric constraint-induced movement therapy: 6-month follow-up. *American Journal of Occupational Therapy.* 2012;66(1):15-23.

45. DeLuca SC, Case-Smith J, Stevenson R, Ramey SL. Constraint-induced movement therapy (CIMT) for young children with cerebral palsy: effects of therapeutic dosage. *Journal of Pediatric Rehabilitation Medicine.* 2012;5(2):133-142.

46. Chamudot R, Parush S, Rigbi A, Horovitz R, Gross-Tsur V. Effectiveness of Modified Constraint-Induced Movement Therapy Compared With Bimanual Therapy Home Programs for Infants With Hemiplegia: A Randomized Controlled Trial. *American Journal of Occupational Therapy.* 2018;72(6):7206205010p7206205011-7206205010p7206205019.

47. Charles JR, Wolf SL, Schneider JA, Gordon AM. Efficacy of a child-friendly form of constraint-induced movement therapy in hemiplegic cerebral palsy: a randomized control trial. *Developmental Medicine & Child Neurology.* 2006;48(8):635-642.

48. Chen CL, Kang LJ, Hong WH, Chen FC, Chen HC, Wu CY. Effect of therapist-based constraint-induced therapy at home on motor control, motor performance and daily function in children with cerebral palsy: a randomized controlled study. *Clinical Rehabilitation.* 2013;27(3):236-245.

49. Chen HC, Chen CL, Kang LJ, Wu CY, Chen FC, Hong WH. Improvement of upper extremity motor control and function after home-based constraint induced therapy in children with unilateral cerebral palsy: immediate and long-term effects. *Archives of Physical Medicine & Rehabilitation.* 2014;95(8):1423-1432.

50. Hsin YJ, Chen FC, Lin KC, Kang LJ, Chen CL, Chen CY. Efficacy of Constraint-Induced Therapy on Functional Performance and Health-Related Quality of Life for Children With Cerebral Palsy: A Randomized Controlled Trial. *Journal of Child Neurology.* 2012;27(8):992-999.

51. Christmas PM, Sackley C, Feltham MG, Cummins C. A randomized controlled trial to compare two methods of constraint-induced movement therapy to improve functional ability in the affected upper limb in pre-school children with hemiplegic cerebral palsy: CATCH TRIAL. *Clinical Rehabilitation.* 2018;32(7):909-918.

52. Choudhary A, Gulati S, Kabra M, et al. Efficacy of modified constraint induced movement therapy in improving upper limb function in children with hemiplegic cerebral palsy: a randomized controlled trial. *Brain & Development.* 2013;35(9):870-876.

53. Deppe W, Thuemmler K, Fleischer J, Berger C, Meyer S, Wiedemann B. Modified constraint-induced movement therapy versus intensive bimanual training for children with hemiplegia - a randomized controlled trial. *Clinical Rehabilitation.* 2013;27(10):909-920.

54. Eliasson AC, Nordstrand L, Ek L, et al. The effectiveness of Baby-CIMT in infants younger than 12 months with clinical signs of unilateral-cerebral palsy; an explorative study with randomized design. *Research in Developmental Disabilities.* 2018;72:191-201.

55. Eugster-Buesch F, de Bruin ED, Boltshauser E, et al. Forced-use therapy for children with cerebral palsy in the community setting: a single-blinded randomized controlled pilot trial. *Journal of Pediatric Rehabilitation Medicine.* 2012;5(2):65-74.

56. Gelkop N, Burshtein DG, Lahav A, et al. Efficacy of constraint-induced movement therapy and bimanual training in children with hemiplegic cerebral palsy in an educational setting. *Physical & Occupational Therapy in Pediatrics.* 2015;35(1):24-39.

57. Gordon AM, Hung YC, Brandao M, et al. Bimanual training and constraint-induced movement therapy in children with hemiplegic cerebral palsy: a randomized trial. *Neurorehabilitation & Neural Repair.* 2011;25(8):692-702.

58. de Brito Brandao M, Gordon AM, Mancini MC. Functional impact of constraint therapy and bimanual training in children with cerebral palsy: a randomized controlled trial. *American Journal of Occupational Therapy.* 2012;66(6):672-681.

59. Hoare B, Imms C, Villanueva E, Rawicki HB, Matyas T, Carey L. Intensive therapy following upper limb botulinum toxin A injection in young children with unilateral cerebral palsy: a randomized trial. *Developmental Medicine & Child Neurology.* 2013;55(3):238-247.

60. Hwang YS, Kwon JY. Effects of Modified Constraint-Induced Movement Therapy in Real-World Arm Use in Young Children with Unilateral Cerebral Palsy: A Single-Blind Randomized Trial. *Neuropediatrics.* 2020;51(4):259-266.

61. Kirton A, Andersen J, Herrero M, et al. Brain stimulation and constraint for perinatal stroke hemiparesis: The PLASTIC CHAMPS Trial. *Neurology.* 2016;86(18):1659-1667.

62. Kuo HC, Zewdie E, Ciechanski P, Damji O, Kirton A. Intervention-Induced Motor Cortex Plasticity in Hemiparetic Children With Perinatal Stroke. *Neurorehabil Neural Repair.* 2018;32(11):941-952.

63. Liang KJ, Chen HL, Huang CW, Wang TN. Efficacy of Constraint-Induced Movement Therapy Versus Bimanual Intensive Training on Motor and Psychosocial Outcomes in Children With Unilateral Cerebral Palsy: A Randomized Trial. *Am J Occup Ther.* 2023;77(4).

64. Lin KC, Wang TN, Wu CY, et al. Effects of home-based constraint-induced therapy versus dose-matched control intervention on functional outcomes and caregiver well-being in children with cerebral palsy. *Research in Developmental Disabilities.* 2011;32(5):1483-1491.

65. Maitre NL, Jeanvoine A, Yoder PJ, et al. Kinematic and Somatosensory Gains in Infants with Cerebral Palsy After a Multi-Component Upper-Extremity Intervention: A Randomized Controlled Trial. *Brain Topography.* 2020.

66. Maring J, Wentzell E. Constraint Induced Movement Therapy: Impact of Setting on Outcomes. *Journal of Allied Health.* 2019;48(3):e73-e77.

67. Ostadzadeh A, Amini M, Hassani Mehraban A, Maroufizadeh S, Farajzadeh A. The Effect of Occupation-Based Modified Constraint-Induced Movement Therapy on the Participation of Children with Cerebral Palsy: A Single-Blind Randomized Controlled Trial. *Iran J Child Neurol.* 2023;17(2):39-54.

68. Palomo-Carrion R, Pinero-Pinto E, Ando-LaFuente S, Ferri-Morales A, Bravo-Esteban E, Romay-Barrero H. Unimanual Intensive Therapy with or without Unaffected Hand Containment in Children with Hemiplegia. A Randomized Controlled Pilot Study. *Journal of Clinical Medicine.* 2020;9(9):14.

69. Palomo-Carrion R, Lirio-Romero C, Ferri-Morales A, Jovellar-Isiegas P, Cortes-Vega MD, Romay-Barrero H. Combined intensive therapies at home in spastic unilateral cerebral palsy with high bimanual functional performance. What do they offer? A comparative randomised clinical trial. *Therapeutic Advances in Chronic Disease.* 2021;12:20406223211034996.

70. Ramey SL, DeLuca SC, Stevenson RD, Conaway M, Darragh AR, Lo W. Constraint-induced movement therapy for cerebral palsy: A randomized trial. *Pediatrics.* 2021;148(5).

71. Rostami HR, Malamiri RA. Effect of treatment environment on modified constraint-induced movement therapy results in children with spastic hemiplegic cerebral palsy: a randomized controlled trial. *Disability & Rehabilitation.* 2012;34(1):40-44.

72. Sakzewski L, Ziviani J, Abbott DF, Macdonell RAL, Jackson GD, Boyd RN. Randomized trial of constraint-induced movement therapy and bimanual training on activity outcomes for children with congenital hemiplegia. *Developmental Medicine and Child Neurology.* 2011;53(4):313-320.

73. Sakzewski L, Ziviani J, Abbott DF, Macdonell RA, Jackson GD, Boyd RN. Equivalent retention of gains at 1 year after training with constraint-induced or bimanual therapy in children with unilateral cerebral palsy. *Neurorehabilitation & Neural Repair.* 2011;25(7):664-671.

74. Sakzewski L, Ziviani J, Abbott DF, Macdonell RA, Jackson GD, Boyd RN. Participation Outcomes in a Randomized Trial of 2 Models of Upper-Limb Rehabilitation for Children With Congenital Hemiplegia. *Archives of Physical Medicine & Rehabilitation.* 2011;92(4):531-539.

75. Sakzewski L, Provan K, Ziviani J, Boyd RN. Comparison of dosage of intensive upper limb therapy for children with unilateral cerebral palsy: how big should the therapy pill be? *Research in Developmental Disabilities.* 2015;37:9-16.

76. Sakzewski L, Miller L, Ziviani J, et al. Randomized comparison trial of density and context of upper limb intensive group versus individualized occupational therapy for children with unilateral cerebral palsy. *Developmental Medicine & Child Neurology.* 2015;57(6):539-547.

77. Smania N, Aglioti SM, Cosentino A, et al. A modified constraint-induced movement therapy (CIT) program improves paretic arm use and function in children with cerebral palsy. *European journal of physical & rehabilitation medicine.* 2009;45(4):493-500.

78. Sung IY, Ryu JS, Pyun SB, Yoo SD, Song WH, Park MJ. Efficacy of forced-use therapy in hemiplegic cerebral palsy. *Archives of Physical Medicine & Rehabilitation.* 2005;86(11):2195-2198.

79. Taub E, Ramey SL, DeLuca S, Echols K. Efficacy of constraint-induced movement therapy for children with cerebral palsy with asymmetric motor impairment. *Pediatrics.* 2004;113(2):305-312.

80. Deluca SC, Echols K, Law CR, Ramey SL. Intensive pediatric constraint-induced therapy for children with cerebral palsy: randomized, controlled, crossover trial. *Journal of Child Neurology.* 2006;21(11):931-938.

81. Taub E, Griffin A, Uswatte G, Gammons K, Nick J, Law CR. Treatment of Congenital Hemiparesis With Pediatric Constraint-Induced Movement Therapy. *Journal of Child Neurology.* 2011;26(9):1163-1173.

82. Vaghela VG. To Study the effects of Mcimt Versus Cimt for Young Children with Spastic Hemiplegic Cerebral Palsy-- A Comparitive Study. *Indian Journal of Physiotherapy & Occupational Therapy.* 2014;8(2):136-141.

83. Wallen M, Ziviani J, Naylor O, Evans R, Novak I, Herbert RD. Modified constraint-induced therapy for children with hemiplegic cerebral palsy: a randomized trial. *Developmental Medicine & Child Neurology.* 2011;53(12):1091-1099.

84. Xu K, Wang L, Mai J, He L. Efficacy of constraint-induced movement therapy and electrical stimulation on hand function of children with hemiplegic cerebral palsy: a controlled clinical trial. *Disability & Rehabilitation.* 2012;34(4):337-346.

85. Yu J, Kang H, Jung J. Effects of modified constraint-induced movement therapy on hand dexterity, grip strength and activities of daily living of children with cerebral palsy: a randomized control trial. *Journal of physical therapy science.* 2012;24(10):1029‐1031.

86. Zafer H, Amjad I, Malik AN, Shaukat E. Effectiveness of constraint induced movement therapy as compared to bimanual therapy in upper motor function outcome in child with hemiplegic cerebral palsy. *Pakistan Journal of Medical Sciences.* 2016;32(1):181-184.

87. Bruchez R, Gygax MJ, Roches S, et al. Mirror therapy in children with hemiparesis: a randomized observer-blinded trial. *Developmental Medicine and Child Neurology.* 2016;58(9):970-978.

88. Elsepaee MI, Elhadidy EI, Emara HA, Nawar EAE. EFFECT OF MIRROR VISUAL FEEDBACK ON HAND FUNCTIONS IN CHILDREN WITH HEMIPARESIS. *International Journal of Physiotherapy.* 2016;3(2):147-153.

89. Kara OK, Yardimci BN, Sahin S, Orhan C, Livanelioglu A, Soylu AR. Combined Effects of Mirror Therapy and Exercises on the Upper Extremities in Children with Unilateral Cerebral Palsy: A Randomized Controlled Trial. *Developmental neurorehabilitation.* 2020;23(4):253-264.

90. Narimani A, Kalantari M, Dalvand H, Tabatabaee SM. Effect of mirror therapy on dexterity and hand grasp in children aged 9-14 years with hemiplegic cerebral palsy. *Iranian Journal of Child Neurology.* 2019;13(4):135-142.

91. Cameron D, Craig T, Edwards B, Missiuna C, Schwellnus H, Polatajko HJ. Cognitive Orientation to daily Occupational Performance (CO-OP): A New Approach for Children with Cerebral Palsy. *Phys Occup Ther Pediatr.* 2017;37(2):183-198.

92. Holmström L, Eliasson AC, Almeida R, et al. Efficacy of the small step program in a randomized controlled trial for infants under 12 months old at risk of cerebral palsy (CP) and other neurological disorders. *Journal of Clinical Medicine.* 2019;8(7).

93. Jackman M, Novak I, Lannin N, Froude E, Miller L, Galea C. Effectiveness of Cognitive Orientation to daily Occupational Performance over and above functional hand splints for children with cerebral palsy or brain injury: a randomized controlled trial. *BMC Pediatrics.* 2018;18(1):248.

94. Ko EJ, Sung IY, Moon HJ, Yuk JS, Kim H-S, Lee NH. Effect of Group-Task-Oriented Training on Gross and Fine Motor Function, and Activities of Daily Living in Children with Spastic Cerebral Palsy. *Physical & Occupational Therapy in Pediatrics.* 2020;40(1):18-30.

95. Law MC, Darrah J, Pollock N, et al. Focus on function: a cluster, randomized controlled trial comparing child- versus context-focused intervention for young children with cerebral palsy. *Developmental Medicine & Child Neurology.* 2011;53(7):621-629.

96. Moon J-H, Jung J-H, Hahm S-C, Cho H-y. The effects of task-oriented training on hand dexterity and strength in children with spastic hemiplegic cerebral palsy: A preliminary study. *Journal of physical therapy science.* 2017;29(10):1800-1802.

97. Sousa LK, Brandao MB, Curtin CM, Magalhaes LC. A Collaborative and Cognitive-based Intervention for Young People with Cerebral Palsy. *Canadian Journal of Occupational Therapy - Revue Canadienne d Ergotherapie.* 2020;87(4):319-330.

98. Yuan A, Hou M, Wang S, Liu Q, Li Y, Chen JI. Goals-activity-motor enrichment can improve the motor functioning of infants with a mild to moderate developmental disorder *Chinese Journal of Physical Medicine and Rehabilitation* 2023;12:808-812.

99. Wallen M, O'Flaherty SJ, Waugh MC. Functional outcomes of intramuscular botulinum toxin type a and occupational therapy in the upper limbs of children with cerebral palsy: a randomized controlled trial. *Archives of Physical Medicine & Rehabilitation.* 2007;88(1):1-10.

100. Friel KM, Ferre CL, Brandao M, et al. Improvements in Upper Extremity Function Following Intensive Training Are Independent of Corticospinal Tract Organization in Children With Unilateral Spastic Cerebral Palsy: A Clinical Randomized Trial. *Frontiers in neurology [electronic resource].* 2021;12:660780.

101. Afzal MT, Amjad I, Ghous M. Comparison of classic constraint-induced movement therapy and its modified form on upper extremity motor functions and psychosocial impact in hemiplegic cerebral palsy. *Journal of the Pakistan Medical Association.* 2022;72(7):1418-1421.

102. Bingol H, Gunel MK. Comparing the effects of modified constraint-induced movement therapy and bimanual training in children with hemiplegic cerebral palsy mainstreamed in regular school: A randomized controlled study. *Arch Pediatr.* 2022;29(2):105-115.

103. Dong AQ, Fong NK. Remind to move - A novel treatment on hemiplegic arm functions in children with unilateral cerebral palsy: A randomized cross-over study. *Developmental neurorehabilitation.* 2016;19(5):275-283.

104. Mohamed RA, Yousef AM, Radwan NL, Ibrahim MM. Efficacy of different approaches on quality of upper extremity function, dexterity and grip strength in hemiplegic children: a randomized controlled study. *European Review for Medical & Pharmacological Sciences.* 2021;25(17):5412-5423.

105. Rostami HR, Arastoo AA, Nejad SJ, Mahany MK, Malamiri RA, Goharpey S. Effects of modified constraint-induced movement therapy in virtual environment on upper-limb function in children with spastic hemiparetic cerebral palsy: a randomised controlled trial. *Neurorehabilitation.* 2012;31(4):357-365.
