# Supplementary material for: Active upper‐limb therapies for hand function, individual goal achievement, and self‐care in children with cerebral palsy: A network meta‐analysis
Source: Dev Med Child Neurol. 2025 Sep 5;67(12):1543–53. doi: 10.1111/dmcn.16476 (PMC12618955; doi:10.1111/dmcn.16476)
Supplement: Supplementary file 3 — Table S3: Adverse events, adherence, loss to follow‐up [file DMCN-67-1543-s008.docx]

## Table S3 Adverse events, adherence, loss to follow-up

Table S3. Study details pertaining to adverse events, adherence, loss to follow-up, financial disclosure.

|  | Adverse Event | | Adherence | | Loss to follow-up/drop out | | Financial Disclosure |
| --- | --- | --- | --- | --- | --- | --- | --- |
|  | Intervention | Control | Intervention | Control | Intervention | Control |  |
| ***Action Observation*** | |  |  |  |  |  |  |
| Beani 2023 | 0 | 0 | 100% | NR | 0/15 | 0/15 | Yes |
| Buccino 2012 | NR | NR | NR | NR | 0/8 | 0/7 | NR |
| Buccino 2018 | NR | NR | NR | NR | 0/11 | 0/7 | NR |
| Elbagoury 2022 | NR | NR | NR | NR | NR | NR | NR |
| Kim 2018 | NR | NR | 100% | 100% | 0/6 | 0/6 | NR |
| Kim 2020 | NR | NR | NR | NR | 0/5 | 0/5 | NR |
| Kirkpatrick 2016 | 0 | 0 | Mean play sessions 48.2/60 | Mean play sessions 54.8/60 | 26/35  withdrew 3  LTF 6 | 27/35  withdrew 1  LTF 6  Data error 1 | Yes |
| Palomo-Carrion 2023 | 0 | 0 | 100% adherence, 96% planned dose | 100% adherence, 97.5% planned dose | 1/6 unable to complete final assessment due to surgery | 0/6 | Yes |
| Quadrelli 2019 | NR | NR | NR | NR | 0/4 | 0/4 | Yes |
| Sgandurra 2013 | 0 | 0 | 100 | 100 | 0/12 | 0/12 | Yes |
| Simon-Martinez 2020 | NR | NR | 100 | 100 | 0/22 | 2/22 | Yes |
| **Bimanual** |  |  |  |  |  |  |  |
| Araneda 2022[^4^](#_ENREF_4) | NR | NR | NR | NR | NR | NR | Yes |
| Araneda 2024[^32^](#_ENREF_32) | NR | NR | NR | NR | 1/25 lost at T2 | 1/25 lost at T0  1/25 lost at T2 | Yes |
| Bleyenheuft 2015^26^ | NR | NR | NR | Mean 80 hrs/90 | 1/11 | 0/12 | Yes |
| Bleyenheuft 2017^27^ | NR | NR | 84.5/90 hrs | Mean 2.8 hrs/wk | 0/10 | 2/10  LTF 2 | Yes |
| Brandao 2014^28^ | NR | NR | Camp >90% time in bimanual tasks. HP of goals = 165mins | Camp >90% time in bimanual tasks. HP of goals = 184 mins | Matched pair excluded | 1/11 not have CP so withdrawn | Yes |
| Brandao 2018^29^ | NR | NR | NR | NR | 1/10  1 could not do baseline assessment | 1/10  1 withdrew medical problem | Yes |
| Faccini 2011 & Fedrizzi 2013 | Transient reduction in grasp function of nonaffected hand in the mCIMT group. | NR | NR | NR | mCIMT gained 2 extra participants due to organizational reasons. No dropouts | 10% (n=4) dropout for each of BIM and UC groups. | Yes |
| Ferre 2017^30^ | NR | NR | 82.9/90 hrs | 76.7/90 hrs | 8/20  Did not receive intervention or withdrew | 8/20 | Yes |
| Figueiredo 2020^31^ | 0 | 0 | 87.2 hrs actual vs. 90 hrs intended | 4.5 hrs actual vs. 4.5 hrs intended | 2/21 did not receive intervention due to commuting issues | 0/20 | Yes |
| Gordon 2007^32^ | NR | NR | 10 children spent 25% more time on tasks compared to previous CIMT trial. HP compliance twice as high during intervention and 50% greater after intervention. | NR | 1/10 LTF at 1mth  1/10 AHA video omitted due to poor quality  1/10 unable to do drawer task | 1/10 LTF at 1 mth | Yes |
| Kuo 2016[^21^](#_ENREF_21) | NR | NR | NR | NR | 0/10 | 1/10 drop out | Yes |
| **CIMT** |  |  |  |  |  |  |  |
| Aarts 2010^71^ and  Geerdink 2013^72^ | NR | NR | Mean 9/9 hrs/wk + mean 12.3/? hrs HP | Mean 1.5/1.5 hrs/wk + mean 12.7/? hrs HP | 0/28 | 2/24  2 withdrew | Yes |
| Abd El-Kafy 2014^37^ | NR | NR | NR | NR | 0/15 | 2/15 | NR |
| Afzal 2022 | NR | NR | NR | 1/20 seizures not stated if related to intervention | 2/20 withdrew | 1/20 withdrew due to seizures | Yes |
| Al-Oraibi 2011^36^ | NR | NR | NR | NR | 0/8 | 7/8  1 withdrew | Yes |
| Bingol 2022 | 0 | 0 | Diaries, phone call, whatsapp messages for adherence & compliance | Diaries, phone call, whatsapp messages for adherence & compliance | 0 | 0 |  |
| de Brito Brandao 2010^73^ | Initially “children showed irritability with the use of the restriction” | NR | NR | NR | 0 | 1 dropout due to family reasons | Yes |
| Case-Smith  2012^74^  and Deluca  2012^75^ | “no frustration or discomfort related to continuous casting” | As per inter- vention group | Fidelity (video) and logs documenting activities, challenges etc. | As per intervention group | 0 | 0 | Yes |
| Chamudot 2018^39^ | NR | NR | Mean 48.4/60 hrs | Mean 45/60 hrs | 1/18  Withdrew 1 | 2/18  Ineligible |  |
| Charles 2006^40^ | NR | NR | NR (assume full participation?)  HP: 5.7 hrs during 2 wks Rx (of recommended 10 hrs); 7.3 hrs/wk during 6 mth post Rx /10hrs/wk planned | NR | 8/19 | 3/14 | Yes |
| Chen 2013^41^ and  Chen 2014^42^ and  Hsin 2012^43^ | NR | NR | Mean constraint time 3.5 hrs / 3.5-4 hrs planned | NR | 0/24 | 1/23 | Yes |
| Choudhary 2013^46^ | NR | NR | NR | NR | 1/16 | 0/15 | NR |
| Christmas 2018^44^ | 3 serious AE  1 flu induced wheeze, 1 chest infection, 1 rash  Deemed unrelated  Non serious AE  12  2 bruising, 10 skin abrasions | 1 Serious AE  Seizure  Deemed unrelated | Median hrs 3.6 more than manual restraint group. |  | 1/32  1 missed 10 wk assessment | 3/30  1 withdrew  2 LTF | YES |
| Deppe 2013^76^ | NR | NR | NR | NR | CIMT – 2 dropout. Unsure if is CP group or all children. | BiT – 3 dropout Unsure if is CP group or all children. | Yes |
|  |  |  |  |  |  |  |  |
| Dong 2017a[^17^](#_ENREF_17) | No major AEs. | Remind to move – no AEs. | CIMT -Attendance 91.7% | Remind to move – 100% compliance | CIMT – 2/24 children drop out due to inconvenience of restraint | Remind to move – 0/25 | Yes |
| Dong 2017b[^17^](#_ENREF_17) |  | Conventional rehab – no adverse events |  | Conventional rehab – 95.7% attendance |  | Conventional rehab – 1/24 dropout as parent refused allocation |  |
| Eliasson 2018^50^ | 0 | 0 | Full adherence | Full adherence | 1/19 excluded as had bilateral CP not unilateral CP | 5/18 excluded as not have unilateral CP | Yes |
| Eugster-Buesch 2012^51^ | NR | NR | Splint wear: 72% always or often wore 6hrs/6hrs | NR | 4 dropouts (not clear which group) |  | Yes |
| Facchin 2011^52^ and Fedrizzi 2013^53^ | Transient reduction in grasp function of nonaffected hand in the mCIMT group. | NR | NR | NR | mCIMT gained 2 extra participants due to organizational reasons. No dropout. | 10% (n=4) dropout for each of BIM and usual care groups. | Yes |
| Friel 2021 | 0 | 0 | NR | NR | 3 LTF | 1 withdrew  4 LTF | Yes |
| Gelkop 2015^54^ | 0 | 0 | 100% | 100% | 1/6 not cooperate in QUEST assessment | 1/6 not cooperate in QUEST assessment | No funding reported |
| Gordon 2011 & Brandao 2012 | 0 | 0 | 100% | 100% | 1/22 dropped out after randomization (not sure of group) | 1/22 excluded for inability to comply with testing | Yes |
| Hoare 2013 | 0 | 0 | Mean wear time at home of mitt=98.5 hrs- 59% of expected wear time (168 hrs). Difficulties due to illness, sleep times, usual family, work & social demands. 16 hrs therapy over 8 wks. | Mean hrs at home=31.6. 16 hrs therapy over 8 wks. | 0 | 1 drop out after finding out allocation, but before baseline. | Yes |
| Hwang 2020 ^55^ | 2 children had increased crying in the first wk Feasibility study: 8/12 parents said the mCIMT experience was difficult or very difficult. 10/12 said they would recommend the treatment to other families. | 0 | 90% adherence to protocol. Wear time of splint <6 hrs/day. | NR | No dropout in intervention period. 2 children not participate in study as parents thought child too young | NR | Yes. |
| Kirton 2016^79^  And  Kuo 2018 | 0 | 0 | NR | NR | Gained two participants who were randomized to rTMS were reassigned to sham TMS because of high resting motor thresholds. | 0/12 | Trial registered at Clinicaltrials.gov/ NCT01189058 |
| Klingels 2013^80^ | NR | NR | 9/23 did not reach 80% compliance (40 hr) | 8/25 did not reach 80% compliance (40 hr) | 3 withdrew from CIMT due to behaviour difficulties, commitment, and 1 LTF at final timepoint | 0 | Yes |
| Liang 2023 | 0 | 0 | 100% | 100% | 1/25 withdrew due to botulinum toxin | 1/25 withdrew due to transportation | Yes |
| Lin 2011^57^ | Parent-child dysfunctional interaction score increased, but overall parent stress remained same. | No change in parent stress | NR | NR | 1/11 withdrew | 0 lost | Yes |
| Maitre 2020^81^ | 0 | 0 | Parent intervention fidelity for 4 wks between 93%-99%. | NR | 2 withdrew | 3 withdrew | Yes |
| Maring 2019[^66^](#_ENREF_66) | NR | NR | Total 60hrs planned, actual mean 38 hrs, due to cancellations | Home based group did 33 hrs formal HP activities | 0 | 0 | Yes. |
| Mohamed 2021[^104^](#_ENREF_104) | NR | NR | NR | NR | 0 | 0 | Yes |
| Ostadzadeh 2023[^67^](#_ENREF_67) | NR | NR | NR – parents sent videos | NR- parents sent videos | 1/11 | 2/12 | Yes |
| Paloma-Carrion 2020[^68^](#_ENREF_68) | NR | NR | NR | NR | 0 | 0 | Yes |
| Paloma-Carrion 2021[^69^](#_ENREF_69) | NR | NR | Mean 94/100hrs | Mean 94/100hrs | 0 | 0 | Yes |
| Ramey 2021[^70^](#_ENREF_70)  a) CIMT_60h_cast  b) CIMT_60h_mitt  c) CIMT_30h_cast  d) CIMT_30h_mitt | 4 (not related) | NR | 100% restraint wear  95% dose | NR | 1 LTF  2 withdrew, 2 LTF  2 withdrew, 3 LTF  2 withdrew, 4 LTF | 1 LTF | Yes |
| Rostami 2012a^58 (VR, CIMT etc)^ | NR | NR | NR | NR | 0/8 | 0/8 | Yes |
| Rostami 2012b^59 (home vs clinic)^ | NR | NR | NR | NR | NR | NR | Yes |
| Sakzewski 2011 [^72-74^](#_ENREF_72) | 0 | 0 | 59% 60/60 hrs  41% 54/60 | 58% 60/60  39% 54/60 hrs  3% 42/60 hrs | 3/32  3LTF | 3/32  1 injured prior to baseline  2 LTF | Yes |
| Sakzewski 2015 ^63^ | 1 Mod AE not related | 0 | 56% 45/45 hrs  24% 40-45/45 hrs  12% 35-39/40 hrs  8% < 30/45 hrs | 95% 6/6 sessions  5% 5/6 sessions  Mean 30.9 hrs/36 hrs HP | 4/28  3 withdrew  1 LTF | 19/25  5 withdrew  1 LTF | Yes |
| Sakzewski 2015^84^ | 1/25 had seizure due to fever (unrelated to therapy) | 0 | 56% received 45h; 24% had 40-45hrs, 12% had 35-39hrs, 2 children had<30hrs | OT: 95% had 6 sessions, 1 child had 5 sessions. 68% of children completed average 20.9hrs HP (range 4.5-39.8hrs). | 3 withdrew.  Retention at 13 & 26 wks =96% | 6 withdrew. Retention at 13 and 26 wk =94% and 100%. | Yes |
| Smania 2009 ^64^ | NR | NR | NR | NR | 1 child refused enrolment in mCIMT group due to nervousness | 0 | NR |
| Sung 2005^65^ | Forced use group – no adverse events/decline in function of unaffected /restrained hand observed | NR | NR | NR | nil | “Difficult to recruit participants for control group” Potentially 5/18 dropped from control | NR |
| Taub 2004^48^ and Deluca 2006^47^ | 2/9 behaviour problems  4/9 minor skin irritations from cast | NR | NR | NR | Mean 2.2 hrs/wk | NR | Yes |
| Taub 2011[^81^](#_ENREF_81)^85^ | Children infrequently complained beyond first day. | 0 | NR | NR | 0 | 2 children dropped out prior to intervention (seizure; hospitalisation) | Yes |
| Vaghela 2014^66^ | NR | NR | NR | NR | 0 | 0 | Yes |
| Wallen 2011^67^ | 5/25 exp adverse minor – frustration and refusal to cooperate | 1/25 | Hrs intervention completed 5/25 experienced challenges | Hrs intervention reported – 1/25 experienced challenges | No loss | No loss | Yes |
| Xu 2012^68^ | 0 | 0 | NR | NR | CIMT - 2/23 discontinued | OT group – 1/26 discontinued, 2/26 LTF | Yes |
| Yu 2012^69^ | NR | NR | NR | NR | NR – 4 children in study lost but not specified which groups | NR– 4 children in study LTF but not specified which group | NR |
| Zafer 2016^70^ | NR | NR | NR | NR | 1/10 | 1/10 | Yes. |
| **Mirror** |  |  |  |  |  |  |  |
| Bruchez 2016[^87^](#_ENREF_87) | No adverse events | 0 | NR | NR | 8/45: 2 LTF, 6 discontinued | 6/45: 1 withdrew, 2 LTF, 3 discontinued | Clinical trial protocol registered International Standard RCT registry #48748291 |
| Elsepaee 2016[^88^](#_ENREF_88) | NR | NR | NR | NR | NR | NR | No disclosure stated |
| Gygax 2011[^14^](#_ENREF_14) | NR | NR | Half children reported difficulty concentrating on 3 tasks for 15 mins, but not recorded with group. | Half children reported difficulty concentrating on 3 tasks for 15 mins, but not recorded with group. | 0 | 0 | Yes |
| Kara 2020 ^78^ | 1/17 not cooperative | 0 | 100% | 100% | 2/17  1 withdrew, 1 did not cooperate | 2/17  1 LTF  1 Illness | NR |
| Narimani 2019 [^90^](#_ENREF_90) | NR | NR | Varying concentration | NR | 0 | 0 | Yes |
| **Other** |  |  |  |  |  |  |  |
| Cameron 2017^34^ | NR | NR | Therapists targeted goals 100% time. | Therapists focused on goals 62% of time and 38% time on underlying components | 0/9 | 1/9 LTF at 4 mths | Yes |
| Holmstrom 2019 | 0 | 0 | Parents in Small Step group > satisfaction than control | Parents satisfied with program | 0 | 1 dropout - dissatisfaction with allocation | Yes |
| Jackman 2018^35^ | NR | NR | Mean CO-OP 485/600mins  Mean home practice 372/600 mins | Splint wear adherence 47.1% | 4/15  4 withdrew | 5/15  4 withdrew  1 LTF | Yes |
| Ko 2020[^94^](#_ENREF_94) | NR | NR | NR | NR | 0 | 0  Did not do follow-up for control group. | Yes |
| Law 2011 | NR | NR | 18.7 hrs direct intervention | 17.7 hrs direct intervention | Therapy child focus group: 6 not receive intervention, 2 LTF | Context focused group: 4 not receive intervention, 6 LTF | Yes |
| McLean 2017^82^ | NR | NR | NR | NR | 1 withdrew (time commitment) | 1 dropout (moved) | Yes |
| Moon 2017 | NR | NR | NR | NR | 0 | 0 | NR |
| Novak 2009 (a)[^12^](#_ENREF_12) | 0 | 0 | Good adherence | NR | 0/12 | 0/12 | Yes |
| Novak 2009 (b)[^12^](#_ENREF_12) | 0 |  | (4 wk group) Over adherence – families continued program after 4 wks |  | 1/12 |  | Yes |
| Sousa 2021 | NR | NR | 100% attendance | 100% attendance | 0/6 | 0/6 | Yes |
| Verhaegh 2023 | NR | NR | 93% of planned 28 h performed | 86% of planned 28 h performed. 2 low adherence (<50%) due to hospital admissions | 0/8 | 0/8 | Yes |
| Yuan 2023 | NR | NR | NR | NR | 8/58 lost to economic or illness | 4/48 lost to economic or illness | Yes |
| Wallen 2007^83^ | Illness/rash | 0 | NR | NR | 3 withdrew (2 not satisfied with allocation, 1 unable to obtain intervention) | 4 withdrew as not satisfied with allocation | NR |

Key: NR, not reported; wk, week; hrs, hours; mths, months; CO-OP, Cognitive Orientation to Occupational Performance; QUEST, Quality of Upper Extremity Skills Test; LTF, lost to follow-up; AEs, adverse events; HP, home program; UC, usual care; rTMS, repetitive transcranial magnetic stimulation; CIMT, constraint induced movement therapy; OT, occupational therapy; AHA, Assisting Hand Assessment;

# References

1. Sgandurra G, Ferrari, Adriano, , Cossu G, Guzzetta, Andrea, , Biagi L, Tosetti M, Fogassi L, Cioni G. Upper Limb Children Action-observation Training (UP-CAT): A Randomised Controlled Trial in Hemiplegic Cerebral Palsy. *BMC Neurology.* 2011;11(1).

2. Gordon AM, Schneider JA, Chinnan A, Charles JR. Efficacy of a hand-arm bimanual intensive therapy (HABIT) in children with hemiplegic cerebral palsy: a randomized control trial. *Developmental Medicine & Child Neurology.* 2007;49(11):830-838.

3. Figueiredo PRP, Mancini MC, Feitosa AM, et al. Hand-arm bimanual intensive therapy and daily functioning of children with bilateral cerebral palsy: a randomized controlled trial. *Developmental Medicine and Child Neurology.* 2020.

4. Araneda R, Herman E, Delcour L, et al. Mirror movements after bimanual intensive therapy in children with unilateral cerebral palsy: A randomized controlled trial. *Dev Med Child Neurol.* 2022.

5. Bleyenheuft Y, Ebner-Karestinos D, Surana B, et al. Intensive upper- and lower-extremity training for children with bilateral cerebral palsy: a quasi-randomized trial. *Developmental Medicine & Child Neurology.* 2017;59(6):625-633.

6. Taub E, Miller NE, Novack TA, et al. Technique to improve chronic motor deficit after stroke. . *Arch Phys Med Rehabil.* 1993;74:347-354.

7. Eliasson AC, Krumlinde-Sundholm L, Gordon AM, et al. Guidelines for future research in constraint-induced movement therapy for children with unilateral cerebral palsy: an expert consensus. *Dev Med Child Neurol.* 2014;56(2):125-137.

8. Hoare BJ, Wallen MA, Thorley MN, Jackman ML, Carey LM, Imms C. Constraint-induced movement therapy in children with unilateral cerebral palsy. *Cochrane Database Syst Rev.* 2019;4:CD004149.

9. Gimeno H, Polatajko H. The Cognitive Orientation to daily Occupational Performance approach in childhood-onset disabilities. *Dev Med Child Neurol.* 2025;67(8):977-985.

10. Polatajko HJ, Mandich A. *Enabling occupation in children : the cognitive orientation to daily occupational performance (CO-OP) approach.* Ontario, Ottowa: CAOT Publications ACE.; 2004.

11. Eliasson AC, Shaw K, Berg E, Krumlinde-Sundholm L. An ecological approach of Constraint Induced Movement Therapy for 2-3-year-old children: a randomized control trial. *Research in Developmental Disabilities.* 2011;32(6):2820-2828.

12. Novak I, Cusick A, Lannin N. Occupational therapy home programs for cerebral palsy: double-blind, randomized, controlled trial. *Pediatrics.* 2009;124(4):e606-614.

13. Verhaegh APM, Groen BE, Aarts PBM, et al. Multisensory Stimulation and Priming (MuSSAP) in 4-10 Months Old Infants with a Unilateral Brain Lesion: A Pilot Randomised Clinical Trial. *Occup Ther Int.* 2023;2023:8128407.

14. Gygax MJ, Schneider P, Newman CJ. Mirror therapy in children with hemiplegia: a pilot study. *Developmental Medicine and Child Neurology.* 2011;53(5):473-476.

15. Te Velde A, Morgan C, Finch-Edmondson M, et al. Neurodevelopmental Therapy for Cerebral Palsy: A Meta-analysis. *Pediatrics.* 2022;149(6).

16. Vaughan-Graham J, C. C. Defining a Bobath clinical framework - a modified e-Delphi study. *Physiother Theory Pract.* 2016;32:612-627.

17. Dong VA, Fong KN, Chen YF, Tseng SS, Wong LM. 'Remind-to-move' treatment versus constraint-induced movement therapy for children with hemiplegic cerebral palsy: a randomized controlled trial. *Developmental Medicine & Child Neurology.* 2017;59(2):160-167.

18. McLean B, Taylor S, Blair E, Valentine J, Carey L, Elliott C. Somatosensory Discrimination Intervention Improves Body Position Sense and Motor Performance in Children With Hemiplegic Cerebral Palsy. *American Journal of Occupational Therapy.* 2017;71(3):1-9.

19. Klingels K, Feys H, Molenaers G, et al. Randomized trial of modified constraint-induced movement therapy with and without an intensive therapy program in children with unilateral cerebral palsy. *Neurorehabilitation & Neural Repair.* 2013;27(9):799-807.

20. Simon-Martinez C, Mailleux L, Hoskens J, et al. Randomized controlled trial combining constraint-induced movement therapy and action-observation training in unilateral cerebral palsy: clinical effects and influencing factors of treatment response. *Therapeutic Advances in Neurological Disorders.* 2020;13.

21. Kuo HC, Gordon AM, Henrionnet A, Hautfenne S, Friel KM, Bleyenheuft Y. The effects of intensive bimanual training with and without tactile training on tactile function in children with unilateral spastic cerebral palsy: A pilot study. *Res Dev Disabil.* 2016;49-50:129-139.

22. Beani E, Menici V, Sicola E, et al. Effectiveness of the home-based training program Tele-UPCAT (Tele-monitored UPper Limb Children Action Observation Training) in unilateral cerebral palsy: a randomized controlled trial. *Eur J Phys Rehabil Med.* 2023.

23. Buccino G, Arisi D, Gough P, et al. Improving upper limb motor functions through action observation treatment: a pilot study in children with cerebral palsy. *Developmental Medicine & Child Neurology.* 2012;54(9):822-828.

24. Buccino G, Molinaro A, Ambrosi C, et al. Action Observation Treatment Improves Upper Limb Motor Functions in Children with Cerebral Palsy: A Combined Clinical and Brain Imaging Study. *Neural Plasticity.* 2018;2018:4843985.

25. Elbagoury WS, El-Saeed TM, Olama KA, Kamel MI. Functional-outcomes-of-verbaldirected-training-versus-visualdirected-training-in-children-with-unilateral-cerebral-palsy. *26.* 2022;1:1205-1210.

26. Kim DH, An DH, Yoo WG. Effects of live and video form action observation training on upper limb function in children with hemiparetic cerebral palsy. *Technology & Health Care.* 2018;26(3):437-443.

27. Kim DH. Comparison of short- and long-time action observation training (AOT) on upper limb function in children with cerebral palsy. *Physiotherapy Practice & Research.* 2020;41(1):53-58.

28. Kirkpatrick E, Pearse J, James P, Basu A. Effect of parent-delivered action observation therapy on upper limb function in unilateral cerebral palsy: a randomized controlled trial. *Developmental Medicine & Child Neurology.* 2016;58(10):1049-1056.

29. Palomo-Carrion R, Zuil-Escobar JC, Cabrera-Guerra M, Barreda-Martinez P, Martinez-Cepa CB. Mirror and action observation therapy in children with unilateral spastic cerebral palsy: a feasibility study. *Revista de Neurologia.* 2022;75(11):325-332.

30. Quadrelli E, Anzani A, Ferri M, et al. Electrophysiological correlates of action observation treatment in children with cerebral palsy: A pilot study. *Developmental Neurobiology.* 2019;79(11-12):934-948.

31. Sgandurra G, Ferrari A, Cossu G, Guzzetta A, Fogassi L, Cioni G. Randomized trial of observation and execution of upper extremity actions versus action alone in children with unilateral cerebral palsy. *Neurorehabilitation & Neural Repair.* 2013;27(9):808-815.

32. Araneda R, Ebner-Karestinos D, Paradis J, et al. Changes Induced by Early Hand-Arm Bimanual Intensive Therapy Including Lower Extremities in Young Children With Unilateral Cerebral Palsy: A Randomized Clinical Trial. *JAMA Pediatrics.* 2024;178(1):19-28.

33. Bleyenheuft Y, Arnould C, Brandao MB, Bleyenheuft C, Gordon AM. Hand and Arm Bimanual Intensive Therapy Including Lower Extremity (HABIT-ILE) in Children With Unilateral Spastic Cerebral Palsy: A Randomized Trial. *Neurorehabilitation & Neural Repair.* 2015;29(7):645-657.

34. Brandao MB, Ferre C, Kuo HC, et al. Comparison of Structured Skill and Unstructured Practice During Intensive Bimanual Training in Children With Unilateral Spastic Cerebral Palsy. *Neurorehabilitation & Neural Repair.* 2014;28(5):452-461.

35. Brandao MB, Mancini MC, Ferre CL, et al. Does Dosage Matter? A Pilot Study of Hand-Arm Bimanual Intensive Training (HABIT) Dose and Dosing Schedule in Children with Unilateral Cerebral Palsy. *Physical & Occupational Therapy in Pediatrics.* 2018;38(3):227-242.

36. Facchin P, Rosa-Rizzotto M, Pozza LVD, et al. Multisite Trial Comparing the Efficacy of Constraint-Induced Movement Therapy with that of Bimanual Intensive Training in Children with Hemiplegic Cerebral Palsy. *American Journal of Physical Medicine & Rehabilitation.* 2011;90(7):539-553.

37. Fedrizzi E, Rosa-Rizzotto M, Turconi AC, et al. Unimanual and bimanual intensive training in children with hemiplegic cerebral palsy and persistence in time of hand function improvement: 6-month follow-up results of a multisite clinical trial. *Journal of Child Neurology.* 2013;28(2):161-175.

38. Ferre CL, Brandao M, Surana B, Dew AP, Moreau NG, Gordon AM. Caregiver-directed home-based intensive bimanual training in young children with unilateral spastic cerebral palsy: a randomized trial. *Developmental Medicine & Child Neurology.* 2017;59(5):497-504.

39. Aarts PB, Jongerius PH, Geerdink YA, van Limbeek J, Geurts AC. Effectiveness of modified constraint-induced movement therapy in children with unilateral spastic cerebral palsy: a randomized controlled trial. *Neurorehabilitation & Neural Repair.* 2010;24(6):509-518.

40. Geerdink Y, Aarts P, Geurts AC. Motor learning curve and long-term effectiveness of modified constraint-induced movement therapy in children with unilateral cerebral palsy: a randomized controlled trial. *Research in Developmental Disabilities.* 2013;34(3):923-931.

41. Abd El-Kafy EM, Elshemy SA, Alghamdi MS. Effect of constraint-induced therapy on upper limb functions: a randomized control trial. *Scandinavian Journal of Occupational Therapy.* 2014;21(1):11-23.

42. Al-Oraibi S, Eliasson AC. Implementation of constraint-induced movement therapy for young children with unilateral cerebral palsy in Jordan: a home-based model. *Disability & Rehabilitation.* 2011;33(21-22):2006-2012.

43. de Brito Brandao M, Mancini MC, Vaz DV, Pereira de Melo AP, Fonseca ST. Adapted version of constraint-induced movement therapy promotes functioning in children with cerebral palsy: a randomized controlled trial. *Clinical Rehabilitation.* 2010;24(7):639-647.

44. Case-Smith J, DeLuca SC, Stevenson R, Ramey SL. Multicenter randomized controlled trial of pediatric constraint-induced movement therapy: 6-month follow-up. *American Journal of Occupational Therapy.* 2012;66(1):15-23.

45. DeLuca SC, Case-Smith J, Stevenson R, Ramey SL. Constraint-induced movement therapy (CIMT) for young children with cerebral palsy: effects of therapeutic dosage. *Journal of Pediatric Rehabilitation Medicine.* 2012;5(2):133-142.

46. Chamudot R, Parush S, Rigbi A, Horovitz R, Gross-Tsur V. Effectiveness of Modified Constraint-Induced Movement Therapy Compared With Bimanual Therapy Home Programs for Infants With Hemiplegia: A Randomized Controlled Trial. *American Journal of Occupational Therapy.* 2018;72(6):7206205010p7206205011-7206205010p7206205019.

47. Charles JR, Wolf SL, Schneider JA, Gordon AM. Efficacy of a child-friendly form of constraint-induced movement therapy in hemiplegic cerebral palsy: a randomized control trial. *Developmental Medicine & Child Neurology.* 2006;48(8):635-642.

48. Chen CL, Kang LJ, Hong WH, Chen FC, Chen HC, Wu CY. Effect of therapist-based constraint-induced therapy at home on motor control, motor performance and daily function in children with cerebral palsy: a randomized controlled study. *Clinical Rehabilitation.* 2013;27(3):236-245.

49. Chen HC, Chen CL, Kang LJ, Wu CY, Chen FC, Hong WH. Improvement of upper extremity motor control and function after home-based constraint induced therapy in children with unilateral cerebral palsy: immediate and long-term effects. *Archives of Physical Medicine & Rehabilitation.* 2014;95(8):1423-1432.

50. Hsin YJ, Chen FC, Lin KC, Kang LJ, Chen CL, Chen CY. Efficacy of Constraint-Induced Therapy on Functional Performance and Health-Related Quality of Life for Children With Cerebral Palsy: A Randomized Controlled Trial. *Journal of Child Neurology.* 2012;27(8):992-999.

51. Christmas PM, Sackley C, Feltham MG, Cummins C. A randomized controlled trial to compare two methods of constraint-induced movement therapy to improve functional ability in the affected upper limb in pre-school children with hemiplegic cerebral palsy: CATCH TRIAL. *Clinical Rehabilitation.* 2018;32(7):909-918.

52. Choudhary A, Gulati S, Kabra M, et al. Efficacy of modified constraint induced movement therapy in improving upper limb function in children with hemiplegic cerebral palsy: a randomized controlled trial. *Brain & Development.* 2013;35(9):870-876.

53. Deppe W, Thuemmler K, Fleischer J, Berger C, Meyer S, Wiedemann B. Modified constraint-induced movement therapy versus intensive bimanual training for children with hemiplegia - a randomized controlled trial. *Clinical Rehabilitation.* 2013;27(10):909-920.

54. Eliasson AC, Nordstrand L, Ek L, et al. The effectiveness of Baby-CIMT in infants younger than 12 months with clinical signs of unilateral-cerebral palsy; an explorative study with randomized design. *Research in Developmental Disabilities.* 2018;72:191-201.

55. Eugster-Buesch F, de Bruin ED, Boltshauser E, et al. Forced-use therapy for children with cerebral palsy in the community setting: a single-blinded randomized controlled pilot trial. *Journal of Pediatric Rehabilitation Medicine.* 2012;5(2):65-74.

56. Gelkop N, Burshtein DG, Lahav A, et al. Efficacy of constraint-induced movement therapy and bimanual training in children with hemiplegic cerebral palsy in an educational setting. *Physical & Occupational Therapy in Pediatrics.* 2015;35(1):24-39.

57. Gordon AM, Hung YC, Brandao M, et al. Bimanual training and constraint-induced movement therapy in children with hemiplegic cerebral palsy: a randomized trial. *Neurorehabilitation & Neural Repair.* 2011;25(8):692-702.

58. de Brito Brandao M, Gordon AM, Mancini MC. Functional impact of constraint therapy and bimanual training in children with cerebral palsy: a randomized controlled trial. *American Journal of Occupational Therapy.* 2012;66(6):672-681.

59. Hoare B, Imms C, Villanueva E, Rawicki HB, Matyas T, Carey L. Intensive therapy following upper limb botulinum toxin A injection in young children with unilateral cerebral palsy: a randomized trial. *Developmental Medicine & Child Neurology.* 2013;55(3):238-247.

60. Hwang YS, Kwon JY. Effects of Modified Constraint-Induced Movement Therapy in Real-World Arm Use in Young Children with Unilateral Cerebral Palsy: A Single-Blind Randomized Trial. *Neuropediatrics.* 2020;51(4):259-266.

61. Kirton A, Andersen J, Herrero M, et al. Brain stimulation and constraint for perinatal stroke hemiparesis: The PLASTIC CHAMPS Trial. *Neurology.* 2016;86(18):1659-1667.

62. Kuo HC, Zewdie E, Ciechanski P, Damji O, Kirton A. Intervention-Induced Motor Cortex Plasticity in Hemiparetic Children With Perinatal Stroke. *Neurorehabil Neural Repair.* 2018;32(11):941-952.

63. Liang KJ, Chen HL, Huang CW, Wang TN. Efficacy of Constraint-Induced Movement Therapy Versus Bimanual Intensive Training on Motor and Psychosocial Outcomes in Children With Unilateral Cerebral Palsy: A Randomized Trial. *Am J Occup Ther.* 2023;77(4).

64. Lin KC, Wang TN, Wu CY, et al. Effects of home-based constraint-induced therapy versus dose-matched control intervention on functional outcomes and caregiver well-being in children with cerebral palsy. *Research in Developmental Disabilities.* 2011;32(5):1483-1491.

65. Maitre NL, Jeanvoine A, Yoder PJ, et al. Kinematic and Somatosensory Gains in Infants with Cerebral Palsy After a Multi-Component Upper-Extremity Intervention: A Randomized Controlled Trial. *Brain Topography.* 2020.

66. Maring J, Wentzell E. Constraint Induced Movement Therapy: Impact of Setting on Outcomes. *Journal of Allied Health.* 2019;48(3):e73-e77.

67. Ostadzadeh A, Amini M, Hassani Mehraban A, Maroufizadeh S, Farajzadeh A. The Effect of Occupation-Based Modified Constraint-Induced Movement Therapy on the Participation of Children with Cerebral Palsy: A Single-Blind Randomized Controlled Trial. *Iran J Child Neurol.* 2023;17(2):39-54.

68. Palomo-Carrion R, Pinero-Pinto E, Ando-LaFuente S, Ferri-Morales A, Bravo-Esteban E, Romay-Barrero H. Unimanual Intensive Therapy with or without Unaffected Hand Containment in Children with Hemiplegia. A Randomized Controlled Pilot Study. *Journal of Clinical Medicine.* 2020;9(9):14.

69. Palomo-Carrion R, Lirio-Romero C, Ferri-Morales A, Jovellar-Isiegas P, Cortes-Vega MD, Romay-Barrero H. Combined intensive therapies at home in spastic unilateral cerebral palsy with high bimanual functional performance. What do they offer? A comparative randomised clinical trial. *Therapeutic Advances in Chronic Disease.* 2021;12:20406223211034996.

70. Ramey SL, DeLuca SC, Stevenson RD, Conaway M, Darragh AR, Lo W. Constraint-induced movement therapy for cerebral palsy: A randomized trial. *Pediatrics.* 2021;148(5).

71. Rostami HR, Malamiri RA. Effect of treatment environment on modified constraint-induced movement therapy results in children with spastic hemiplegic cerebral palsy: a randomized controlled trial. *Disability & Rehabilitation.* 2012;34(1):40-44.

72. Sakzewski L, Ziviani J, Abbott DF, Macdonell RAL, Jackson GD, Boyd RN. Randomized trial of constraint-induced movement therapy and bimanual training on activity outcomes for children with congenital hemiplegia. *Developmental Medicine and Child Neurology.* 2011;53(4):313-320.

73. Sakzewski L, Ziviani J, Abbott DF, Macdonell RA, Jackson GD, Boyd RN. Equivalent retention of gains at 1 year after training with constraint-induced or bimanual therapy in children with unilateral cerebral palsy. *Neurorehabilitation & Neural Repair.* 2011;25(7):664-671.

74. Sakzewski L, Ziviani J, Abbott DF, Macdonell RA, Jackson GD, Boyd RN. Participation Outcomes in a Randomized Trial of 2 Models of Upper-Limb Rehabilitation for Children With Congenital Hemiplegia. *Archives of Physical Medicine & Rehabilitation.* 2011;92(4):531-539.

75. Sakzewski L, Provan K, Ziviani J, Boyd RN. Comparison of dosage of intensive upper limb therapy for children with unilateral cerebral palsy: how big should the therapy pill be? *Research in Developmental Disabilities.* 2015;37:9-16.

76. Sakzewski L, Miller L, Ziviani J, et al. Randomized comparison trial of density and context of upper limb intensive group versus individualized occupational therapy for children with unilateral cerebral palsy. *Developmental Medicine & Child Neurology.* 2015;57(6):539-547.

77. Smania N, Aglioti SM, Cosentino A, et al. A modified constraint-induced movement therapy (CIT) program improves paretic arm use and function in children with cerebral palsy. *European journal of physical & rehabilitation medicine.* 2009;45(4):493-500.

78. Sung IY, Ryu JS, Pyun SB, Yoo SD, Song WH, Park MJ. Efficacy of forced-use therapy in hemiplegic cerebral palsy. *Archives of Physical Medicine & Rehabilitation.* 2005;86(11):2195-2198.

79. Taub E, Ramey SL, DeLuca S, Echols K. Efficacy of constraint-induced movement therapy for children with cerebral palsy with asymmetric motor impairment. *Pediatrics.* 2004;113(2):305-312.

80. Deluca SC, Echols K, Law CR, Ramey SL. Intensive pediatric constraint-induced therapy for children with cerebral palsy: randomized, controlled, crossover trial. *Journal of Child Neurology.* 2006;21(11):931-938.

81. Taub E, Griffin A, Uswatte G, Gammons K, Nick J, Law CR. Treatment of Congenital Hemiparesis With Pediatric Constraint-Induced Movement Therapy. *Journal of Child Neurology.* 2011;26(9):1163-1173.

82. Vaghela VG. To Study the effects of Mcimt Versus Cimt for Young Children with Spastic Hemiplegic Cerebral Palsy-- A Comparitive Study. *Indian Journal of Physiotherapy & Occupational Therapy.* 2014;8(2):136-141.

83. Wallen M, Ziviani J, Naylor O, Evans R, Novak I, Herbert RD. Modified constraint-induced therapy for children with hemiplegic cerebral palsy: a randomized trial. *Developmental Medicine & Child Neurology.* 2011;53(12):1091-1099.

84. Xu K, Wang L, Mai J, He L. Efficacy of constraint-induced movement therapy and electrical stimulation on hand function of children with hemiplegic cerebral palsy: a controlled clinical trial. *Disability & Rehabilitation.* 2012;34(4):337-346.

85. Yu J, Kang H, Jung J. Effects of modified constraint-induced movement therapy on hand dexterity, grip strength and activities of daily living of children with cerebral palsy: a randomized control trial. *Journal of physical therapy science.* 2012;24(10):1029‐1031.

86. Zafer H, Amjad I, Malik AN, Shaukat E. Effectiveness of constraint induced movement therapy as compared to bimanual therapy in upper motor function outcome in child with hemiplegic cerebral palsy. *Pakistan Journal of Medical Sciences.* 2016;32(1):181-184.

87. Bruchez R, Gygax MJ, Roches S, et al. Mirror therapy in children with hemiparesis: a randomized observer-blinded trial. *Developmental Medicine and Child Neurology.* 2016;58(9):970-978.

88. Elsepaee MI, Elhadidy EI, Emara HA, Nawar EAE. EFFECT OF MIRROR VISUAL FEEDBACK ON HAND FUNCTIONS IN CHILDREN WITH HEMIPARESIS. *International Journal of Physiotherapy.* 2016;3(2):147-153.

89. Kara OK, Yardimci BN, Sahin S, Orhan C, Livanelioglu A, Soylu AR. Combined Effects of Mirror Therapy and Exercises on the Upper Extremities in Children with Unilateral Cerebral Palsy: A Randomized Controlled Trial. *Developmental neurorehabilitation.* 2020;23(4):253-264.

90. Narimani A, Kalantari M, Dalvand H, Tabatabaee SM. Effect of mirror therapy on dexterity and hand grasp in children aged 9-14 years with hemiplegic cerebral palsy. *Iranian Journal of Child Neurology.* 2019;13(4):135-142.

91. Cameron D, Craig T, Edwards B, Missiuna C, Schwellnus H, Polatajko HJ. Cognitive Orientation to daily Occupational Performance (CO-OP): A New Approach for Children with Cerebral Palsy. *Phys Occup Ther Pediatr.* 2017;37(2):183-198.

92. Holmström L, Eliasson AC, Almeida R, et al. Efficacy of the small step program in a randomized controlled trial for infants under 12 months old at risk of cerebral palsy (CP) and other neurological disorders. *Journal of Clinical Medicine.* 2019;8(7).

93. Jackman M, Novak I, Lannin N, Froude E, Miller L, Galea C. Effectiveness of Cognitive Orientation to daily Occupational Performance over and above functional hand splints for children with cerebral palsy or brain injury: a randomized controlled trial. *BMC Pediatrics.* 2018;18(1):248.

94. Ko EJ, Sung IY, Moon HJ, Yuk JS, Kim H-S, Lee NH. Effect of Group-Task-Oriented Training on Gross and Fine Motor Function, and Activities of Daily Living in Children with Spastic Cerebral Palsy. *Physical & Occupational Therapy in Pediatrics.* 2020;40(1):18-30.

95. Law MC, Darrah J, Pollock N, et al. Focus on function: a cluster, randomized controlled trial comparing child- versus context-focused intervention for young children with cerebral palsy. *Developmental Medicine & Child Neurology.* 2011;53(7):621-629.

96. Moon J-H, Jung J-H, Hahm S-C, Cho H-y. The effects of task-oriented training on hand dexterity and strength in children with spastic hemiplegic cerebral palsy: A preliminary study. *Journal of physical therapy science.* 2017;29(10):1800-1802.

97. Sousa LK, Brandao MB, Curtin CM, Magalhaes LC. A Collaborative and Cognitive-based Intervention for Young People with Cerebral Palsy. *Canadian Journal of Occupational Therapy - Revue Canadienne d Ergotherapie.* 2020;87(4):319-330.

98. Yuan A, Hou M, Wang S, Liu Q, Li Y, Chen JI. Goals-activity-motor enrichment can improve the motor functioning of infants with a mild to moderate developmental disorder *Chinese Journal of Physical Medicine and Rehabilitation* 2023;12:808-812.

99. Wallen M, O'Flaherty SJ, Waugh MC. Functional outcomes of intramuscular botulinum toxin type a and occupational therapy in the upper limbs of children with cerebral palsy: a randomized controlled trial. *Archives of Physical Medicine & Rehabilitation.* 2007;88(1):1-10.

100. Friel KM, Ferre CL, Brandao M, et al. Improvements in Upper Extremity Function Following Intensive Training Are Independent of Corticospinal Tract Organization in Children With Unilateral Spastic Cerebral Palsy: A Clinical Randomized Trial. *Frontiers in neurology [electronic resource].* 2021;12:660780.

101. Afzal MT, Amjad I, Ghous M. Comparison of classic constraint-induced movement therapy and its modified form on upper extremity motor functions and psychosocial impact in hemiplegic cerebral palsy. *Journal of the Pakistan Medical Association.* 2022;72(7):1418-1421.

102. Bingol H, Gunel MK. Comparing the effects of modified constraint-induced movement therapy and bimanual training in children with hemiplegic cerebral palsy mainstreamed in regular school: A randomized controlled study. *Arch Pediatr.* 2022;29(2):105-115.

103. Dong AQ, Fong NK. Remind to move - A novel treatment on hemiplegic arm functions in children with unilateral cerebral palsy: A randomized cross-over study. *Developmental neurorehabilitation.* 2016;19(5):275-283.

104. Mohamed RA, Yousef AM, Radwan NL, Ibrahim MM. Efficacy of different approaches on quality of upper extremity function, dexterity and grip strength in hemiplegic children: a randomized controlled study. *European Review for Medical & Pharmacological Sciences.* 2021;25(17):5412-5423.

105. Rostami HR, Arastoo AA, Nejad SJ, Mahany MK, Malamiri RA, Goharpey S. Effects of modified constraint-induced movement therapy in virtual environment on upper-limb function in children with spastic hemiparetic cerebral palsy: a randomised controlled trial. *Neurorehabilitation.* 2012;31(4):357-365.
