# Supplementary material for: Active upper‐limb therapies for hand function, individual goal achievement, and self‐care in children with cerebral palsy: A network meta‐analysis
Source: Dev Med Child Neurol. 2025 Sep 5;67(12):1543–53. doi: 10.1111/dmcn.16476 (PMC12618955; doi:10.1111/dmcn.16476)
Supplement: Supplementary file 5 — Appendix S1: Outcome measures and search strategy [file DMCN-67-1543-s006.docx]

# Appendix S1

## S1.1 Outcome measures – included and excluded

The following outcome measures were eligible for inclusion: Hand Assessment for Infants (HAI), mini-Assisting Hand Assessment (mini-AHA), Kids-AHA, Both Hands Assessment (BoHA), ABILHAND-Kids, Bayley Scales of Infant and Toddler Development–version III (BSITD-III), Box and Blocks test, Besta Scale, Children’s Hand-use Experience Questionnaire (CHEQ), Jebsen-Taylor Test of Hand Function (JTTHF), Melbourne Assessment of Unilateral Upper Limb Function (MUUL), Melbourne Assessment 2 (MA2), Peabody Developmental Motor Scales-II (PDMS-II), Pediatric Arm Function Test (PAFT), Pediatric Motor Activity Log - Revised (PMAL-R), Quality of Upper Extremity Skills Test (QUEST) domains of Dissociated Movements and Grasps, Shriner’s Hospital Upper Extremity Evaluation (SHUEE), The Tyneside Pegboard Test, PEDI, PEDI-CAT, WeeFIM, COPM, GAS, revised Video-Observation Aarts and Aarts module: Determine Developmental Disregard (VOAA-DDD-R).

The following outcome measures were ineligible as they lacked evidence for validity or reliability for children with CP: 9 hole peg test, Birmingham Bimanual Questionnaire, Bruininks-Oseretsky Test of Motor Proficiency, Caregiver Functional Use Test, Child Arm Use Test, Erhardt Developmental Test, Inventory of New Motor Behaviors, Peabody Developmental Motor Scales I, Pediatric Motor Activity Log (PMAL), QUEST total score/protective extension domain/weightbearing domain/adapted versions, Toddler Arm Use Test, Upper Extremity Function Test, Use test Accelerometry, Grip & pinch strength, Modified Ashworth Scale, Modified Tardieu Scale, Passive range of motion, Strength tests – Box Task & Cup Task.

## S1.2 Categorisation of interventions

**Definitions used for categorising treatment/intervention groups.**

Study interventions were ‘lumped’ together into categories according to their key characteristic. Categories are given below. Each category becomes a node in the network map. For studies which were a combination of 2 key characteristics, these were made into a category to represent a node on the network map. Studies vary in the level of description of detail. Two authors (AB & LS) classified intervention categories. When consensus could not be reached, a third author (MT) was brought in to reach decision.

**Action Observation (AO)**

Action Observation is defined by observation of an action, followed by imitation of the action. AO is based on the discovery of mirror neurons which discharge when a goal directed action is performed and discharge when the goal directed action is observed. AO in children with CP was first described by Sgandurra et al in 2011.[^1^](#_ENREF_1) Studies which report observation of a goal directed action on a video, or by observation of a ‘live’ person were both considered under the umbrella of AO.

**Bimanual Therapy (BiM)**

Bimanual intensive therapy (BiM) is defined by two key components: use of two hands together and intensive, structured upper limb therapy which focuses on improving coordination of the two hands together. Bimanual intensive therapy was first described by Andrew Gordon in 2007.[^2^](#_ENREF_2) Studies which reported a combination of approaches (Hybrid BiM) were classified as BiM when the proportion of BiM was greater than the other intervention approach it was combined with. Examples of interventions and studies which were categorised as Bimanual Therapy include Hand Arm Bimanual Intensive Therapy (HABIT)[^3^](#_ENREF_3) and HABIT-Including Lower Extremity (HABIT-ILE).[^4^](#_ENREF_4)^,^[^5^](#_ENREF_5)

**modified Constraint Induced Movement Therapy (mCIMT)**

CIMT is defined by two key components: restraint of the less affected upper limb, and intensive, structured, upper-limb therapy of the most affected hand. Signature CIMT, first described by Edward Taub in 1993,[^6^](#_ENREF_6) has been adapted and modified in the intervening years. The umbrella definition of CIMT drawn from published guidelines and the definitions of types of CIMT was used.[^7^](#_ENREF_7) Signature CIMT, modified CIMT, hybrid CIMT and forced use therapy were included under the umbrella of CIMT as per previous Cochrane systematic review.[^8^](#_ENREF_8) Types of restraints may include long casts, mitts, splints. Restraints may be applied for a short period or for 24 h/day over a period of weeks. Hybrid CIMT was classified as CIMT when the proportion of CIMT was greater than that of BiM (or other therapy approach) with which it was combined.

**Cognitive Orientation to daily Occupational Performance (CO-OP)**

CO-OP is a problem-solving approach that uses cognitive strategies to facilitate skill acquisition.[^9^](#_ENREF_9) It focuses on the learning of a global problem-solving strategy. CO-OP was first described by Polatajko and Mandich in 2004.[^10^](#_ENREF_10)

**Control**

Control groups were classified as such when a placebo was received which was not an active intervention approach, or, when no treatment was received in the trial (although participants could receive their usual care). For example, groups were classified as ‘control’ when they involved passive intervention (e.g., massage was categorised as control in Eliasson et al., 2011[^11^](#_ENREF_11)).

**Goal directed**

The key factor used to categorise an intervention as goal directed was that the intervention was focused on achieving parent/child identified goals. Intervention activities varied but included motor training, environmental modification and practice of goal activities. Intervention could be clinician delivered, supervised, or delivered via a formal home programme.

Home programs are used as a therapeutic strategy.[^12^](#_ENREF_12) Home programs are considered a method of delivery of and a key ingredient of GDT. Home programs may use multimodal interventions to target problems (activity/participation/body structure) which are identified by parents and therapist collaboratively.[^12^](#_ENREF_12) Home programs are individualised and family-friendly and designed to meet the family goals.[^12^](#_ENREF_12)

**Home-based Early Interventions (HEI)**

Home-based Early Interventions are those interventions specifically for infants and toddlers which include a combination of intervention elements namely: home based, parent-coaching, goal-directed, unimanual training, bimanual training, sensory-motor enrichment. For example, the intervention described in Verhaegh et al., 2023[^13^](#_ENREF_13) called Early Intensive-Upper Limb was considered a Home-based intervention with video coaching of parents. Goals are set by parents together with therapist.

**Mirror therapy**

Mirror therapy involves the use of a mirror to provide a visual illusion of a functional limb to enhance reorganisation and stimulate plasticity of the premotor cortex for adults and children with hemiplegia.[^14^](#_ENREF_14) Mirror therapy was first described by Ramachandran in 1995 in the treatment of phantom pain in amputees, and later found to be a potential rehabilitation intervention in hemiplegia.[^14^](#_ENREF_14)

**Neurodevelopmental Therapy**

Neurodevelopmental Therapy is defined on three key principles: movement analysis of task performance, interdependence of posture and movement and importance of sensory information in motor control.[^15^](#_ENREF_15)^,^[^16^](#_ENREF_16) NDT is characterised by therapist-controlled movement and focused on normalising motor patterns.[^15^](#_ENREF_15)

**Sensory Stimulation Reminder**

Sensory input provided through wristband which is worn by children on the affected upper limb. The wristband provides sensory stimulation using tactile vibration alone, or with other multisensory input (visual lights, auditory song). The wristband aims to increase attention for the affected upper limb, remind the infant/child to move and increase active movement during task activities. Study examples of SSR are Dong 2017,[^17^](#_ENREF_17) Verhaegh 2023.[^13^](#_ENREF_13)

***Categories which combined two intervention modalities:***

**Sensory & GDT (Sens+GDT)**

This intervention was used in McLean et al., 2017.[^18^](#_ENREF_18) Sensory+GDT is an occupation-based approach combined with somatosensory training. Functional goals were made with families, goals were practiced (whole and component), together with active exploration of relevant task item (with vision occluded performed), feedback on performance, repetition of tasks, progression from easy to more complex. Somatosensory training refers to stereognosis, proprioception and two point/touch discrimination training, which was done within an occupation-based framework. where goal performance was the primary outcome. Activities which were meaningful to the child were practised and included active exploration of familiar objects with vision occluded. Repetition and progression from easy to more complex tasks, feedback on performance, calibration with the less affected hand were performed. Sensory+GDT was performed in community settings (home/school) with the caregiver present; caregiver education was included.

**mCIMT & Intensive strengthening (mCIMT+Int)**

Home based mCIMT (with caregiver doing graded activities on goals, supervised by therapist) combined with intensive therapy in the clinic focused on distal upper limb strengthening exercises and bimanual functional activities using principles of repetitive whole-task practice, practice specificity, feedback and grading, including specific practice of two bimanual activities of daily living. Functional activities were chosen by parent and child. This intervention was used in Klingels et al, 2013.[^19^](#_ENREF_19)

**Action Observation & mCIMT (AO+mCIMT)**

This intervention classification involves the combination of mCIMT and AO. The intervention was used in Simon-Martinez et al., 2020.[^20^](#_ENREF_20)

**HEI & SSR (HEI+SSR)**

Home based early intervention (HEI) is combined with Sensory Stimulation Reminder (SSR) which aimed to increase infant attention for affected upper limb and facilitate readiness for goal-directed movement. HEIR+SSR was used in Verhaegh et al., 2023.[^13^](#_ENREF_13)

**Examples of intervention classification:**

Kuo et al., 2016[^21^](#_ENREF_21) - treatment groups were 82h HABIT+8h tactile training (with tactile stimulating materials), and, 82h HABIT + 8h play with tactile stimulating materials (without training). Both interventions were classified as BiM as that was the major component of both intervention arms, and as such it was regarded as a dosage study.

Novak et al., 2009[^12^](#_ENREF_12) – Home program – intervention arm classified as GDT – as key ingredient is GDT and home program is method of delivery.

## S1.3 Full Search Strategy

**MEDLINE**

((Cerebral palsy/ OR cerebral pals$.tw. OR little$ disease.tw. OR CP.tw. OR (unilateral adj3 spastic$).tw. OR (hemiplegi$ adj3 spastic$).tw. OR (diplegi$ adj3 spastic$).tw. OR (triplegi$ adj3 spastic$).tw. OR ((bilateral or bi-lateral) adj3 spastic$).tw. OR (quadripleg$ adj3 spastic$).tw.) OR (dystoni$ adj3 cerebral palsy))

**AND**
**(**
(Therap$.tw. OR therapy.tw. OR Intervention.tw. OR Exercis$.tw. OR Habilitation.tw. OR Training.tw. OR Service$.tw. OR Treat$.tw. OR modalit$.tw. OR exp *Rehabilitation/ OR exp Therapeutics/ OR exp "Physical and Rehabilitation Medicine"/)

**OR**

(bobath$.tw OR neurodevelopment$.tw. OR neuro-developmen.tw. OR neurophysiological.tw. OR neuro-physiological.tw. OR neuromuscular.tw. OR

neuro-muscular.tw. OR NDT.tw. OR neurofacilitation$ OR neuro-facilitation$.tw. OR neuro$facilitation.tw.)

**OR**

(Virtual reality exposure therapy/ OR (virtual OR virtually OR VR).tw. OR Exp user computer interface/ OR Exp computer simulation/ OR computer simulat$.tw. OR ((simulat$ OR augment$ OR mediat$) adj3 (world$ OR realit$ OR environment$)).tw. OR Exp video games/ OR (videogame$ OR ((video OR computer OR electronic OR online OR on-line OR simulation OR role playing) adj game$)).tw. OR Wii.tw OR ((head OR helmet) adj mounted).tw. OR (immerse$ OR spatial presence OR lifelike OR life-like).tw. OR interactive$.tw. OR augment$.tw. OR computer$.tw. OR serious gaming.tw. OR software.tw. OR user-computer interface$.tw. OR exergam$.tw. OR reality system$.tw. OR (Nintendo OR sony OR gestureTek OR NeuroVR OR Hocoma OR Motek OR virtual realities OR haptic Master OR Microsoft OR Xbox OR essential reality OR SensAble OR Novint OR Cyberglove).tw. )

OR

(web-based.tw. OR telerehabilitation.tw. OR teletherapy.tw. OR exp teletherapy/ OR exp telerehabilitation/ )

**OR**

(constraint induced movement therapy.tw. OR (constrain$ adj10 (movement$ or therap$)).tw. OR CIMT.tw. OR mCIMT.tw. OR CI therap$.tw. OR forced.tw. OR massed practice.tw.)

**OR**

(bimanual therapy.tw. OR bimanual training.tw. OR Hand arm bimanual intensive training.tw. OR HABIT.tw. OR HABIT-ILE.tw.)

**OR**

(functional therap**$**.tw. OR task oriented training.tw. OR task oriented training program.tw. OR task oriented.tw. OR goal directed training.tw. OR motor training.tw. OR task training.tw. OR task practice.tw. OR task-specific training.tw. OR physiotherap**$**.tw. OR physical therap**$**.tw. OR occupational therap**$**.tw. OR functional therapy.tw. OR task OR OT)

**OR**

(COOP.tw. OR cognitive orientation to occupational performance.tw. OR CO-OP.tw.)

**OR**

(context focussed.tw.)

**OR**

(action observation.tw. OR action observation training.tw. OR action observation therapy.tw. OR action observation treatment.tw.)

**OR**

(mirror therapy.tw. OR mirror therapy intervention.tw. OR mirror therapy program.tw. OR mirror box therapy.tw. OR mirror$.tw.)

OR

(Home active$.tw. OR Home-based.tw. OR Home.tw.)

**)**

**AND**

(exp "upper extremity"/ OR upper extremit$.tw. OR upper limb$.tw. OR hand.tw. OR hands.tw. OR arm.tw. OR arms.tw. OR manual.tw. OR bimanual.tw. OR Elbow.tw. OR elbows.tw. OR Forearm.tw. OR forearms.tw. OR Finger.tw. OR Fingers.tw. OR Thumb.tw. OR thumbs.tw. OR fine motor$.tw.)

**AND**
(
((randomized controlled trial or controlled clinical trial).pt.
 OR randomized.tw. OR randomised.tw. or placebo.tw. or drug therapy.tw. or randomly.tw. or trial.tw. or groups.tw. OR exp 'clinical trial'/ OR exp 'randomized controlled trial'/ OR exp 'placebo'/ OR exp 'quasi experimental study'/ OR exp 'controlled study'/ OR exp 'experimental study'/ OR waitlist-controlled) not (exp animals/ not humans.sh.)
)

**EMBASE**

('cerebral palsy'/exp OR 'cerebral pals*':ti,ab OR 'little* disease':ti,ab OR CP:ti,ab OR (unilateral NEAR/3 spastic*):ti,ab OR (hemiplegi* NEAR/3 spastic*):ti,ab OR (diplegi* NEAR/3 spastic*):ti,ab OR (triplegi* NEAR/3 spastic*):ti,ab OR ((bilateral or bi-lateral) NEAR/3 spastic*):ti,ab OR (quadripleg* NEAR/3 spastic*):ti,ab OR (dystoni* NEAR/3 'cerebral palsy'):ti,ab)

**AND**
**(**
(Rehabilitation/exp OR ‘therapy’/exp OR Rehabilitation:ti,ab OR Therap*:ti,ab OR therapy:ti,ab OR Intervention:ti,ab OR Exercis*:ti,ab OR Habilitation:ti,ab OR Training:ti,ab OR Service*:ti,ab OR Treat*:ti,ab OR modalit*:ti,ab)

OR
('Physical medicine'/exp OR 'Physical therapy modalities'/exp)

OR

(bobath*:ti,ab OR neurodevelopment*:ti,ab OR neuro-developmen:ti,ab OR neurophysiological:ti,ab OR neuro-physiological:ti,ab OR neuromuscular:ti,ab OR neuro-muscular:ti,ab OR NDT:ti,ab OR neurofacilitation* OR neuro-facilitation*:ti,ab OR neuro*facilitation:ti,ab )

OR
('virtual reality'/exp OR 'virtual reality head mounted display'/exp OR 'virtual reality exposure therapy'/exp OR (virtual OR virtually OR VR):ti,ab OR ‘computer interface’/exp OR ‘computer simulation’/exp OR 'computer simulat*':ti,ab OR ((simulat* OR augment* OR mediat*) NEAR/3 (world* OR realit* OR environment*)):ti,ab OR 'video game'/exp OR videogame* OR ((video OR computer OR electronic OR online OR on-line OR simulation OR 'role playing') NEAR/1 game*):ti,ab OR Wii.tw OR ((head OR helmet) NEAR/1 mounted):ti,ab OR (immerse* OR 'spatial presence' OR lifelike OR life-like):ti,ab OR interactive*:ti,ab OR augment*:ti,ab OR computer*:ti,ab OR 'serious gaming':ti,ab OR softwar:ti,ab OR 'user-computer interface*':ti,ab OR exergam*:ti,ab OR 'reality system*':ti,ab OR (Nintendo OR sony OR gestureTek OR NeuroVR OR Hocoma OR Motek OR 'virtual realities' OR haptic Master OR Microsoft OR Xbox OR 'essential reality' OR SensAble OR Novint OR Cyberglove):ti,ab )

**OR**

(web-based:ti,ab OR telehealth/exp OR telerehabilitation/exp OR telemedicine/exp)

**OR**

('constraint induced movement therapy':ti,ab OR (constrain* NEAR/10 (movement* or therap*)):ti,ab OR CIMT:ti,ab OR mCIMT:ti,ab OR 'CI therap*':ti,ab OR forced:ti,ab OR 'massed practice':ti,ab)

**OR**

('bimanual therapy':ti,ab OR 'bimanual training':ti,ab OR 'Hand arm bimanual intensive training':ti,ab OR HABIT:ti,ab OR HABIT-ILE:ti,ab)

**OR**

('functional therap***'**:ti,ab OR 'task oriented training':ti,ab OR 'task oriented training program':ti,ab OR 'task oriented':ti,ab OR 'goal directed training':ti,ab OR 'motor training':ti,ab OR 'task training':ti,ab OR 'task practice':ti,ab OR 'task-specific training':ti,ab OR physiotherap*****:ti,ab OR 'physical therap***'**:ti,ab OR 'occupational therap***'**:ti,ab OR 'functional therapy':ti,ab OR task:ti,ab OR OT:ti,ab)

**OR**

(COOP:ti,ab OR 'cognitive orientation to occupational performance':ti,ab OR CO-OP:ti,ab)

**OR**

('context focussed':ti,ab)

**OR**

('action observation':ti,ab OR 'action observation training':ti,ab OR 'action observation therapy':ti,ab OR 'action observation treatment':ti,ab)

**OR**

('mirror therapy':ti,ab OR 'mirror therapy intervention':ti,ab OR 'mirror therapy program':ti,ab OR 'mirror box therapy':ti,ab OR mirror*:ti,ab)

**OR**

('Home active*':ti,ab OR Home-based:ti,ab OR Home:ti,ab)

**)**

**AND**

('upper limb'/exp OR 'upper extremit*':ti,ab OR 'upper limb*':ti,ab OR hand:ti,ab OR hands:ti,ab OR arm:ti,ab OR arms:ti,ab OR manual:ti,ab OR bimanual:ti,ab OR Elbow:ti,ab OR elbows:ti,ab OR Forearm:ti,ab OR forearms:ti,ab OR Finger:ti,ab OR Fingers:ti,ab OR Thumb:ti,ab OR thumbs:ti,ab OR 'fine motor*':ti,ab)

**AND**

('crossover procedure':exp OR 'double-blind procedure':exp OR 'randomized controlled trial'/exp OR ‘controlled clinical trial’/exp OR 'single-blind procedure':exp OR ‘crossover procedure’/exp OR (random* OR factorial* OR crossover* OR cross NEXT/1 over* OR placebo* OR doubl* NEAR/1 blind* OR singl* NEAR/1 blind* OR assign* OR allocat* OR volunteer*):ab,ti NOT ('animal'/exp NOT ('animal'/exp AND 'human'/exp)))
**AND**
[embase]/lim

**Cochrane**

([mh "Cerebral palsy"] OR "cerebral pals*":ti,ab OR "little* disease":ti,ab OR CP:ti,ab OR (unilateral NEAR/3 spastic*):ti,ab OR (hemiplegi*

NEAR/3 spastic*):ti,ab OR (diplegi* NEAR/3 spastic* ):ti,ab OR (triplegi* NEAR/3 spastic*):ti,ab OR ((bilateral OR bi*) NEAR/3 spastic*):ti,ab OR (quadripleg* NEAR/3 spastic*):ti,ab OR (dystoni* NEAR/3 "cerebral palsy"):ti,ab)

**AND**

(

(Therap*:ti,ab OR therapy:ti,ab OR Intervention OR Exercis* OR Habilitation OR Training OR Service* OR Treat* OR modalit* OR [mh Rehabilitation] OR [mh Therapeutics] OR [mh "Physical and rehabilitation medicine"])

OR

([mh "Physical medicine"] OR [mh "Physical therapy modalities"] )

OR

(bobath*:ti,ab OR neurodevelopment*:ti,ab OR neuro-developmen:ti,ab OR neurophysiological:ti,ab OR neuro-physiological:ti,ab OR neuromuscular:ti,ab OR neuro-muscular:ti,ab OR NDT:ti,ab OR neurofacilitation* OR neuro-facilitation*:ti,ab OR neuro*facilitation:ti,ab)

OR

([mh "Virtual reality exposure therapy"] OR (virtual OR virtually OR VR):ti,ab OR [mh "user computer interface"] OR [mh "computer simulation"] OR "computer simulat*":ti,ab OR ((simulat* OR augment* OR mediat* ) NEAR/3 (world* OR realit* OR environment*

)):ti,ab OR [mh "video games"] OR videogame*:ti,ab OR ((video OR computer OR electronic OR online OR simulation OR "role playing") NEXT game*):ti,ab OR Wii:ti,ab OR ((head OR helmet) NEXT mounted ):ti,ab OR (immerse* OR "spatial presence" OR lifelike):ti,ab OR interactive*:ti,ab OR augment*:ti,ab OR computer*:ti,ab OR "serious gaming":ti,ab OR softwar:ti,ab OR "computer interface*":ti,ab OR exergam*:ti,ab OR "reality system*":ti,ab OR ((Nintendo OR sony OR gestureTek OR NeuroVR OR Hocoma OR Motek

OR "virtual realities" OR "haptic Master" OR Microsoft):ti,ab) OR ((Xbox OR "essential reality" OR SensAble OR Novint OR Cyberglove):ti,ab) )

OR

("web based":ti,ab OR [mh teletherapy] OR [mh telerehabilitation])

OR

("constraint induced movement therapy":ti,ab OR (constrain* NEAR/10 (movement* OR therap*)):ti,ab OR CIMT:ti,ab OR mCIMT:ti,ab OR "CI therap*":ti,ab OR forced:ti,ab OR "massed practice":ti,ab)

OR

("bimanual therapy":ti,ab OR "bimanual training":ti,ab OR "Hand arm bimanual intensive training":ti,ab OR HABIT:ti,ab OR "HABIT ILE":ti,ab)

OR

("functional therap*":ti,ab OR "task oriented training":ti,ab OR "task oriented training program":ti,ab OR "task oriented":ti,ab OR "goal directed training":ti,ab OR "motor training":ti,ab OR "task training":ti,ab OR "task practice":ti,ab OR "task-specific training":ti,ab OR physiotherap*:ti,ab OR "physical therap*":ti,ab OR "occupational therap*":ti,ab OR "functional therapy":ti,ab OR task OR OT:ti,ab)

OR

(COOP:ti,ab OR "cognitive orientation to occupational performance":ti,ab OR "CO OP":ti,ab)

OR

("context focussed":ti,ab)

OR

("action observation":ti,ab OR "action observation training":ti,ab OR "action observation therapy":ti,ab OR "action observation treatment":ti,ab)

OR

("mirror therapy":ti,ab OR "mirror therapy intervention":ti,ab OR "mirror therapy program":ti,ab OR "mirror box therapy":ti,ab OR mirror*:ti,ab)

OR

("Home activ*":ti,ab OR "Home based":ti,ab OR Home:ti,ab)

)

AND

([mh "upper extremity"] OR "upper extremit*":ti,ab OR "upper limb*":ti,ab OR hand:ti,ab OR hands:ti,ab OR arm:ti,ab OR arms:ti,ab OR manual:ti,ab OR bimanual:ti,ab OR Elbow:ti,ab OR elbows:ti,ab OR Forearm:ti,ab OR forearms:ti,ab OR Finger:ti,ab OR Fingers:ti,ab OR Thumb:ti,ab OR thumbs:ti,ab OR "fine motor*":ti,ab)

**CINAHL**

((MH "Cerebral palsy") OR TI "cerebral pals*" OR AB "cerebral pals*" OR TI "little* disease" OR AB "little* disease" OR TI CP OR AB CP OR ((TI unilateral OR AB unilateral) N3 (TI spastic* OR AB spastic*)) OR ((TI hemiplegi* OR AB hemiplegi*) N3 (TI spastic* OR AB spastic*)) OR ((TI diplegi* OR AB diplegi*) N3 (TI spastic* OR AB spastic*)) OR ((TI triplegi* OR AB triplegi*) N3 (TI spastic* OR AB spastic*)) OR ((TI bilateral OR AB bilateral OR TI bi-lateral OR AB bi-lateral) N3 (TI spastic* OR AB spastic*)) OR ((TI quadripleg* OR AB quadripleg*) N3 (TI spastic* OR AB spastic*)) OR (dystoni* N3 "cerebral palsy"))

AND

(

(Therap* OR TI therapy OR AB therapy OR Intervention OR Exercis* OR Habilitation OR Training OR Service* OR Treat* OR modalit* OR (MH "Rehabilitation+"))

OR

((MH "Physical medicine") OR (MH "Physical therapy modalities"))

OR

(TI bobath* OR AB bobath* OR TI neurodevelopment* OR AB neurodevelopment* OR TI neuro-development OR AB neuro-development OR TI neurophysiological OR AB neurophysiological OR TI neuro-physiological OR AB neuro-physiological OR TI neuromuscular OR AB neuromuscular OR TI neuro-muscular OR AB neuro-muscular OR TI NDT OR AB NDT OR neurofacilitation* OR TI neuro-facilitation* OR AB neuro-facilitation*)

OR

((MH "Virtual reality exposure therapy") OR (TI virtual OR AB virtual OR TI virtually OR AB virtually OR TI VR OR AB VR) OR (MH "user computer interface+") OR (MH "computer simulation+") OR TI "computer simulat*" OR AB "computer simulat*" OR ((TI simulat* OR AB simulat* OR TI augment* OR AB augment* OR TI mediat* OR AB mediat*) N3 (TI world* OR AB world* OR TI realit* OR AB realit* OR TI environment* OR AB environment*)) OR (MH "video games+") OR TI videogame* OR AB videogame* OR ((TI video OR AB video OR TI computer OR AB computer OR TI electronic OR AB electronic OR TI online OR AB online OR TI on-line OR AB on-line OR TI simulation OR AB simulation OR TI "role playing" OR AB "role playing") NEXT (TI "game*" OR AB "game*")) OR TI Wii OR AB Wii OR ((TI head OR AB head OR TI helmet OR AB helmet) NEXT (TI "mounted" OR AB "mounted")) OR (TI immerse* OR AB immerse* OR TI "spatial presence" OR AB "spatial presence" OR TI lifelike OR AB lifelike OR TI life-like OR AB life-like) OR TI interactive* OR AB interactive* OR TI augment* OR AB augment* OR TI computer* OR AB computer* OR TI "serious gaming" OR AB "serious gaming" OR TI software OR AB software OR TI "user-computer interface*" OR AB "user-computer interface*" OR TI exergam* OR AB exergam* OR TI "reality system*" OR AB "reality system*" OR (TI Nintendo OR AB Nintendo OR TI sony OR AB sony OR TI gestureTek OR AB gestureTek OR TI NeuroVR OR AB NeuroVR OR TI Hocoma OR AB Hocoma OR TI Motek OR AB Motek OR TI "virtual realities" OR AB "virtual realities" OR TI "haptic Master" OR AB "haptic Master" OR TI Microsoft OR AB Microsoft OR TI Xbox OR AB Xbox OR TI "essential reality" OR AB "essential reality" OR TI SensAble OR AB SensAble OR TI Novint OR AB Novint OR TI Cyberglove OR AB Cyberglove) )

OR

(TI web-based OR AB web-based OR (MH "telehealth+"))

OR

(TI "constraint induced movement therapy" OR AB "constraint induced movement therapy" OR ((TI constrain* OR AB constrain*) N10 (TI movement* OR AB movement* OR TI therap* OR AB therap*)) OR TI CIMT OR AB CIMT OR TI mCIMT OR AB mCIMT OR TI "CI therap*" OR AB "CI therap*" OR TI forced OR AB forced OR TI "massed practice" OR AB "massed practice")

OR

(TI "bimanual therapy" OR AB "bimanual therapy" OR TI "bimanual training" OR AB "bimanual training" OR TI "Hand arm bimanual intensive training" OR AB "Hand arm bimanual intensive training" OR TI HABIT OR AB HABIT OR TI HABIT-ILE OR AB HABIT-ILE)

OR

(TI "functional therap*" OR AB "functional therap*" OR TI "task oriented training" OR AB "task oriented training" OR TI "task oriented training program" OR AB "task oriented training program" OR TI "task oriented" OR AB "task oriented" OR TI "goal directed training" OR AB "goal directed training" OR TI "motor training" OR AB "motor training" OR TI "task training" OR AB "task training" OR TI "task practice" OR AB "task practice" OR TI "task-specific training" OR AB "task-specific training" OR TI physiotherap* OR AB physiotherap* OR TI "physical therap*" OR AB "physical therap*" OR TI "occupational therap*" OR AB "occupational therap*" OR TI "functional therapy" OR AB "functional therapy" OR task OR OT )

OR

(TI COOP OR AB COOP OR TI "cognitive orientation to occupational performance" OR AB "cognitive orientation to occupational performance" OR TI CO-OP OR AB CO-OP)

OR

(TI "context focussed" OR AB "context focussed")

OR

(TI "action observation" OR AB "action observation" OR TI "action observation training" OR AB "action observation training" OR TI "action observation therapy" OR AB "action observation therapy" OR TI "action observation treatment" OR AB "action observation treatment")

OR

(TI "mirror therapy" OR AB "mirror therapy" OR TI "mirror therapy intervention" OR AB "mirror therapy intervention" OR TI "mirror therapy program" OR AB "mirror therapy program" OR TI "mirror box therapy" OR AB "mirror box therapy" OR TI mirror* OR AB mirror*)

OR

(TI "Home active*" OR AB "Home active*" OR TI Home-based OR AB Home-based OR TI Home OR AB Home)

)

AND

((MH "upper extremity+") OR TI "upper extremit*" OR AB "upper extremit*" OR TI "upper limb*" OR AB "upper limb*" OR TI hand OR AB hand OR TI hands OR AB hands OR TI arm OR AB arm OR TI arms OR AB arms OR TI manual OR AB manual OR TI bimanual OR AB bimanual OR TI Elbow OR AB Elbow OR TI elbows OR AB elbows OR TI Forearm OR AB Forearm OR TI forearms OR AB forearms OR TI Finger OR AB Finger OR TI Fingers OR AB Fingers OR TI Thumb OR AB Thumb OR TI thumbs OR AB thumbs OR TI "fine motor*" OR AB "fine motor*")

AND

((MH "Quasi-Experimental Studies+") OR MH randomized controlled trials OR MH double‐blind studies OR MH single‐blind studies OR MH random assignment OR MH pretest‐posttest design OR MH cluster sample OR TI (randomised OR randomized) OR AB (random*) OR TI (trial) OR MH (sample size) AND AB (assigned OR allocated OR control) OR MH (placebos) OR PT (randomized controlled trial) OR AB (control W5 group) OR MH (crossover design) OR MH (comparative studies) OR AB (cluster W3 RCT)) NOT ((MH animals+ OR MH (animal studies) OR TI (animal model*)) NOT MH (human))

**WEB of SCIENCE (core collection)** (("Cerebral palsy" OR "cerebral pals*" OR "little* disease" OR CP OR (unilateral NEAR/3 spastic*) OR (hemiplegi* NEAR/3 spastic*) OR (diplegi* NEAR/3 spastic*) OR (triplegi* NEAR/3 spastic*) OR ((bilateral OR bi-lateral) NEAR/3 spastic*) OR (quadripleg* NEAR/3 spastic*)) OR (dystoni* NEAR/3 "cerebral palsy"))
AND
(

(Therap* OR therapy OR Intervention OR Exercis* OR Habilitation OR Training OR Service* OR Treat* OR modalit* OR Rehabilitation OR Therapeutics OR "Physical and Rehabilitation Medicine")
OR
(bobath* OR neurodevelopment* OR neuro-developmen OR neurophysiological OR neuro-physiological OR neuromuscular OR neuro-muscular OR NDT OR neurofacilitation* OR neuro-facilitation* OR neuro*facilitation)
OR
("Virtual reality exposure therapy" OR (virtual OR virtually OR VR) OR "user computer interface" OR "computer simulation" OR "computer simulat*" OR ((simulat* OR augment* OR mediat*) NEAR/3 (world* OR realit* OR environment*)) OR "video games" OR videogame* OR ((video OR computer OR electronic OR online OR on-line OR simulation OR "role playing") NEAR/1 game*) OR Wii OR ((head OR helmet) NEAR/1 mounted) OR (immerse* OR "spatial presence" OR lifelike OR life-like) OR interactive* OR augment* OR computer* OR "serious gaming" OR software OR "user-computer interface*" OR exergam* OR "reality system*" OR (Nintendo OR sony OR gestureTek OR NeuroVR OR Hocoma OR Motek OR "virtual realities" OR "haptic Master" OR Microsoft OR Xbox OR "essential reality" OR SensAble OR Novint OR Cyberglove) )
OR
(web-based OR telerehabilitation OR teletherapy OR teletherapy OR telerehabilitation)
OR
("constraint induced movement therapy" OR (constrain* NEAR/10 (movement* OR therap*)) OR CIMT OR mCIMT OR "CI therap*" OR forced OR "massed practice")
OR
("bimanual therapy" OR "bimanual training" OR "Hand arm bimanual intensive training" OR HABIT OR HABIT-ILE)
OR
("functional therap*" OR "task oriented training" OR "task oriented training program" OR "task oriented" OR "goal directed training" OR "motor training" OR "task training" OR "task practice" OR "task-specific training" OR physiotherap* OR "physical therap*" OR "occupational therap*" OR "functional therapy" OR task OR OT)
OR
(COOP OR "cognitive orientation to occupational performance" OR CO-OP)
OR
("context focussed")
OR
("action observation" OR "action observation training" OR "action observation therapy" OR "action observation treatment")
OR
("mirror therapy" OR "mirror therapy intervention" OR "mirror therapy program" OR "mirror box therapy" OR mirror*)
OR
("Home active*" OR Home-based OR Home)

)
AND
("upper extremity" OR "upper extremit*" OR "upper limb*" OR hand OR hands OR arm OR arms OR manual OR bimanual OR Elbow OR elbows OR Forearm OR forearms OR Finger OR Fingers OR Thumb OR thumbs OR "fine motor*")

AND
**RUN NEXT part separately**

Topic= (random* or placebo* or allocat* or crossover* or "cross over" or ((singl* or doubl*) NEAR/1 blind*))

Title=(trial)

*Do two lines above separately and then combine with OR*

**Then combine these with rest of search using AND, in the search history.**

# References

1. Sgandurra G, Ferrari, Adriano, , Cossu G, Guzzetta, Andrea, , Biagi L, Tosetti M, Fogassi L, Cioni G. Upper Limb Children Action-observation Training (UP-CAT): A Randomised Controlled Trial in Hemiplegic Cerebral Palsy. *BMC Neurology.* 2011;11(1).

2. Gordon AM, Schneider JA, Chinnan A, Charles JR. Efficacy of a hand-arm bimanual intensive therapy (HABIT) in children with hemiplegic cerebral palsy: a randomized control trial. *Developmental Medicine & Child Neurology.* 2007;49(11):830-838.

3. Figueiredo PRP, Mancini MC, Feitosa AM, et al. Hand-arm bimanual intensive therapy and daily functioning of children with bilateral cerebral palsy: a randomized controlled trial. *Developmental Medicine and Child Neurology.* 2020.

4. Araneda R, Herman E, Delcour L, et al. Mirror movements after bimanual intensive therapy in children with unilateral cerebral palsy: A randomized controlled trial. *Dev Med Child Neurol.* 2022.

5. Bleyenheuft Y, Ebner-Karestinos D, Surana B, et al. Intensive upper- and lower-extremity training for children with bilateral cerebral palsy: a quasi-randomized trial. *Developmental Medicine & Child Neurology.* 2017;59(6):625-633.

6. Taub E, Miller NE, Novack TA, et al. Technique to improve chronic motor deficit after stroke. . *Arch Phys Med Rehabil.* 1993;74:347-354.

7. Eliasson AC, Krumlinde-Sundholm L, Gordon AM, et al. Guidelines for future research in constraint-induced movement therapy for children with unilateral cerebral palsy: an expert consensus. *Dev Med Child Neurol.* 2014;56(2):125-137.

8. Hoare BJ, Wallen MA, Thorley MN, Jackman ML, Carey LM, Imms C. Constraint-induced movement therapy in children with unilateral cerebral palsy. *Cochrane Database Syst Rev.* 2019;4:CD004149.

9. Gimeno H, Polatajko H. The Cognitive Orientation to daily Occupational Performance approach in childhood-onset disabilities. *Dev Med Child Neurol.* 2025;67(8):977-985.

10. Polatajko HJ, Mandich A. *Enabling occupation in children : the cognitive orientation to daily occupational performance (CO-OP) approach.* Ontario, Ottowa: CAOT Publications ACE.; 2004.

11. Eliasson AC, Shaw K, Berg E, Krumlinde-Sundholm L. An ecological approach of Constraint Induced Movement Therapy for 2-3-year-old children: a randomized control trial. *Research in Developmental Disabilities.* 2011;32(6):2820-2828.

12. Novak I, Cusick A, Lannin N. Occupational therapy home programs for cerebral palsy: double-blind, randomized, controlled trial. *Pediatrics.* 2009;124(4):e606-614.

13. Verhaegh APM, Groen BE, Aarts PBM, et al. Multisensory Stimulation and Priming (MuSSAP) in 4-10 Months Old Infants with a Unilateral Brain Lesion: A Pilot Randomised Clinical Trial. *Occup Ther Int.* 2023;2023:8128407.

14. Gygax MJ, Schneider P, Newman CJ. Mirror therapy in children with hemiplegia: a pilot study. *Developmental Medicine and Child Neurology.* 2011;53(5):473-476.

15. Te Velde A, Morgan C, Finch-Edmondson M, et al. Neurodevelopmental Therapy for Cerebral Palsy: A Meta-analysis. *Pediatrics.* 2022;149(6).

16. Vaughan-Graham J, C. C. Defining a Bobath clinical framework - a modified e-Delphi study. *Physiother Theory Pract.* 2016;32:612-627.

17. Dong VA, Fong KN, Chen YF, Tseng SS, Wong LM. 'Remind-to-move' treatment versus constraint-induced movement therapy for children with hemiplegic cerebral palsy: a randomized controlled trial. *Developmental Medicine & Child Neurology.* 2017;59(2):160-167.

18. McLean B, Taylor S, Blair E, Valentine J, Carey L, Elliott C. Somatosensory Discrimination Intervention Improves Body Position Sense and Motor Performance in Children With Hemiplegic Cerebral Palsy. *American Journal of Occupational Therapy.* 2017;71(3):1-9.

19. Klingels K, Feys H, Molenaers G, et al. Randomized trial of modified constraint-induced movement therapy with and without an intensive therapy program in children with unilateral cerebral palsy. *Neurorehabilitation & Neural Repair.* 2013;27(9):799-807.

20. Simon-Martinez C, Mailleux L, Hoskens J, et al. Randomized controlled trial combining constraint-induced movement therapy and action-observation training in unilateral cerebral palsy: clinical effects and influencing factors of treatment response. *Therapeutic Advances in Neurological Disorders.* 2020;13.

21. Kuo HC, Gordon AM, Henrionnet A, Hautfenne S, Friel KM, Bleyenheuft Y. The effects of intensive bimanual training with and without tactile training on tactile function in children with unilateral spastic cerebral palsy: A pilot study. *Res Dev Disabil.* 2016;49-50:129-139.

22. Beani E, Menici V, Sicola E, et al. Effectiveness of the home-based training program Tele-UPCAT (Tele-monitored UPper Limb Children Action Observation Training) in unilateral cerebral palsy: a randomized controlled trial. *Eur J Phys Rehabil Med.* 2023.

23. Buccino G, Arisi D, Gough P, et al. Improving upper limb motor functions through action observation treatment: a pilot study in children with cerebral palsy. *Developmental Medicine & Child Neurology.* 2012;54(9):822-828.

24. Buccino G, Molinaro A, Ambrosi C, et al. Action Observation Treatment Improves Upper Limb Motor Functions in Children with Cerebral Palsy: A Combined Clinical and Brain Imaging Study. *Neural Plasticity.* 2018;2018:4843985.

25. Elbagoury WS, El-Saeed TM, Olama KA, Kamel MI. Functional-outcomes-of-verbaldirected-training-versus-visualdirected-training-in-children-with-unilateral-cerebral-palsy. *26.* 2022;1:1205-1210.

26. Kim DH, An DH, Yoo WG. Effects of live and video form action observation training on upper limb function in children with hemiparetic cerebral palsy. *Technology & Health Care.* 2018;26(3):437-443.

27. Kim DH. Comparison of short- and long-time action observation training (AOT) on upper limb function in children with cerebral palsy. *Physiotherapy Practice & Research.* 2020;41(1):53-58.

28. Kirkpatrick E, Pearse J, James P, Basu A. Effect of parent-delivered action observation therapy on upper limb function in unilateral cerebral palsy: a randomized controlled trial. *Developmental Medicine & Child Neurology.* 2016;58(10):1049-1056.

29. Palomo-Carrion R, Zuil-Escobar JC, Cabrera-Guerra M, Barreda-Martinez P, Martinez-Cepa CB. Mirror and action observation therapy in children with unilateral spastic cerebral palsy: a feasibility study. *Revista de Neurologia.* 2022;75(11):325-332.

30. Quadrelli E, Anzani A, Ferri M, et al. Electrophysiological correlates of action observation treatment in children with cerebral palsy: A pilot study. *Developmental Neurobiology.* 2019;79(11-12):934-948.

31. Sgandurra G, Ferrari A, Cossu G, Guzzetta A, Fogassi L, Cioni G. Randomized trial of observation and execution of upper extremity actions versus action alone in children with unilateral cerebral palsy. *Neurorehabilitation & Neural Repair.* 2013;27(9):808-815.

32. Araneda R, Ebner-Karestinos D, Paradis J, et al. Changes Induced by Early Hand-Arm Bimanual Intensive Therapy Including Lower Extremities in Young Children With Unilateral Cerebral Palsy: A Randomized Clinical Trial. *JAMA Pediatrics.* 2024;178(1):19-28.

33. Bleyenheuft Y, Arnould C, Brandao MB, Bleyenheuft C, Gordon AM. Hand and Arm Bimanual Intensive Therapy Including Lower Extremity (HABIT-ILE) in Children With Unilateral Spastic Cerebral Palsy: A Randomized Trial. *Neurorehabilitation & Neural Repair.* 2015;29(7):645-657.

34. Brandao MB, Ferre C, Kuo HC, et al. Comparison of Structured Skill and Unstructured Practice During Intensive Bimanual Training in Children With Unilateral Spastic Cerebral Palsy. *Neurorehabilitation & Neural Repair.* 2014;28(5):452-461.

35. Brandao MB, Mancini MC, Ferre CL, et al. Does Dosage Matter? A Pilot Study of Hand-Arm Bimanual Intensive Training (HABIT) Dose and Dosing Schedule in Children with Unilateral Cerebral Palsy. *Physical & Occupational Therapy in Pediatrics.* 2018;38(3):227-242.

36. Facchin P, Rosa-Rizzotto M, Pozza LVD, et al. Multisite Trial Comparing the Efficacy of Constraint-Induced Movement Therapy with that of Bimanual Intensive Training in Children with Hemiplegic Cerebral Palsy. *American Journal of Physical Medicine & Rehabilitation.* 2011;90(7):539-553.

37. Fedrizzi E, Rosa-Rizzotto M, Turconi AC, et al. Unimanual and bimanual intensive training in children with hemiplegic cerebral palsy and persistence in time of hand function improvement: 6-month follow-up results of a multisite clinical trial. *Journal of Child Neurology.* 2013;28(2):161-175.

38. Ferre CL, Brandao M, Surana B, Dew AP, Moreau NG, Gordon AM. Caregiver-directed home-based intensive bimanual training in young children with unilateral spastic cerebral palsy: a randomized trial. *Developmental Medicine & Child Neurology.* 2017;59(5):497-504.

39. Aarts PB, Jongerius PH, Geerdink YA, van Limbeek J, Geurts AC. Effectiveness of modified constraint-induced movement therapy in children with unilateral spastic cerebral palsy: a randomized controlled trial. *Neurorehabilitation & Neural Repair.* 2010;24(6):509-518.

40. Geerdink Y, Aarts P, Geurts AC. Motor learning curve and long-term effectiveness of modified constraint-induced movement therapy in children with unilateral cerebral palsy: a randomized controlled trial. *Research in Developmental Disabilities.* 2013;34(3):923-931.

41. Abd El-Kafy EM, Elshemy SA, Alghamdi MS. Effect of constraint-induced therapy on upper limb functions: a randomized control trial. *Scandinavian Journal of Occupational Therapy.* 2014;21(1):11-23.

42. Al-Oraibi S, Eliasson AC. Implementation of constraint-induced movement therapy for young children with unilateral cerebral palsy in Jordan: a home-based model. *Disability & Rehabilitation.* 2011;33(21-22):2006-2012.

43. de Brito Brandao M, Mancini MC, Vaz DV, Pereira de Melo AP, Fonseca ST. Adapted version of constraint-induced movement therapy promotes functioning in children with cerebral palsy: a randomized controlled trial. *Clinical Rehabilitation.* 2010;24(7):639-647.

44. Case-Smith J, DeLuca SC, Stevenson R, Ramey SL. Multicenter randomized controlled trial of pediatric constraint-induced movement therapy: 6-month follow-up. *American Journal of Occupational Therapy.* 2012;66(1):15-23.

45. DeLuca SC, Case-Smith J, Stevenson R, Ramey SL. Constraint-induced movement therapy (CIMT) for young children with cerebral palsy: effects of therapeutic dosage. *Journal of Pediatric Rehabilitation Medicine.* 2012;5(2):133-142.

46. Chamudot R, Parush S, Rigbi A, Horovitz R, Gross-Tsur V. Effectiveness of Modified Constraint-Induced Movement Therapy Compared With Bimanual Therapy Home Programs for Infants With Hemiplegia: A Randomized Controlled Trial. *American Journal of Occupational Therapy.* 2018;72(6):7206205010p7206205011-7206205010p7206205019.

47. Charles JR, Wolf SL, Schneider JA, Gordon AM. Efficacy of a child-friendly form of constraint-induced movement therapy in hemiplegic cerebral palsy: a randomized control trial. *Developmental Medicine & Child Neurology.* 2006;48(8):635-642.

48. Chen CL, Kang LJ, Hong WH, Chen FC, Chen HC, Wu CY. Effect of therapist-based constraint-induced therapy at home on motor control, motor performance and daily function in children with cerebral palsy: a randomized controlled study. *Clinical Rehabilitation.* 2013;27(3):236-245.

49. Chen HC, Chen CL, Kang LJ, Wu CY, Chen FC, Hong WH. Improvement of upper extremity motor control and function after home-based constraint induced therapy in children with unilateral cerebral palsy: immediate and long-term effects. *Archives of Physical Medicine & Rehabilitation.* 2014;95(8):1423-1432.

50. Hsin YJ, Chen FC, Lin KC, Kang LJ, Chen CL, Chen CY. Efficacy of Constraint-Induced Therapy on Functional Performance and Health-Related Quality of Life for Children With Cerebral Palsy: A Randomized Controlled Trial. *Journal of Child Neurology.* 2012;27(8):992-999.

51. Christmas PM, Sackley C, Feltham MG, Cummins C. A randomized controlled trial to compare two methods of constraint-induced movement therapy to improve functional ability in the affected upper limb in pre-school children with hemiplegic cerebral palsy: CATCH TRIAL. *Clinical Rehabilitation.* 2018;32(7):909-918.

52. Choudhary A, Gulati S, Kabra M, et al. Efficacy of modified constraint induced movement therapy in improving upper limb function in children with hemiplegic cerebral palsy: a randomized controlled trial. *Brain & Development.* 2013;35(9):870-876.

53. Deppe W, Thuemmler K, Fleischer J, Berger C, Meyer S, Wiedemann B. Modified constraint-induced movement therapy versus intensive bimanual training for children with hemiplegia - a randomized controlled trial. *Clinical Rehabilitation.* 2013;27(10):909-920.

54. Eliasson AC, Nordstrand L, Ek L, et al. The effectiveness of Baby-CIMT in infants younger than 12 months with clinical signs of unilateral-cerebral palsy; an explorative study with randomized design. *Research in Developmental Disabilities.* 2018;72:191-201.

55. Eugster-Buesch F, de Bruin ED, Boltshauser E, et al. Forced-use therapy for children with cerebral palsy in the community setting: a single-blinded randomized controlled pilot trial. *Journal of Pediatric Rehabilitation Medicine.* 2012;5(2):65-74.

56. Gelkop N, Burshtein DG, Lahav A, et al. Efficacy of constraint-induced movement therapy and bimanual training in children with hemiplegic cerebral palsy in an educational setting. *Physical & Occupational Therapy in Pediatrics.* 2015;35(1):24-39.

57. Gordon AM, Hung YC, Brandao M, et al. Bimanual training and constraint-induced movement therapy in children with hemiplegic cerebral palsy: a randomized trial. *Neurorehabilitation & Neural Repair.* 2011;25(8):692-702.

58. de Brito Brandao M, Gordon AM, Mancini MC. Functional impact of constraint therapy and bimanual training in children with cerebral palsy: a randomized controlled trial. *American Journal of Occupational Therapy.* 2012;66(6):672-681.

59. Hoare B, Imms C, Villanueva E, Rawicki HB, Matyas T, Carey L. Intensive therapy following upper limb botulinum toxin A injection in young children with unilateral cerebral palsy: a randomized trial. *Developmental Medicine & Child Neurology.* 2013;55(3):238-247.

60. Hwang YS, Kwon JY. Effects of Modified Constraint-Induced Movement Therapy in Real-World Arm Use in Young Children with Unilateral Cerebral Palsy: A Single-Blind Randomized Trial. *Neuropediatrics.* 2020;51(4):259-266.

61. Kirton A, Andersen J, Herrero M, et al. Brain stimulation and constraint for perinatal stroke hemiparesis: The PLASTIC CHAMPS Trial. *Neurology.* 2016;86(18):1659-1667.

62. Kuo HC, Zewdie E, Ciechanski P, Damji O, Kirton A. Intervention-Induced Motor Cortex Plasticity in Hemiparetic Children With Perinatal Stroke. *Neurorehabil Neural Repair.* 2018;32(11):941-952.

63. Liang KJ, Chen HL, Huang CW, Wang TN. Efficacy of Constraint-Induced Movement Therapy Versus Bimanual Intensive Training on Motor and Psychosocial Outcomes in Children With Unilateral Cerebral Palsy: A Randomized Trial. *Am J Occup Ther.* 2023;77(4).

64. Lin KC, Wang TN, Wu CY, et al. Effects of home-based constraint-induced therapy versus dose-matched control intervention on functional outcomes and caregiver well-being in children with cerebral palsy. *Research in Developmental Disabilities.* 2011;32(5):1483-1491.

65. Maitre NL, Jeanvoine A, Yoder PJ, et al. Kinematic and Somatosensory Gains in Infants with Cerebral Palsy After a Multi-Component Upper-Extremity Intervention: A Randomized Controlled Trial. *Brain Topography.* 2020.

66. Maring J, Wentzell E. Constraint Induced Movement Therapy: Impact of Setting on Outcomes. *Journal of Allied Health.* 2019;48(3):e73-e77.

67. Ostadzadeh A, Amini M, Hassani Mehraban A, Maroufizadeh S, Farajzadeh A. The Effect of Occupation-Based Modified Constraint-Induced Movement Therapy on the Participation of Children with Cerebral Palsy: A Single-Blind Randomized Controlled Trial. *Iran J Child Neurol.* 2023;17(2):39-54.

68. Palomo-Carrion R, Pinero-Pinto E, Ando-LaFuente S, Ferri-Morales A, Bravo-Esteban E, Romay-Barrero H. Unimanual Intensive Therapy with or without Unaffected Hand Containment in Children with Hemiplegia. A Randomized Controlled Pilot Study. *Journal of Clinical Medicine.* 2020;9(9):14.

69. Palomo-Carrion R, Lirio-Romero C, Ferri-Morales A, Jovellar-Isiegas P, Cortes-Vega MD, Romay-Barrero H. Combined intensive therapies at home in spastic unilateral cerebral palsy with high bimanual functional performance. What do they offer? A comparative randomised clinical trial. *Therapeutic Advances in Chronic Disease.* 2021;12:20406223211034996.

70. Ramey SL, DeLuca SC, Stevenson RD, Conaway M, Darragh AR, Lo W. Constraint-induced movement therapy for cerebral palsy: A randomized trial. *Pediatrics.* 2021;148(5).

71. Rostami HR, Malamiri RA. Effect of treatment environment on modified constraint-induced movement therapy results in children with spastic hemiplegic cerebral palsy: a randomized controlled trial. *Disability & Rehabilitation.* 2012;34(1):40-44.

72. Sakzewski L, Ziviani J, Abbott DF, Macdonell RAL, Jackson GD, Boyd RN. Randomized trial of constraint-induced movement therapy and bimanual training on activity outcomes for children with congenital hemiplegia. *Developmental Medicine and Child Neurology.* 2011;53(4):313-320.

73. Sakzewski L, Ziviani J, Abbott DF, Macdonell RA, Jackson GD, Boyd RN. Equivalent retention of gains at 1 year after training with constraint-induced or bimanual therapy in children with unilateral cerebral palsy. *Neurorehabilitation & Neural Repair.* 2011;25(7):664-671.

74. Sakzewski L, Ziviani J, Abbott DF, Macdonell RA, Jackson GD, Boyd RN. Participation Outcomes in a Randomized Trial of 2 Models of Upper-Limb Rehabilitation for Children With Congenital Hemiplegia. *Archives of Physical Medicine & Rehabilitation.* 2011;92(4):531-539.

75. Sakzewski L, Provan K, Ziviani J, Boyd RN. Comparison of dosage of intensive upper limb therapy for children with unilateral cerebral palsy: how big should the therapy pill be? *Research in Developmental Disabilities.* 2015;37:9-16.

76. Sakzewski L, Miller L, Ziviani J, et al. Randomized comparison trial of density and context of upper limb intensive group versus individualized occupational therapy for children with unilateral cerebral palsy. *Developmental Medicine & Child Neurology.* 2015;57(6):539-547.

77. Smania N, Aglioti SM, Cosentino A, et al. A modified constraint-induced movement therapy (CIT) program improves paretic arm use and function in children with cerebral palsy. *European journal of physical & rehabilitation medicine.* 2009;45(4):493-500.

78. Sung IY, Ryu JS, Pyun SB, Yoo SD, Song WH, Park MJ. Efficacy of forced-use therapy in hemiplegic cerebral palsy. *Archives of Physical Medicine & Rehabilitation.* 2005;86(11):2195-2198.

79. Taub E, Ramey SL, DeLuca S, Echols K. Efficacy of constraint-induced movement therapy for children with cerebral palsy with asymmetric motor impairment. *Pediatrics.* 2004;113(2):305-312.

80. Deluca SC, Echols K, Law CR, Ramey SL. Intensive pediatric constraint-induced therapy for children with cerebral palsy: randomized, controlled, crossover trial. *Journal of Child Neurology.* 2006;21(11):931-938.

81. Taub E, Griffin A, Uswatte G, Gammons K, Nick J, Law CR. Treatment of Congenital Hemiparesis With Pediatric Constraint-Induced Movement Therapy. *Journal of Child Neurology.* 2011;26(9):1163-1173.

82. Vaghela VG. To Study the effects of Mcimt Versus Cimt for Young Children with Spastic Hemiplegic Cerebral Palsy-- A Comparitive Study. *Indian Journal of Physiotherapy & Occupational Therapy.* 2014;8(2):136-141.

83. Wallen M, Ziviani J, Naylor O, Evans R, Novak I, Herbert RD. Modified constraint-induced therapy for children with hemiplegic cerebral palsy: a randomized trial. *Developmental Medicine & Child Neurology.* 2011;53(12):1091-1099.

84. Xu K, Wang L, Mai J, He L. Efficacy of constraint-induced movement therapy and electrical stimulation on hand function of children with hemiplegic cerebral palsy: a controlled clinical trial. *Disability & Rehabilitation.* 2012;34(4):337-346.

85. Yu J, Kang H, Jung J. Effects of modified constraint-induced movement therapy on hand dexterity, grip strength and activities of daily living of children with cerebral palsy: a randomized control trial. *Journal of physical therapy science.* 2012;24(10):1029‐1031.

86. Zafer H, Amjad I, Malik AN, Shaukat E. Effectiveness of constraint induced movement therapy as compared to bimanual therapy in upper motor function outcome in child with hemiplegic cerebral palsy. *Pakistan Journal of Medical Sciences.* 2016;32(1):181-184.

87. Bruchez R, Gygax MJ, Roches S, et al. Mirror therapy in children with hemiparesis: a randomized observer-blinded trial. *Developmental Medicine and Child Neurology.* 2016;58(9):970-978.

88. Elsepaee MI, Elhadidy EI, Emara HA, Nawar EAE. EFFECT OF MIRROR VISUAL FEEDBACK ON HAND FUNCTIONS IN CHILDREN WITH HEMIPARESIS. *International Journal of Physiotherapy.* 2016;3(2):147-153.

89. Kara OK, Yardimci BN, Sahin S, Orhan C, Livanelioglu A, Soylu AR. Combined Effects of Mirror Therapy and Exercises on the Upper Extremities in Children with Unilateral Cerebral Palsy: A Randomized Controlled Trial. *Developmental neurorehabilitation.* 2020;23(4):253-264.

90. Narimani A, Kalantari M, Dalvand H, Tabatabaee SM. Effect of mirror therapy on dexterity and hand grasp in children aged 9-14 years with hemiplegic cerebral palsy. *Iranian Journal of Child Neurology.* 2019;13(4):135-142.

91. Cameron D, Craig T, Edwards B, Missiuna C, Schwellnus H, Polatajko HJ. Cognitive Orientation to daily Occupational Performance (CO-OP): A New Approach for Children with Cerebral Palsy. *Phys Occup Ther Pediatr.* 2017;37(2):183-198.

92. Holmström L, Eliasson AC, Almeida R, et al. Efficacy of the small step program in a randomized controlled trial for infants under 12 months old at risk of cerebral palsy (CP) and other neurological disorders. *Journal of Clinical Medicine.* 2019;8(7).

93. Jackman M, Novak I, Lannin N, Froude E, Miller L, Galea C. Effectiveness of Cognitive Orientation to daily Occupational Performance over and above functional hand splints for children with cerebral palsy or brain injury: a randomized controlled trial. *BMC Pediatrics.* 2018;18(1):248.

94. Ko EJ, Sung IY, Moon HJ, Yuk JS, Kim H-S, Lee NH. Effect of Group-Task-Oriented Training on Gross and Fine Motor Function, and Activities of Daily Living in Children with Spastic Cerebral Palsy. *Physical & Occupational Therapy in Pediatrics.* 2020;40(1):18-30.

95. Law MC, Darrah J, Pollock N, et al. Focus on function: a cluster, randomized controlled trial comparing child- versus context-focused intervention for young children with cerebral palsy. *Developmental Medicine & Child Neurology.* 2011;53(7):621-629.

96. Moon J-H, Jung J-H, Hahm S-C, Cho H-y. The effects of task-oriented training on hand dexterity and strength in children with spastic hemiplegic cerebral palsy: A preliminary study. *Journal of physical therapy science.* 2017;29(10):1800-1802.

97. Sousa LK, Brandao MB, Curtin CM, Magalhaes LC. A Collaborative and Cognitive-based Intervention for Young People with Cerebral Palsy. *Canadian Journal of Occupational Therapy - Revue Canadienne d Ergotherapie.* 2020;87(4):319-330.

98. Yuan A, Hou M, Wang S, Liu Q, Li Y, Chen JI. Goals-activity-motor enrichment can improve the motor functioning of infants with a mild to moderate developmental disorder *Chinese Journal of Physical Medicine and Rehabilitation* 2023;12:808-812.

99. Wallen M, O'Flaherty SJ, Waugh MC. Functional outcomes of intramuscular botulinum toxin type a and occupational therapy in the upper limbs of children with cerebral palsy: a randomized controlled trial. *Archives of Physical Medicine & Rehabilitation.* 2007;88(1):1-10.

100. Friel KM, Ferre CL, Brandao M, et al. Improvements in Upper Extremity Function Following Intensive Training Are Independent of Corticospinal Tract Organization in Children With Unilateral Spastic Cerebral Palsy: A Clinical Randomized Trial. *Frontiers in neurology [electronic resource].* 2021;12:660780.

101. Afzal MT, Amjad I, Ghous M. Comparison of classic constraint-induced movement therapy and its modified form on upper extremity motor functions and psychosocial impact in hemiplegic cerebral palsy. *Journal of the Pakistan Medical Association.* 2022;72(7):1418-1421.

102. Bingol H, Gunel MK. Comparing the effects of modified constraint-induced movement therapy and bimanual training in children with hemiplegic cerebral palsy mainstreamed in regular school: A randomized controlled study. *Arch Pediatr.* 2022;29(2):105-115.

103. Dong AQ, Fong NK. Remind to move - A novel treatment on hemiplegic arm functions in children with unilateral cerebral palsy: A randomized cross-over study. *Developmental neurorehabilitation.* 2016;19(5):275-283.

104. Mohamed RA, Yousef AM, Radwan NL, Ibrahim MM. Efficacy of different approaches on quality of upper extremity function, dexterity and grip strength in hemiplegic children: a randomized controlled study. *European Review for Medical & Pharmacological Sciences.* 2021;25(17):5412-5423.

105. Rostami HR, Arastoo AA, Nejad SJ, Mahany MK, Malamiri RA, Goharpey S. Effects of modified constraint-induced movement therapy in virtual environment on upper-limb function in children with spastic hemiparetic cerebral palsy: a randomised controlled trial. *Neurorehabilitation.* 2012;31(4):357-365.
