# Supplementary material for: Active upper‐limb therapies for hand function, individual goal achievement, and self‐care in children with cerebral palsy: A network meta‐analysis
Source: Dev Med Child Neurol. 2025 Sep 5;67(12):1543–53. doi: 10.1111/dmcn.16476 (PMC12618955; doi:10.1111/dmcn.16476)
Supplement: Supplementary file 7 — Appendix S3: NMA and sensitivity analyses for COPM performance. [file DMCN-67-1543-s004.docx]

# Appendix S3 – NMA based on COPM-Performance

**Appendix S3.1 COPM – All studies involving COPM**

**Table S3.1. Studies used in COPM-Performance Network Meta Analyses, with group allocation, sample size, mean, SD, whether dosage was matched, risk of bias and age of children in study. Note that those rated as High risk of bias were excluded in final analysis presented in main text.**

| Study Author | Allocation (node) | n | Mean change | SD of change | Matched dosage | RoB | Age |
| --- | --- | --- | --- | --- | --- | --- | --- |
| Aarts2010_Geerdink2013* | Control | 22 | 1.20 | 1.10 | Yes | Some | Child (2-16y) |
| Aarts2010_Geerdink2013* | mCIMT | 28 | 3.50 | 1.30 | Yes | Some | Child (2-16y) |
| Araneda_2022* | BiM | 16 | 5.35 | 1.15 | No | Some | Child (2-16y) |
| Araneda_2022* | Control | 15 | 0.18 | 0.89 | No | Some | Child (2-16y) |
| Araneda_2024* | BiM | 25 | 4.18 | 1.67 | No | Some | All (0-16y) |
| Araneda_2024* | Control | 24 | 0.20 | 0.57 | No | Some | All (0-16y) |
| Bleyenheuft_2017* | BiM | 10 | 4.10 | 1.89 | No | High | Child (2-16y) |
| Bleyenheuft_2017* | Control | 6 | -0.10 | 0.53 | No | High | Child (2-16y) |
| Cameron_2017* | CO-OP | 9 | 4.50 | 2.50 | Yes | High | Child (2-16y) |
| Cameron_2017* | Control | 9 | 4.40 | 2.20 | Yes | High | Child (2-16y) |
| Ferre_2017 | BiM | 12 | 6.80 | 1.10 | Yes | Low | Child (2-16y) |
| Ferre_2017 | Control | 12 | 4.70 | 1.65 | Yes | Low | Child (2-16y) |
| Figueiredo_2020 | BiM | 19 | 7.20 | 1.10 | No | Low | Child (2-16y) |
| Figueiredo_2020 | Control | 20 | 3.70 | 1.60 | No | Low | Child (2-16y) |
| Friel_2021* | BiM | 39 | 3.40 | 1.70 | Yes | Low | Child (2-16y) |
| Friel_2021* | mCIMT | 39 | 2.90 | 1.80 | Yes | Low | Child (2-16y) |
| Gordon2011_Brandao2012* | BiM | 8 | 3.02 | 1.39 | Yes | High | Child (2-16y) |
| Gordon2011_Brandao2012* | mCIMT | 8 | 1.64 | 1.08 | Yes | High | Child (2-16y) |
| Hoare_2010* | BiM | 17 | 3.10 | 1.80 | Yes | Some | All (0-16y) |
| Hoare_2010* | mCIMT | 17 | 3.30 | 1.90 | Yes | Some | All (0-16y) |
| Kara_2020* | Control | 15 | 0.08 | 0.22 | No | Some | Child (2-16y) |
| Kara_2020* | Mirror | 15 | 2.69 | 1.25 | No | Some | Child (2-16y) |
| Kirton_2016* | Control | 12 | 2.10 | 1.50 | Yes | Low | Child (2-16y) |
| Kirton_2016* | mCIMT | 11 | 3.30 | 2.00 | Yes | Low | Child (2-16y) |
| McLean_2017* | Control | 10 | 0.39 | 1.40 | No | High | Child (2-16y) |
| McLean_2017* | Sensory+GDT | 7 | 3.99 | 1.17 | No | High | Child (2-16y) |
| Novak_2009* | Control | 12 | 0.00 | 0.00 | No | Some | Child (2-16y) |
| Novak_2009* | GDT | 11 | 1.60 | 2.56 | No | Some | Child (2-16y) |
| Ostadzadeh_2023* | mCIMT | 12 | 1.26 | 1.01 | Yes | Some | Child (2-16y) |
| Ostadzadeh_2023* | mCIMT_GDT | 10 | 2.94 | 1.21 | Yes | Some | Child (2-16y) |
| Sakzewski_2011_NNR_DMCN_APMR* | BiM | 31 | 2.80 | 1.60 | Yes | Some | Child (2-16y) |
| Sakzewski_2011_NNR_DMCN_APMR* | mCIMT | 32 | 2.90 | 2.00 | Yes | Some | Child (2-16y) |
| Sakzewski_2015_DMCN* | GDT | 17 | 3.40 | 2.10 | Yes | Some | Child (2-16y) |
| Sakzewski_2015_DMCN* | mCIMT | 24 | 2.70 | 1.40 | Yes | Some | Child (2-16y) |
| Sakzewski_2015_RIDD* | BiM | 9 | 3.10 | 2.30 | Yes | Low | Child (2-16y) |
| Sakzewski_2015_RIDD* | mCIMT | 9 | 1.80 | 1.70 | Yes | Low | Child (2-16y) |
| Sousa_2020* | CO-OP | 6 | 4.61 | 1.08 | Yes | Some | Child (2-16y) |
| Sousa_2020* | GDT | 6 | 1.00 | 1.26 | Yes | Some | Child (2-16y) |
| Wallen_2007* | Control | 15 | 1.20 | 1.20 | No | High | Child (2-16y) |
| Wallen_2007* | GDT | 17 | 2.10 | 1.70 | No | High | Child (2-16y) |
| Wallen_2011* | GDT | 25 | 3.10 | 1.70 | No | Low | All (0-16y) |
| Wallen_2011* | mCIMT | 25 | 3.60 | 2.10 | No | Low | All (0-16y) |

**Note: * beside author indicates that change data is used.**

Ferre 2017 and Figueiredo 2020 – post intervention data used, baseline scores of groups were very similar.

**Figure S3.1 Efficacy of interventions in COPM--units for a) All Studies, b) Studies classified as Low or Some risk of bias, c) studies with Matched Dosage and d) those with Matched Dosage and Low/Some risk of bias.**

**
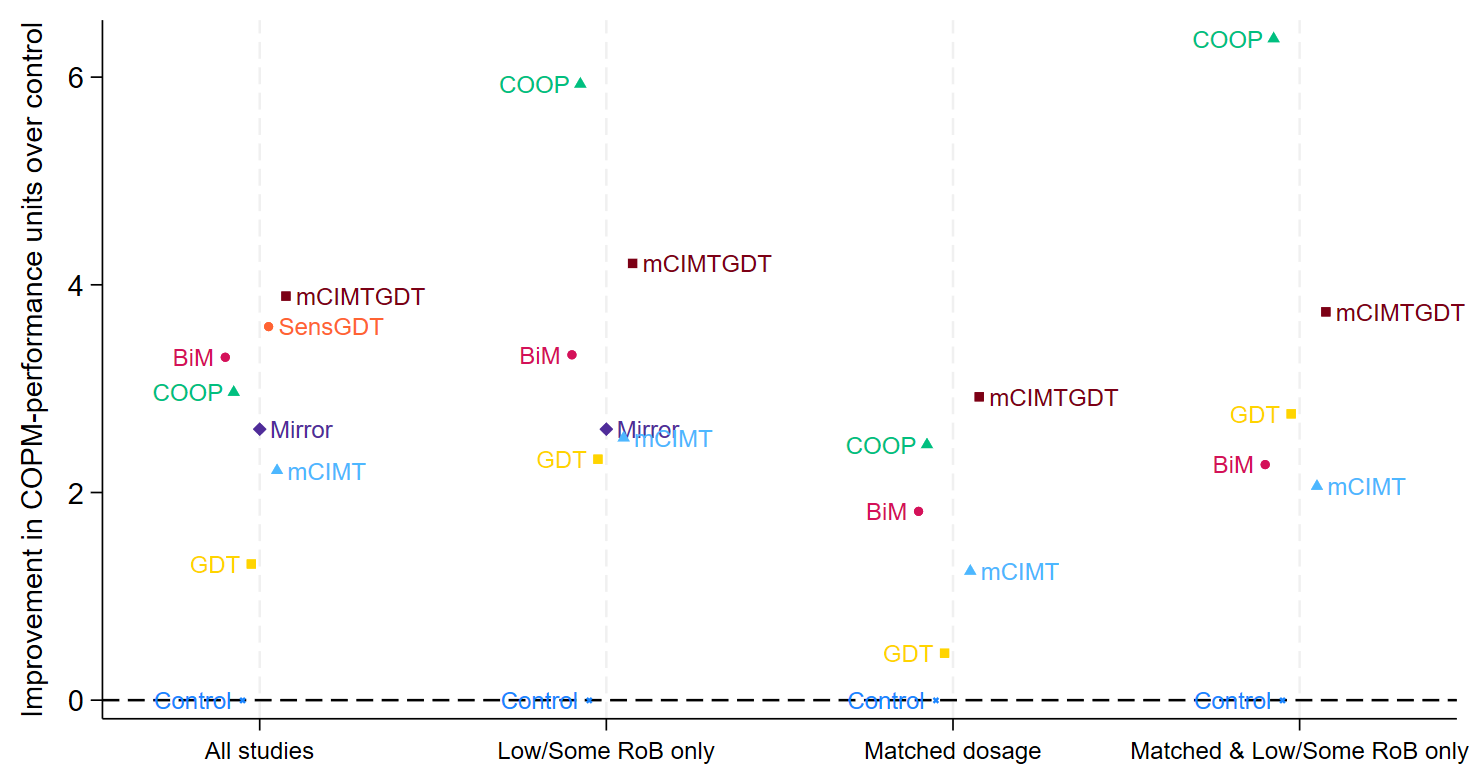
**

- NMA of Low/Some RoB studies only showed effect of GDT, mCIMT, Mirror, BiM, mCIMTint and CO-OP were all >2 COPM-Performance units compared to control.

##### S3.1 NMA of COPM-Performance – all studies

**Figure S3.1.1 Network map – COPM-Performance using all studies (21)**

**
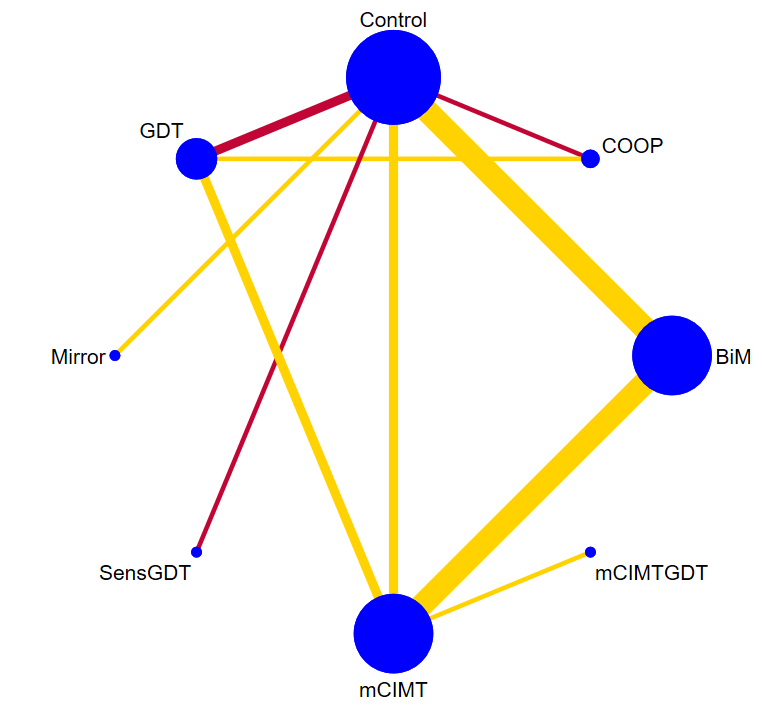
**

Node (circle) size indicates the number of studies reporting each intervention, width of lines is proportional to the number of trials comparing every pair of interventions, colour of lines indicates the most prevalent bias level [low (green), some concerns (yellow), high (red)] for each direct comparison (with the worst category chosen when there is a tie).

Key: AO-Action Observation, AOmCIMT-Action Observation+modified Contraint Induced Movement Therapy, BiM-Bimanual, COOP- Cognitive Orientation to daily Occupational Performance, GDT-Goal Directed Therapy, Mirror-Mirror Therapy, mCIMT-modified ConstraintNDT- Neurodevelopmental Treatment, mCIMT – modified Constraint Induced Movement Therapy; mCIMT- modified Constraint Induced Movement Therapy+Intensive, SensGDT-Sensory+GDT

**Table S3.1.1 of mean difference and its standard error for comparisons based on all COPM-Performance studies**


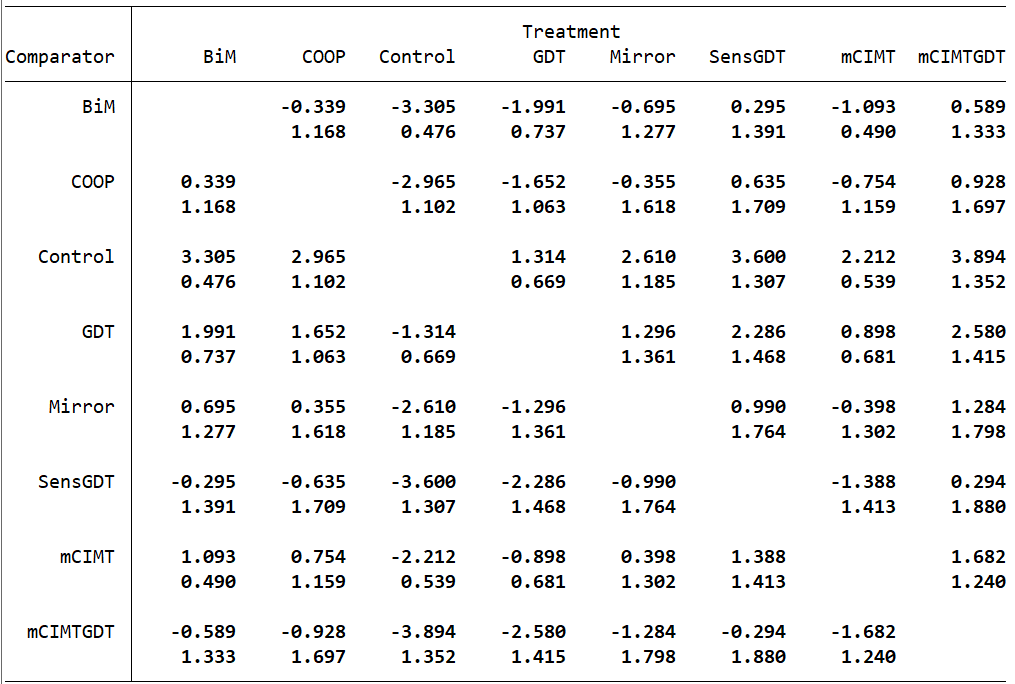


This table shows comparisons, for example, the effect of BiM is greater by 3.3 COPM-Performance units (SE=0.5) than Control. The effect of mCIMT is greater by 2.2 COPM-Performance units (SE=0.6) than Control.

**Figure S3.1.2. Network forest plot from NMA of all COPM-Performance papers**

**
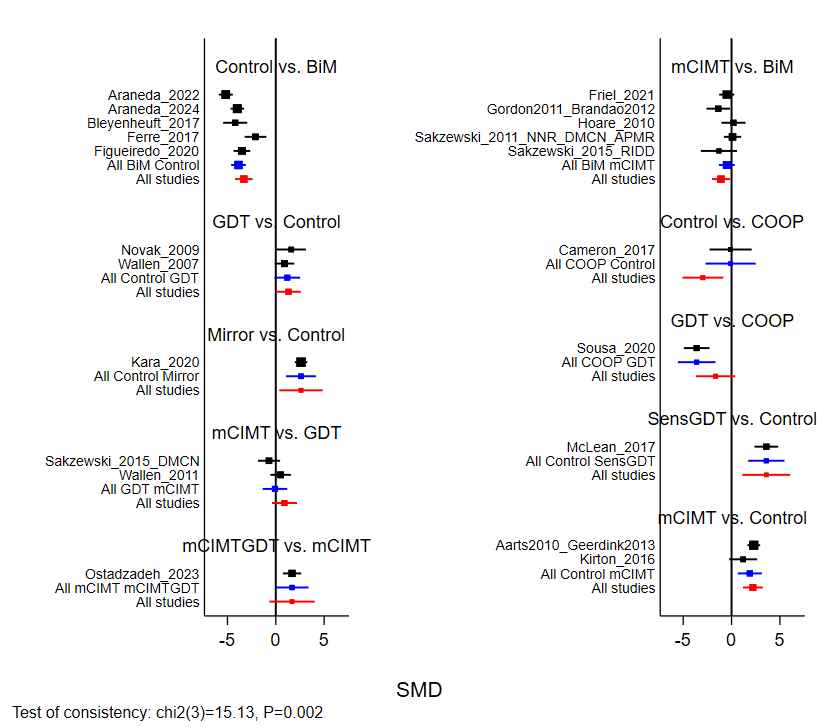
**

Note: Local inconsistency (network sidesplit all, tau) – p values of 0.055 for 5 comparisons and absent for two comparisons.

Key:

Black – effect of each study

Blue –pooled effect of direct comparisons within a trial design (e.g. two-arm RCT) as fit by an inconsistency model (“pooled within design”)

Red – Pooled overall effect (using direct and indirect information) as fit by a consistency model

Notes: (from Shim 2017)

“P-value is congruent with the result of the global test on inconsistency, which confirms that the consistency is accepted”

“Heterogeneity among individual studies within a treatment can be visually inspected. Moreover, based on the similarity between the size of pooled effect of each treatment in the comparison set (blue) and the size of pooled overall effect (red) it can be determined whether the consistency model is supported”

Notes: (from White 2015) The pooled estimates allow for between-studies heterogeneity, and thus may have wider confidence interval and smaller markers than study-specific estimates.

**Figure S3.4.3 Confidence intervals and prediction intervals for every treatment comparison (COPM-Performance all studies).**

**
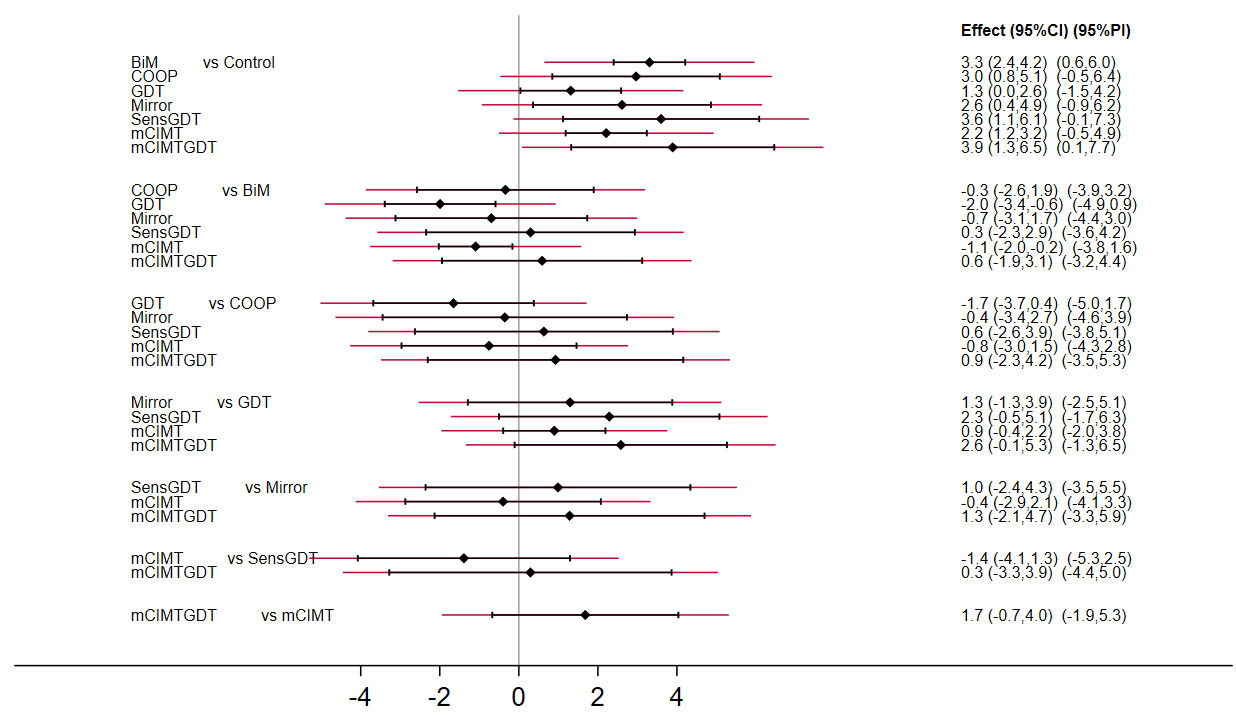
**

The Effect (95%CI) shows the average of the distribution of true effect sizes. The 95% PrI is the ‘prediction interval’, that is the next study that comes along will have a true effect size which is likely to be between these values.

##### S3.2 NMA of COPM-Performance – matched dosage

**Figure S3.2.1 Network map for COPM using studies with matched dosage (12 studies)**

**
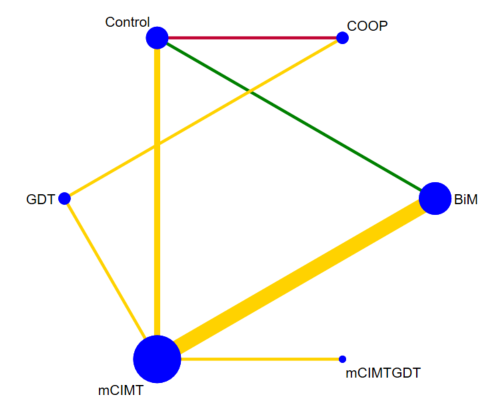
**

Node (circle) size indicates the number of studies reporting each intervention, width of lines is proportional to the number of trials comparing every pair of interventions, colour of lines indicates the most prevalent bias level [low (green), some concerns (yellow), high (red)] for each direct comparison (with the worst category chosen when there is a tie).

Key: AO-Action Observation, AOmCIMT-Action Observation+modified Contraint Induced Movement Therapy, BiM-Bimanual, COOP- Cognitive Orientation to daily Occupational Performance, GDT-Goal Directed Therapy, Mirror-Mirror Therapy, mCIMT-modified ConstraintNDT- Neurodevelopmental Treatment, mCIMT – modified Constraint Induced Movement Therapy; mCIMT- modified Constraint Induced Movement Therapy+Intensive, SensGDT-Sensory+GDT

**Table S3.2.1 Comparison of interventions showing Mean difference (and standard error) based on COPM-Performance data of studies with matched dosage.**

**
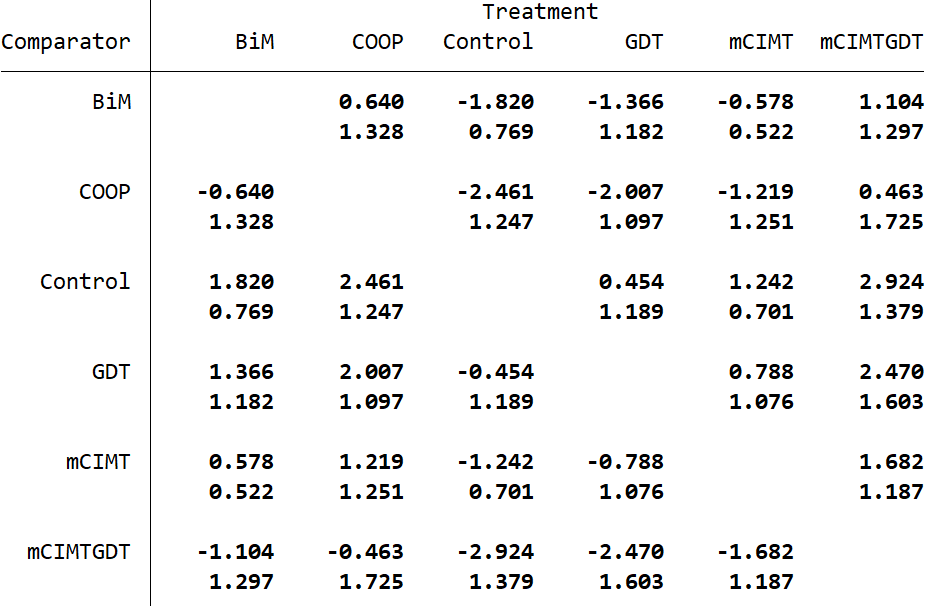
**

**Figure S3.2.2. Network forest plot based on COPM-Performance data of studies with matched dosage**

**
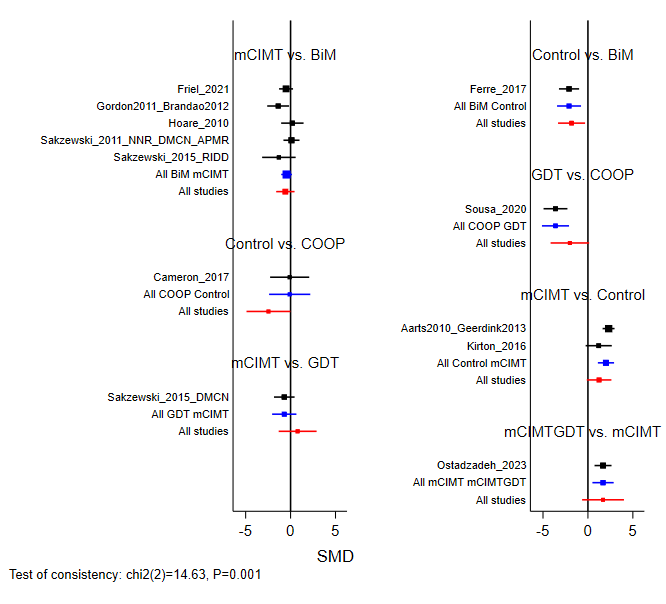
**

Note – p=0.001

Network sidesplit all, tau results: P=0.000 for COOP Control, COOP GDT, GDT mCIMT

Key:

Black – effect of each study

Blue –pooled effect of direct comparisons within a trial design (e.g. two-arm RCT) as fit by an inconsistency model (“pooled within design”)

Red – Pooled overall effect (using direct and indirect information) as fit by a consistency model

Notes: (from Shim 2017)

“P-value is congruent with the result of the global test on inconsistency, which confirms that the consistency is accepted”

“Heterogeneity among individual studies within a treatment can be visually inspected. Moreover, based on the similarity between the size of pooled effect of each treatment in the comparison set (blue) and the size of pooled overall effect (red) it can be determined whether the consistency model is supported”

Notes: (from White 2015) The pooled estimates allow for between-studies heterogeneity, and thus may have wider confidence interval and smaller markers than study-specific estimates.

**Figure S3.2.3 Confidence intervals and prediction intervals for every treatment based on COPM-Performance data of studies with matched dosage**

**
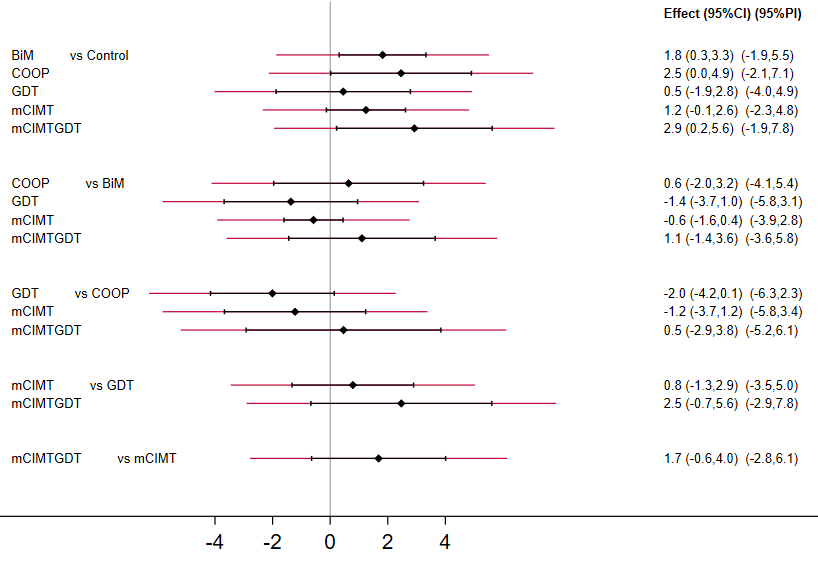
**

The Effect (95%CI) shows the average of the distribution of true effect sizes. The 95% PI is the ‘prediction interval’, that is the next study that comes along will have a true effect size which is likely to be between these values.

##### S3.3 NMA of COPM-Performance - papers with High RoB removed.

**Figure S3.3.1 Network map for COPM-Performance using studies with high RoB removed (15 studies)**


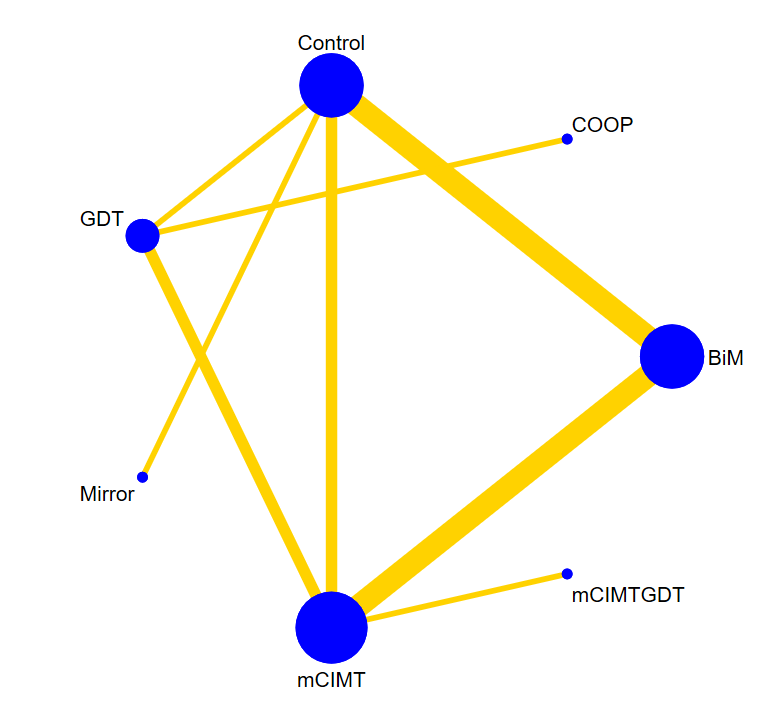


Node (circle) size indicates the number of studies reporting each intervention, width of lines is proportional to the number of trials comparing every pair of interventions, colour of lines indicates the most prevalent bias level [low (green), some concerns (yellow), high (red)] for each direct comparison (with the worst category chosen when there is a tie).

Key: AO-Action Observation, AOmCIMT-Action Observation+modified Contraint Induced Movement Therapy, BiM-Bimanual, COOP- Cognitive Orientation to daily Occupational Performance, GDT-Goal Directed Therapy, Mirror-Mirror Therapy, mCIMT-modified ConstraintNDT- Neurodevelopmental Treatment, mCIMT – modified Constraint Induced Movement Therapy; mCIMT- modified Constraint Induced Movement Therapy+Intensive, SensGDT-Sensory+GDT

**Table S3.3.1 Mean differences (and standard error) for comparisons,**

**based on COPM-Performance data (studies with high RoB removed).**


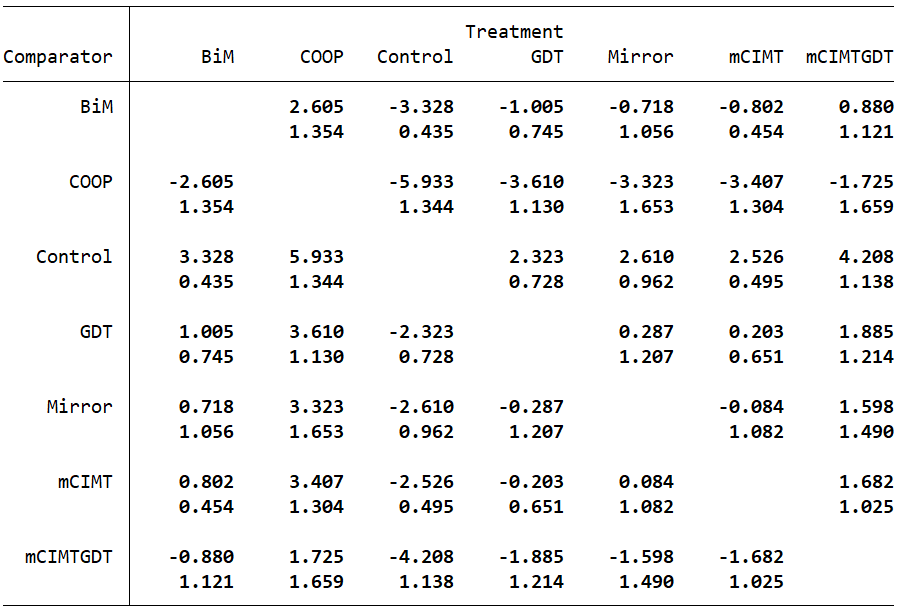


**Figure S3.3.2. Network forest plot based on COPM-Performance data where studies with high risk of bias removed.**

**
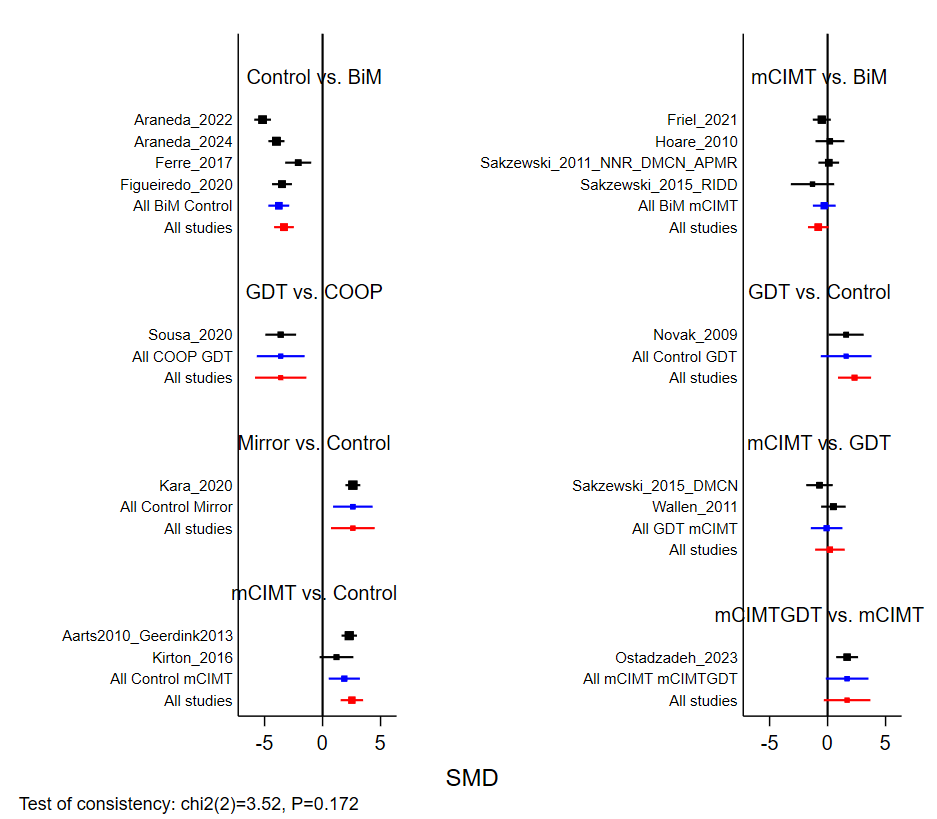
**

Key:

Black – effect of each study

Blue –pooled effect of direct comparisons within a trial design (e.g. two-arm RCT) as fit by an inconsistency model (“pooled within design”)

Red – Pooled overall effect (using direct and indirect information) as fit by a consistency model

Notes: (from Shim 2017)

“P-value is congruent with the result of the global test on inconsistency, which confirms that the consistency is accepted”

“Heterogeneity among individual studies within a treatment can be visually inspected. Moreover, based on the similarity between the size of pooled effect of each treatment in the comparison set (blue) and the size of pooled overall effect (red) it can be determined whether the consistency model is supported”

Notes: (from White 2015) The pooled estimates allow for between-studies heterogeneity, and thus may have wider confidence interval and smaller markers than study-specific estimates.

**Figure S3.3.3 Confidence intervals and prediction intervals (high risk of bias removed)**


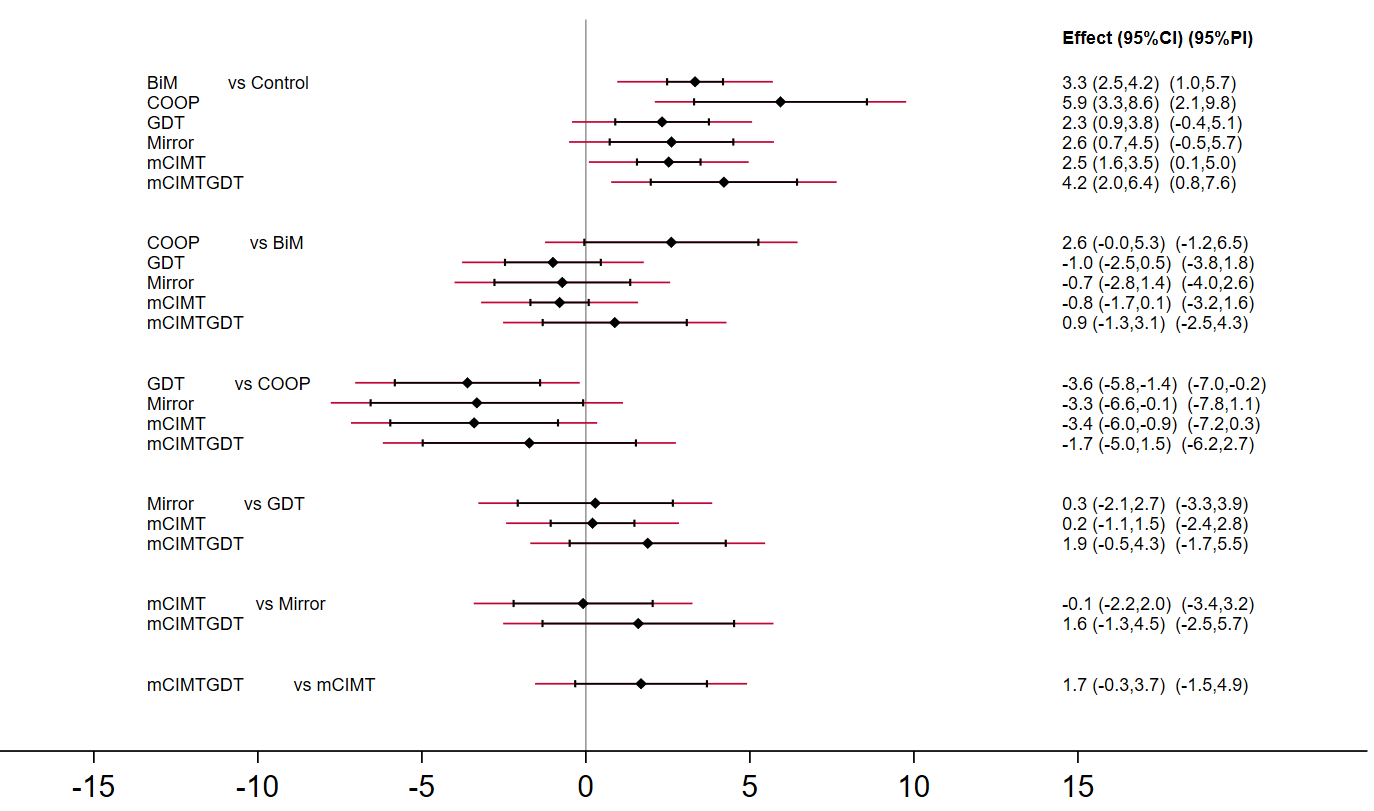


The Effect (95%CI) shows the average of the distribution of true effect sizes. The 95% PI is the ‘prediction interval’, that is the next study that comes along will have a true effect size which is likely to be between these values.

**Figure S3.3.4 Plots of the surface under the cumulative ranking curves (SUCRA) for all treatments in the COPM-Performance network where studies with high risk of bias were excluded.**


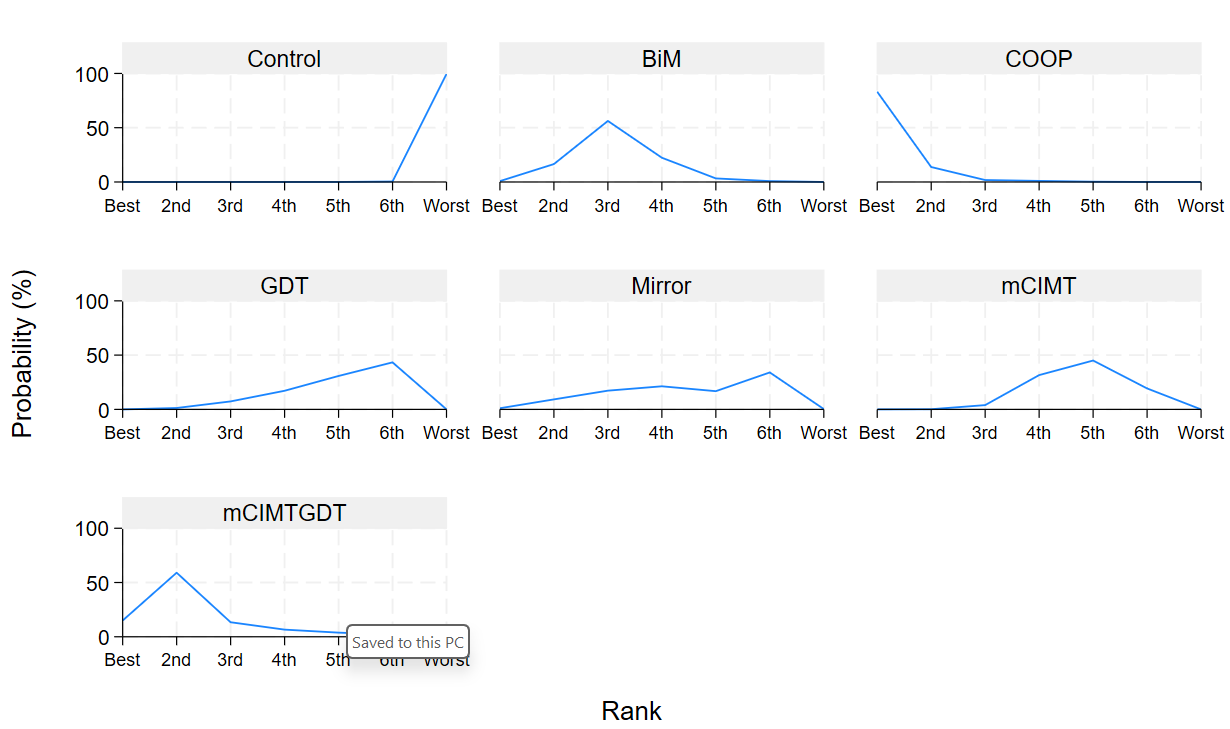


**Table S3.3.1 Estimated probabilities (%) of each treatment being the best (and other ranks) in network for COPM-Performance (studies at high risk of bias excluded)**

Estimated probabilities (%) of each treatment being the best (and other ranks)

---------------------------------------------------------------------------------------------

| Treatment

Author and Rank | Control BiM COOP GDT Mirror mCIMT mCIMTGDT

-----------------------+---------------------------------------------------------------------

Aarts2010_Geerdink2013 |

Best | 0 1 83 0 1 0 15

2nd | 0 17 14 1 9 0 59

3rd | 0 56 2 7 17 4 13

4th | 0 22 1 17 21 32 7

5th | 0 3 0 31 17 45 4

6th | 0 1 0 43 34 19 2

Worst | 100 0 0 0 0 0 0

MEAN RANK | 7 3 1 5 4 5 2

SUCRA | 0 1 1 0 0 0 1

---------------------------------------------------------------------------------------------

Key: AO-Action Observation, AOmCIMT-Action Observation+modified Contraint Induced Movement Therapy, BiM-Bimanual, COOP- Cognitive Orientation to daily Occupational Performance, GDT-Goal Directed Therapy, Mirror-Mirror Therapy, mCIMT-modified ConstraintNDT- Neurodevelopmental Treatment, mCIMT – modified Constraint Induced Movement Therapy; mCIMT- modified Constraint Induced Movement Therapy+Intensive, SensGDT-Sensory+GDT

##### S3.4 NMA of COPM-Performance - unmatched dosage excluded, & high RoB excluded

**Figure A2.4.1 Network map (10 studies)**


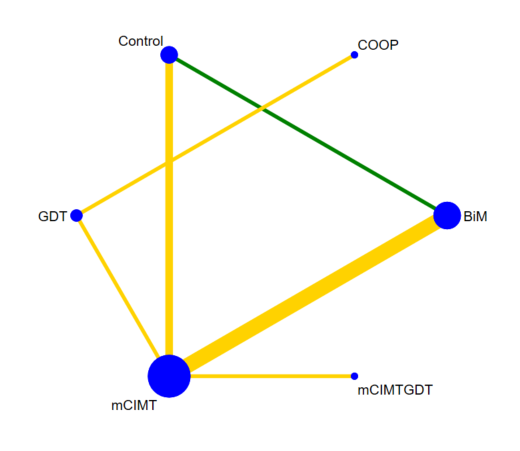


Node (circle) size indicates the number of studies reporting each intervention, width of lines is proportional to the number of trials comparing every pair of interventions, colour of lines indicates the most prevalent bias level [low (green), some concerns (yellow), high (red)] for each direct comparison (with the worst category chosen when there is a tie).

Key: AO-Action Observation, AOmCIMT-Action Observation+modified Contraint Induced Movement Therapy, BiM-Bimanual, COOP- Cognitive Orientation to daily Occupational Performance, GDT-Goal Directed Therapy, Mirror-Mirror Therapy, mCIMT-modified ConstraintNDT- Neurodevelopmental Treatment, mCIMT – modified Constraint Induced Movement Therapy; mCIMT- modified Constraint Induced Movement Therapy+Intensive, SensGDT-Sensory+GDT

**Figure S3.4.2 Scatter plot showing efficacy of interventions in COPM-Performance units in analysis with matched dosage included and high RoB removed**


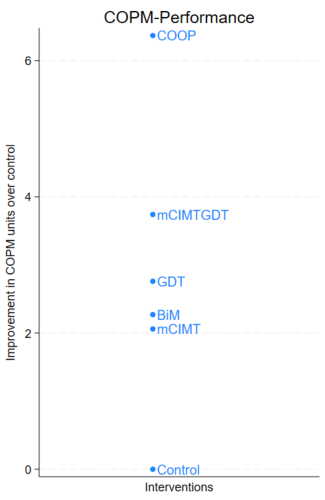


**Table S3.4.1 Mean differences (and standard error) for comparisons**


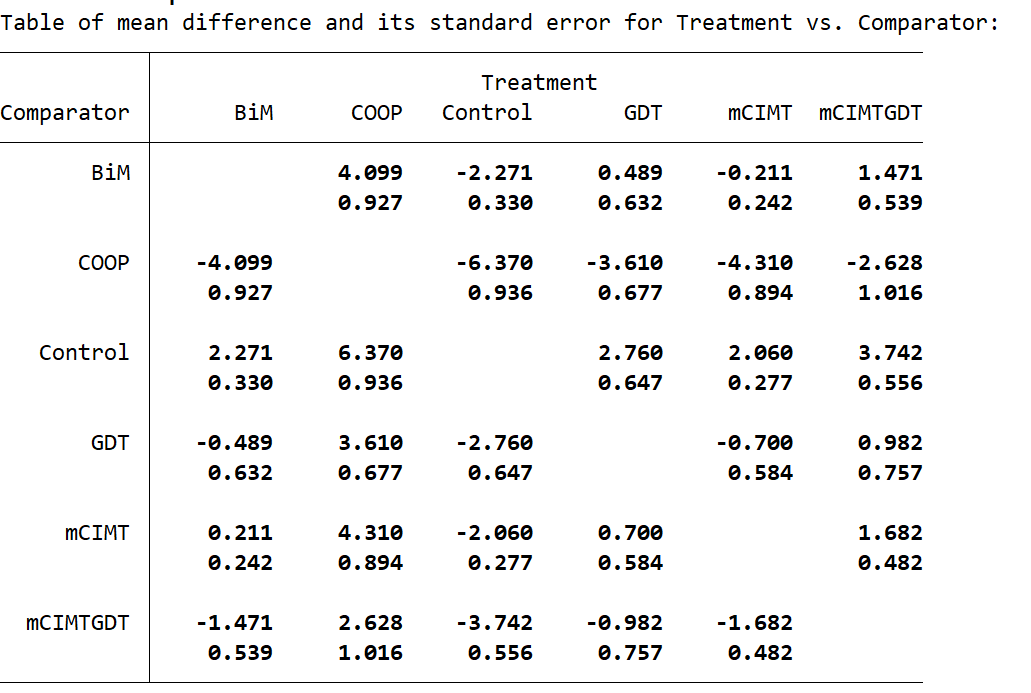


**Figure S3.4.3. Network forest plot based on COPM-Performance data of studies with unmatched dosage excluded and high RoB excluded**


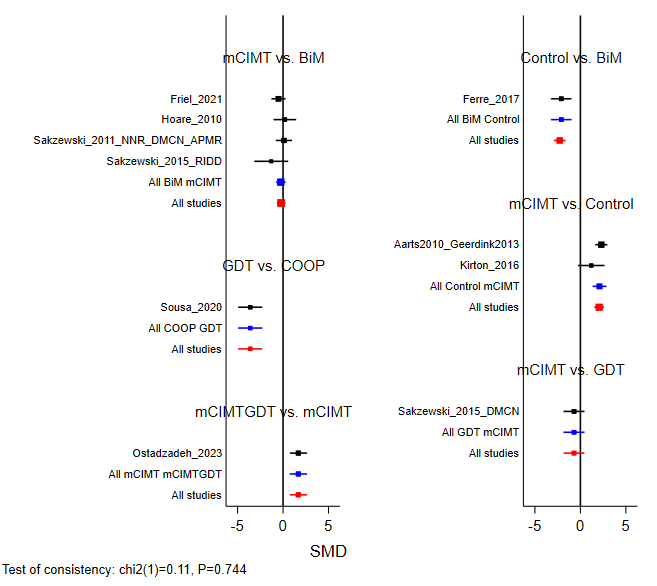


Key:

Black – effect of each study

Blue –pooled effect of direct comparisons within a trial design (e.g. two-arm RCT) as fit by an inconsistency model (“pooled within design”)

Red – Pooled overall effect (using direct and indirect information) as fit by a consistency model

Notes: (from Shim 2017)

“P-value is congruent with the result of the global test on inconsistency, which confirms that the consistency is accepted”

“Heterogeneity among individual studies within a treatment can be visually inspected. Moreover, based on the similarity between the size of pooled effect of each treatment in the comparison set (blue) and the size of pooled overall effect (red) it can be determined whether the consistency model is supported”

Notes: (from White 2015) The pooled estimates allow for between-studies heterogeneity, and thus may have wider confidence interval and smaller markers than study-specific estimates.

**Figure S2.4.4 Confidence intervals and prediction intervals for every treatment**


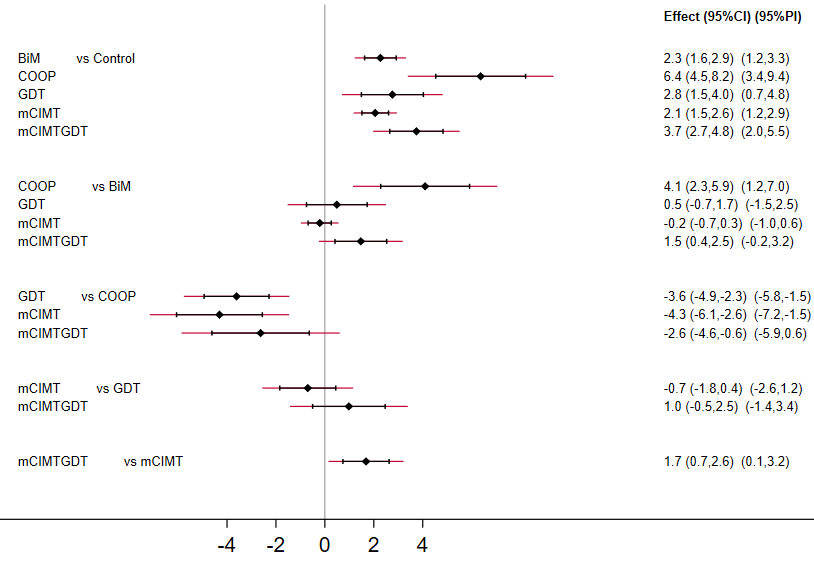


The Effect (95%CI) shows the average of the distribution of true effect sizes. The 95% PI is the ‘prediction interval’, that is the next study that comes along will have a true effect size which is likely to be between these values.

##### S3.5 NMA of COPM-Performance - matched dosage, Dosage 25 or more hours.

Figure S3.5.1 Network map of COPM-Performance data where dosage was matched and >25 hours (8 studies)


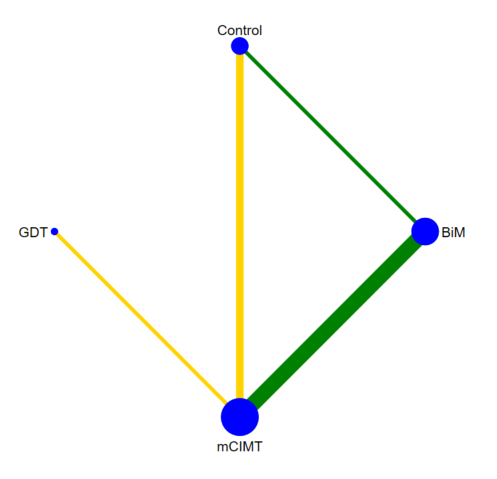


Node (circle) size indicates the number of studies reporting each intervention, width of lines is proportional to the number of trials comparing every pair of interventions, colour of lines indicates the most prevalent bias level [low (green), some concerns (yellow), high (red)] for each direct comparison (with the worst category chosen when there is a tie).

Key: BiM-Bimanual, GDT-Goal Directed Therapy, Mirror-Mirror Therapy, mCIMT- modified Constraint Induced Movement Therapy

**Figure S3.5.2 Scatter plot showing efficacy of interventions in COPM-Performance units (studies with dosage 25+ hours and matched).**

**
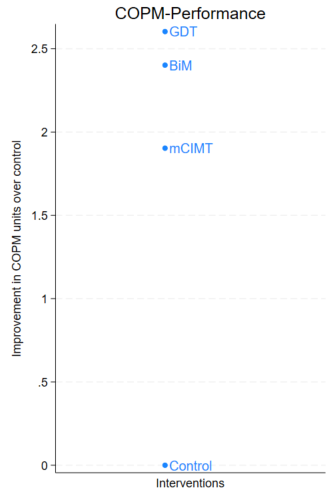
**

**Table S3.5.1 Mean differences (and standard error) for comparisons.**

**
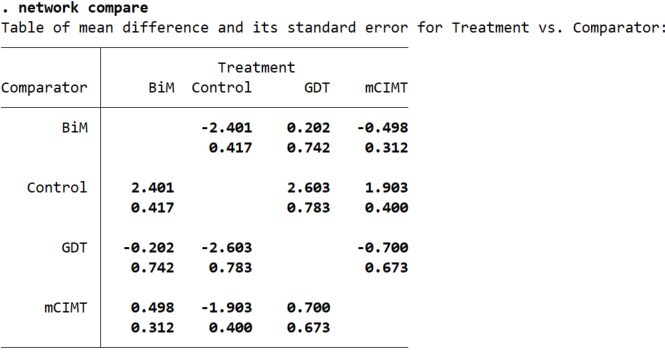
**

**Figure S3.5.3. Network forest plot based on COPM-Performance data of studies with matched dosage and 25+ hours dosage.**


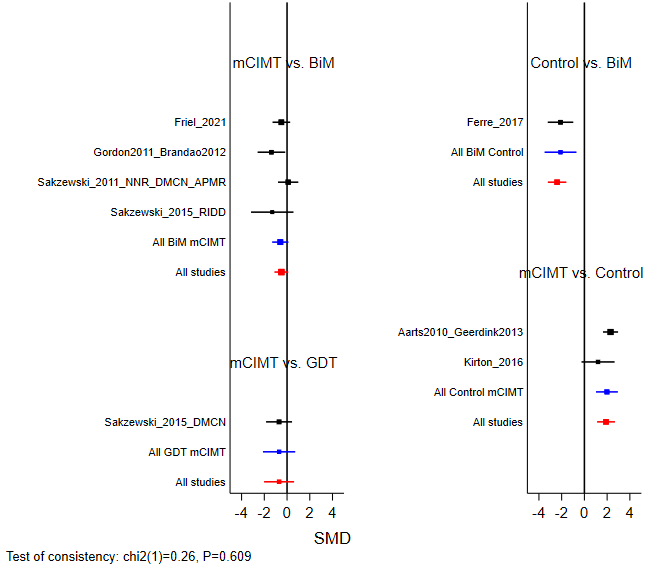


Key:

Black – effect of each study

Blue –pooled effect of direct comparisons within a trial design (e.g. two-arm RCT) as fit by an inconsistency model (“pooled within design”)

Red – Pooled overall effect (using direct and indirect information) as fit by a consistency model

Notes: (from Shim 2017)

“P-value is congruent with the result of the global test on inconsistency, which confirms that the consistency is accepted”

“Heterogeneity among individual studies within a treatment can be visually inspected. Moreover, based on the similarity between the size of pooled effect of each treatment in the comparison set (blue) and the size of pooled overall effect (red) it can be determined whether the consistency model is supported”

Notes: (from White 2015) The pooled estimates allow for between-studies heterogeneity, and thus may have wider confidence interval and smaller markers than study-specific estimates.

**Figure S3.5.4 Confidence intervals and prediction intervals for every treatment**


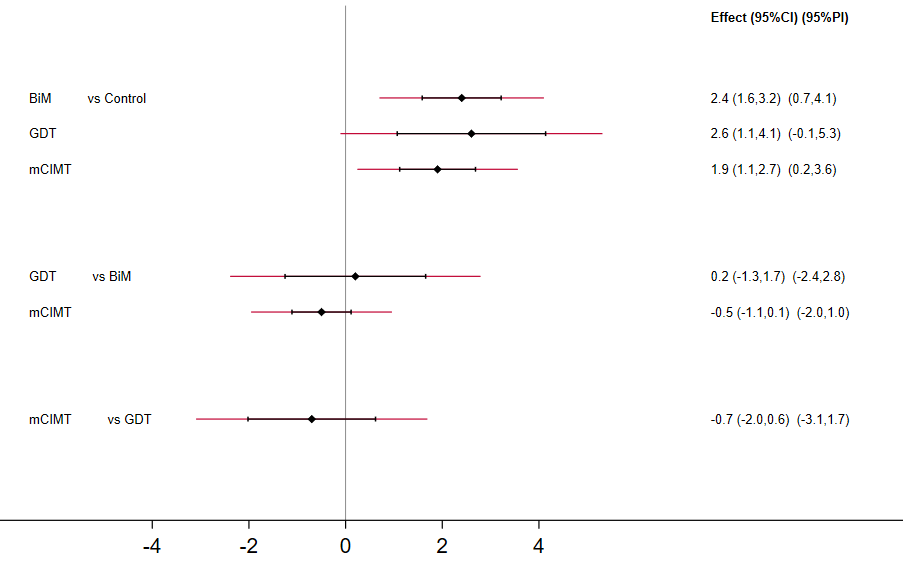


The Effect (95%CI) shows the average of the distribution of true effect sizes. The 95% PI is the ‘prediction interval’, that is the next study that comes along will have a true effect size which is likely to be between these values.

# References

1. Sgandurra G, Ferrari, Adriano, , Cossu G, Guzzetta, Andrea, , Biagi L, Tosetti M, Fogassi L, Cioni G. Upper Limb Children Action-observation Training (UP-CAT): A Randomised Controlled Trial in Hemiplegic Cerebral Palsy. *BMC Neurology.* 2011;11(1).

2. Gordon AM, Schneider JA, Chinnan A, Charles JR. Efficacy of a hand-arm bimanual intensive therapy (HABIT) in children with hemiplegic cerebral palsy: a randomized control trial. *Developmental Medicine & Child Neurology.* 2007;49(11):830-838.

3. Figueiredo PRP, Mancini MC, Feitosa AM, et al. Hand-arm bimanual intensive therapy and daily functioning of children with bilateral cerebral palsy: a randomized controlled trial. *Developmental Medicine and Child Neurology.* 2020.

4. Araneda R, Herman E, Delcour L, et al. Mirror movements after bimanual intensive therapy in children with unilateral cerebral palsy: A randomized controlled trial. *Dev Med Child Neurol.* 2022.

5. Bleyenheuft Y, Ebner-Karestinos D, Surana B, et al. Intensive upper- and lower-extremity training for children with bilateral cerebral palsy: a quasi-randomized trial. *Developmental Medicine & Child Neurology.* 2017;59(6):625-633.

6. Taub E, Miller NE, Novack TA, et al. Technique to improve chronic motor deficit after stroke. . *Arch Phys Med Rehabil.* 1993;74:347-354.

7. Eliasson AC, Krumlinde-Sundholm L, Gordon AM, et al. Guidelines for future research in constraint-induced movement therapy for children with unilateral cerebral palsy: an expert consensus. *Dev Med Child Neurol.* 2014;56(2):125-137.

8. Hoare BJ, Wallen MA, Thorley MN, Jackman ML, Carey LM, Imms C. Constraint-induced movement therapy in children with unilateral cerebral palsy. *Cochrane Database Syst Rev.* 2019;4:CD004149.

9. Gimeno H, Polatajko H. The Cognitive Orientation to daily Occupational Performance approach in childhood-onset disabilities. *Dev Med Child Neurol.* 2025;67(8):977-985.

10. Polatajko HJ, Mandich A. *Enabling occupation in children : the cognitive orientation to daily occupational performance (CO-OP) approach.* Ontario, Ottowa: CAOT Publications ACE.; 2004.

11. Eliasson AC, Shaw K, Berg E, Krumlinde-Sundholm L. An ecological approach of Constraint Induced Movement Therapy for 2-3-year-old children: a randomized control trial. *Research in Developmental Disabilities.* 2011;32(6):2820-2828.

12. Novak I, Cusick A, Lannin N. Occupational therapy home programs for cerebral palsy: double-blind, randomized, controlled trial. *Pediatrics.* 2009;124(4):e606-614.

13. Verhaegh APM, Groen BE, Aarts PBM, et al. Multisensory Stimulation and Priming (MuSSAP) in 4-10 Months Old Infants with a Unilateral Brain Lesion: A Pilot Randomised Clinical Trial. *Occup Ther Int.* 2023;2023:8128407.

14. Gygax MJ, Schneider P, Newman CJ. Mirror therapy in children with hemiplegia: a pilot study. *Developmental Medicine and Child Neurology.* 2011;53(5):473-476.

15. Te Velde A, Morgan C, Finch-Edmondson M, et al. Neurodevelopmental Therapy for Cerebral Palsy: A Meta-analysis. *Pediatrics.* 2022;149(6).

16. Vaughan-Graham J, C. C. Defining a Bobath clinical framework - a modified e-Delphi study. *Physiother Theory Pract.* 2016;32:612-627.

17. Dong VA, Fong KN, Chen YF, Tseng SS, Wong LM. 'Remind-to-move' treatment versus constraint-induced movement therapy for children with hemiplegic cerebral palsy: a randomized controlled trial. *Developmental Medicine & Child Neurology.* 2017;59(2):160-167.

18. McLean B, Taylor S, Blair E, Valentine J, Carey L, Elliott C. Somatosensory Discrimination Intervention Improves Body Position Sense and Motor Performance in Children With Hemiplegic Cerebral Palsy. *American Journal of Occupational Therapy.* 2017;71(3):1-9.

19. Klingels K, Feys H, Molenaers G, et al. Randomized trial of modified constraint-induced movement therapy with and without an intensive therapy program in children with unilateral cerebral palsy. *Neurorehabilitation & Neural Repair.* 2013;27(9):799-807.

20. Simon-Martinez C, Mailleux L, Hoskens J, et al. Randomized controlled trial combining constraint-induced movement therapy and action-observation training in unilateral cerebral palsy: clinical effects and influencing factors of treatment response. *Therapeutic Advances in Neurological Disorders.* 2020;13.

21. Kuo HC, Gordon AM, Henrionnet A, Hautfenne S, Friel KM, Bleyenheuft Y. The effects of intensive bimanual training with and without tactile training on tactile function in children with unilateral spastic cerebral palsy: A pilot study. *Res Dev Disabil.* 2016;49-50:129-139.

22. Beani E, Menici V, Sicola E, et al. Effectiveness of the home-based training program Tele-UPCAT (Tele-monitored UPper Limb Children Action Observation Training) in unilateral cerebral palsy: a randomized controlled trial. *Eur J Phys Rehabil Med.* 2023.

23. Buccino G, Arisi D, Gough P, et al. Improving upper limb motor functions through action observation treatment: a pilot study in children with cerebral palsy. *Developmental Medicine & Child Neurology.* 2012;54(9):822-828.

24. Buccino G, Molinaro A, Ambrosi C, et al. Action Observation Treatment Improves Upper Limb Motor Functions in Children with Cerebral Palsy: A Combined Clinical and Brain Imaging Study. *Neural Plasticity.* 2018;2018:4843985.

25. Elbagoury WS, El-Saeed TM, Olama KA, Kamel MI. Functional-outcomes-of-verbaldirected-training-versus-visualdirected-training-in-children-with-unilateral-cerebral-palsy. *26.* 2022;1:1205-1210.

26. Kim DH, An DH, Yoo WG. Effects of live and video form action observation training on upper limb function in children with hemiparetic cerebral palsy. *Technology & Health Care.* 2018;26(3):437-443.

27. Kim DH. Comparison of short- and long-time action observation training (AOT) on upper limb function in children with cerebral palsy. *Physiotherapy Practice & Research.* 2020;41(1):53-58.

28. Kirkpatrick E, Pearse J, James P, Basu A. Effect of parent-delivered action observation therapy on upper limb function in unilateral cerebral palsy: a randomized controlled trial. *Developmental Medicine & Child Neurology.* 2016;58(10):1049-1056.

29. Palomo-Carrion R, Zuil-Escobar JC, Cabrera-Guerra M, Barreda-Martinez P, Martinez-Cepa CB. Mirror and action observation therapy in children with unilateral spastic cerebral palsy: a feasibility study. *Revista de Neurologia.* 2022;75(11):325-332.

30. Quadrelli E, Anzani A, Ferri M, et al. Electrophysiological correlates of action observation treatment in children with cerebral palsy: A pilot study. *Developmental Neurobiology.* 2019;79(11-12):934-948.

31. Sgandurra G, Ferrari A, Cossu G, Guzzetta A, Fogassi L, Cioni G. Randomized trial of observation and execution of upper extremity actions versus action alone in children with unilateral cerebral palsy. *Neurorehabilitation & Neural Repair.* 2013;27(9):808-815.

32. Araneda R, Ebner-Karestinos D, Paradis J, et al. Changes Induced by Early Hand-Arm Bimanual Intensive Therapy Including Lower Extremities in Young Children With Unilateral Cerebral Palsy: A Randomized Clinical Trial. *JAMA Pediatrics.* 2024;178(1):19-28.

33. Bleyenheuft Y, Arnould C, Brandao MB, Bleyenheuft C, Gordon AM. Hand and Arm Bimanual Intensive Therapy Including Lower Extremity (HABIT-ILE) in Children With Unilateral Spastic Cerebral Palsy: A Randomized Trial. *Neurorehabilitation & Neural Repair.* 2015;29(7):645-657.

34. Brandao MB, Ferre C, Kuo HC, et al. Comparison of Structured Skill and Unstructured Practice During Intensive Bimanual Training in Children With Unilateral Spastic Cerebral Palsy. *Neurorehabilitation & Neural Repair.* 2014;28(5):452-461.

35. Brandao MB, Mancini MC, Ferre CL, et al. Does Dosage Matter? A Pilot Study of Hand-Arm Bimanual Intensive Training (HABIT) Dose and Dosing Schedule in Children with Unilateral Cerebral Palsy. *Physical & Occupational Therapy in Pediatrics.* 2018;38(3):227-242.

36. Facchin P, Rosa-Rizzotto M, Pozza LVD, et al. Multisite Trial Comparing the Efficacy of Constraint-Induced Movement Therapy with that of Bimanual Intensive Training in Children with Hemiplegic Cerebral Palsy. *American Journal of Physical Medicine & Rehabilitation.* 2011;90(7):539-553.

37. Fedrizzi E, Rosa-Rizzotto M, Turconi AC, et al. Unimanual and bimanual intensive training in children with hemiplegic cerebral palsy and persistence in time of hand function improvement: 6-month follow-up results of a multisite clinical trial. *Journal of Child Neurology.* 2013;28(2):161-175.

38. Ferre CL, Brandao M, Surana B, Dew AP, Moreau NG, Gordon AM. Caregiver-directed home-based intensive bimanual training in young children with unilateral spastic cerebral palsy: a randomized trial. *Developmental Medicine & Child Neurology.* 2017;59(5):497-504.

39. Aarts PB, Jongerius PH, Geerdink YA, van Limbeek J, Geurts AC. Effectiveness of modified constraint-induced movement therapy in children with unilateral spastic cerebral palsy: a randomized controlled trial. *Neurorehabilitation & Neural Repair.* 2010;24(6):509-518.

40. Geerdink Y, Aarts P, Geurts AC. Motor learning curve and long-term effectiveness of modified constraint-induced movement therapy in children with unilateral cerebral palsy: a randomized controlled trial. *Research in Developmental Disabilities.* 2013;34(3):923-931.

41. Abd El-Kafy EM, Elshemy SA, Alghamdi MS. Effect of constraint-induced therapy on upper limb functions: a randomized control trial. *Scandinavian Journal of Occupational Therapy.* 2014;21(1):11-23.

42. Al-Oraibi S, Eliasson AC. Implementation of constraint-induced movement therapy for young children with unilateral cerebral palsy in Jordan: a home-based model. *Disability & Rehabilitation.* 2011;33(21-22):2006-2012.

43. de Brito Brandao M, Mancini MC, Vaz DV, Pereira de Melo AP, Fonseca ST. Adapted version of constraint-induced movement therapy promotes functioning in children with cerebral palsy: a randomized controlled trial. *Clinical Rehabilitation.* 2010;24(7):639-647.

44. Case-Smith J, DeLuca SC, Stevenson R, Ramey SL. Multicenter randomized controlled trial of pediatric constraint-induced movement therapy: 6-month follow-up. *American Journal of Occupational Therapy.* 2012;66(1):15-23.

45. DeLuca SC, Case-Smith J, Stevenson R, Ramey SL. Constraint-induced movement therapy (CIMT) for young children with cerebral palsy: effects of therapeutic dosage. *Journal of Pediatric Rehabilitation Medicine.* 2012;5(2):133-142.

46. Chamudot R, Parush S, Rigbi A, Horovitz R, Gross-Tsur V. Effectiveness of Modified Constraint-Induced Movement Therapy Compared With Bimanual Therapy Home Programs for Infants With Hemiplegia: A Randomized Controlled Trial. *American Journal of Occupational Therapy.* 2018;72(6):7206205010p7206205011-7206205010p7206205019.

47. Charles JR, Wolf SL, Schneider JA, Gordon AM. Efficacy of a child-friendly form of constraint-induced movement therapy in hemiplegic cerebral palsy: a randomized control trial. *Developmental Medicine & Child Neurology.* 2006;48(8):635-642.

48. Chen CL, Kang LJ, Hong WH, Chen FC, Chen HC, Wu CY. Effect of therapist-based constraint-induced therapy at home on motor control, motor performance and daily function in children with cerebral palsy: a randomized controlled study. *Clinical Rehabilitation.* 2013;27(3):236-245.

49. Chen HC, Chen CL, Kang LJ, Wu CY, Chen FC, Hong WH. Improvement of upper extremity motor control and function after home-based constraint induced therapy in children with unilateral cerebral palsy: immediate and long-term effects. *Archives of Physical Medicine & Rehabilitation.* 2014;95(8):1423-1432.

50. Hsin YJ, Chen FC, Lin KC, Kang LJ, Chen CL, Chen CY. Efficacy of Constraint-Induced Therapy on Functional Performance and Health-Related Quality of Life for Children With Cerebral Palsy: A Randomized Controlled Trial. *Journal of Child Neurology.* 2012;27(8):992-999.

51. Christmas PM, Sackley C, Feltham MG, Cummins C. A randomized controlled trial to compare two methods of constraint-induced movement therapy to improve functional ability in the affected upper limb in pre-school children with hemiplegic cerebral palsy: CATCH TRIAL. *Clinical Rehabilitation.* 2018;32(7):909-918.

52. Choudhary A, Gulati S, Kabra M, et al. Efficacy of modified constraint induced movement therapy in improving upper limb function in children with hemiplegic cerebral palsy: a randomized controlled trial. *Brain & Development.* 2013;35(9):870-876.

53. Deppe W, Thuemmler K, Fleischer J, Berger C, Meyer S, Wiedemann B. Modified constraint-induced movement therapy versus intensive bimanual training for children with hemiplegia - a randomized controlled trial. *Clinical Rehabilitation.* 2013;27(10):909-920.

54. Eliasson AC, Nordstrand L, Ek L, et al. The effectiveness of Baby-CIMT in infants younger than 12 months with clinical signs of unilateral-cerebral palsy; an explorative study with randomized design. *Research in Developmental Disabilities.* 2018;72:191-201.

55. Eugster-Buesch F, de Bruin ED, Boltshauser E, et al. Forced-use therapy for children with cerebral palsy in the community setting: a single-blinded randomized controlled pilot trial. *Journal of Pediatric Rehabilitation Medicine.* 2012;5(2):65-74.

56. Gelkop N, Burshtein DG, Lahav A, et al. Efficacy of constraint-induced movement therapy and bimanual training in children with hemiplegic cerebral palsy in an educational setting. *Physical & Occupational Therapy in Pediatrics.* 2015;35(1):24-39.

57. Gordon AM, Hung YC, Brandao M, et al. Bimanual training and constraint-induced movement therapy in children with hemiplegic cerebral palsy: a randomized trial. *Neurorehabilitation & Neural Repair.* 2011;25(8):692-702.

58. de Brito Brandao M, Gordon AM, Mancini MC. Functional impact of constraint therapy and bimanual training in children with cerebral palsy: a randomized controlled trial. *American Journal of Occupational Therapy.* 2012;66(6):672-681.

59. Hoare B, Imms C, Villanueva E, Rawicki HB, Matyas T, Carey L. Intensive therapy following upper limb botulinum toxin A injection in young children with unilateral cerebral palsy: a randomized trial. *Developmental Medicine & Child Neurology.* 2013;55(3):238-247.

60. Hwang YS, Kwon JY. Effects of Modified Constraint-Induced Movement Therapy in Real-World Arm Use in Young Children with Unilateral Cerebral Palsy: A Single-Blind Randomized Trial. *Neuropediatrics.* 2020;51(4):259-266.

61. Kirton A, Andersen J, Herrero M, et al. Brain stimulation and constraint for perinatal stroke hemiparesis: The PLASTIC CHAMPS Trial. *Neurology.* 2016;86(18):1659-1667.

62. Kuo HC, Zewdie E, Ciechanski P, Damji O, Kirton A. Intervention-Induced Motor Cortex Plasticity in Hemiparetic Children With Perinatal Stroke. *Neurorehabil Neural Repair.* 2018;32(11):941-952.

63. Liang KJ, Chen HL, Huang CW, Wang TN. Efficacy of Constraint-Induced Movement Therapy Versus Bimanual Intensive Training on Motor and Psychosocial Outcomes in Children With Unilateral Cerebral Palsy: A Randomized Trial. *Am J Occup Ther.* 2023;77(4).

64. Lin KC, Wang TN, Wu CY, et al. Effects of home-based constraint-induced therapy versus dose-matched control intervention on functional outcomes and caregiver well-being in children with cerebral palsy. *Research in Developmental Disabilities.* 2011;32(5):1483-1491.

65. Maitre NL, Jeanvoine A, Yoder PJ, et al. Kinematic and Somatosensory Gains in Infants with Cerebral Palsy After a Multi-Component Upper-Extremity Intervention: A Randomized Controlled Trial. *Brain Topography.* 2020.

66. Maring J, Wentzell E. Constraint Induced Movement Therapy: Impact of Setting on Outcomes. *Journal of Allied Health.* 2019;48(3):e73-e77.

67. Ostadzadeh A, Amini M, Hassani Mehraban A, Maroufizadeh S, Farajzadeh A. The Effect of Occupation-Based Modified Constraint-Induced Movement Therapy on the Participation of Children with Cerebral Palsy: A Single-Blind Randomized Controlled Trial. *Iran J Child Neurol.* 2023;17(2):39-54.

68. Palomo-Carrion R, Pinero-Pinto E, Ando-LaFuente S, Ferri-Morales A, Bravo-Esteban E, Romay-Barrero H. Unimanual Intensive Therapy with or without Unaffected Hand Containment in Children with Hemiplegia. A Randomized Controlled Pilot Study. *Journal of Clinical Medicine.* 2020;9(9):14.

69. Palomo-Carrion R, Lirio-Romero C, Ferri-Morales A, Jovellar-Isiegas P, Cortes-Vega MD, Romay-Barrero H. Combined intensive therapies at home in spastic unilateral cerebral palsy with high bimanual functional performance. What do they offer? A comparative randomised clinical trial. *Therapeutic Advances in Chronic Disease.* 2021;12:20406223211034996.

70. Ramey SL, DeLuca SC, Stevenson RD, Conaway M, Darragh AR, Lo W. Constraint-induced movement therapy for cerebral palsy: A randomized trial. *Pediatrics.* 2021;148(5).

71. Rostami HR, Malamiri RA. Effect of treatment environment on modified constraint-induced movement therapy results in children with spastic hemiplegic cerebral palsy: a randomized controlled trial. *Disability & Rehabilitation.* 2012;34(1):40-44.

72. Sakzewski L, Ziviani J, Abbott DF, Macdonell RAL, Jackson GD, Boyd RN. Randomized trial of constraint-induced movement therapy and bimanual training on activity outcomes for children with congenital hemiplegia. *Developmental Medicine and Child Neurology.* 2011;53(4):313-320.

73. Sakzewski L, Ziviani J, Abbott DF, Macdonell RA, Jackson GD, Boyd RN. Equivalent retention of gains at 1 year after training with constraint-induced or bimanual therapy in children with unilateral cerebral palsy. *Neurorehabilitation & Neural Repair.* 2011;25(7):664-671.

74. Sakzewski L, Ziviani J, Abbott DF, Macdonell RA, Jackson GD, Boyd RN. Participation Outcomes in a Randomized Trial of 2 Models of Upper-Limb Rehabilitation for Children With Congenital Hemiplegia. *Archives of Physical Medicine & Rehabilitation.* 2011;92(4):531-539.

75. Sakzewski L, Provan K, Ziviani J, Boyd RN. Comparison of dosage of intensive upper limb therapy for children with unilateral cerebral palsy: how big should the therapy pill be? *Research in Developmental Disabilities.* 2015;37:9-16.

76. Sakzewski L, Miller L, Ziviani J, et al. Randomized comparison trial of density and context of upper limb intensive group versus individualized occupational therapy for children with unilateral cerebral palsy. *Developmental Medicine & Child Neurology.* 2015;57(6):539-547.

77. Smania N, Aglioti SM, Cosentino A, et al. A modified constraint-induced movement therapy (CIT) program improves paretic arm use and function in children with cerebral palsy. *European journal of physical & rehabilitation medicine.* 2009;45(4):493-500.

78. Sung IY, Ryu JS, Pyun SB, Yoo SD, Song WH, Park MJ. Efficacy of forced-use therapy in hemiplegic cerebral palsy. *Archives of Physical Medicine & Rehabilitation.* 2005;86(11):2195-2198.

79. Taub E, Ramey SL, DeLuca S, Echols K. Efficacy of constraint-induced movement therapy for children with cerebral palsy with asymmetric motor impairment. *Pediatrics.* 2004;113(2):305-312.

80. Deluca SC, Echols K, Law CR, Ramey SL. Intensive pediatric constraint-induced therapy for children with cerebral palsy: randomized, controlled, crossover trial. *Journal of Child Neurology.* 2006;21(11):931-938.

81. Taub E, Griffin A, Uswatte G, Gammons K, Nick J, Law CR. Treatment of Congenital Hemiparesis With Pediatric Constraint-Induced Movement Therapy. *Journal of Child Neurology.* 2011;26(9):1163-1173.

82. Vaghela VG. To Study the effects of Mcimt Versus Cimt for Young Children with Spastic Hemiplegic Cerebral Palsy-- A Comparitive Study. *Indian Journal of Physiotherapy & Occupational Therapy.* 2014;8(2):136-141.

83. Wallen M, Ziviani J, Naylor O, Evans R, Novak I, Herbert RD. Modified constraint-induced therapy for children with hemiplegic cerebral palsy: a randomized trial. *Developmental Medicine & Child Neurology.* 2011;53(12):1091-1099.

84. Xu K, Wang L, Mai J, He L. Efficacy of constraint-induced movement therapy and electrical stimulation on hand function of children with hemiplegic cerebral palsy: a controlled clinical trial. *Disability & Rehabilitation.* 2012;34(4):337-346.

85. Yu J, Kang H, Jung J. Effects of modified constraint-induced movement therapy on hand dexterity, grip strength and activities of daily living of children with cerebral palsy: a randomized control trial. *Journal of physical therapy science.* 2012;24(10):1029‐1031.

86. Zafer H, Amjad I, Malik AN, Shaukat E. Effectiveness of constraint induced movement therapy as compared to bimanual therapy in upper motor function outcome in child with hemiplegic cerebral palsy. *Pakistan Journal of Medical Sciences.* 2016;32(1):181-184.

87. Bruchez R, Gygax MJ, Roches S, et al. Mirror therapy in children with hemiparesis: a randomized observer-blinded trial. *Developmental Medicine and Child Neurology.* 2016;58(9):970-978.

88. Elsepaee MI, Elhadidy EI, Emara HA, Nawar EAE. EFFECT OF MIRROR VISUAL FEEDBACK ON HAND FUNCTIONS IN CHILDREN WITH HEMIPARESIS. *International Journal of Physiotherapy.* 2016;3(2):147-153.

89. Kara OK, Yardimci BN, Sahin S, Orhan C, Livanelioglu A, Soylu AR. Combined Effects of Mirror Therapy and Exercises on the Upper Extremities in Children with Unilateral Cerebral Palsy: A Randomized Controlled Trial. *Developmental neurorehabilitation.* 2020;23(4):253-264.

90. Narimani A, Kalantari M, Dalvand H, Tabatabaee SM. Effect of mirror therapy on dexterity and hand grasp in children aged 9-14 years with hemiplegic cerebral palsy. *Iranian Journal of Child Neurology.* 2019;13(4):135-142.

91. Cameron D, Craig T, Edwards B, Missiuna C, Schwellnus H, Polatajko HJ. Cognitive Orientation to daily Occupational Performance (CO-OP): A New Approach for Children with Cerebral Palsy. *Phys Occup Ther Pediatr.* 2017;37(2):183-198.

92. Holmström L, Eliasson AC, Almeida R, et al. Efficacy of the small step program in a randomized controlled trial for infants under 12 months old at risk of cerebral palsy (CP) and other neurological disorders. *Journal of Clinical Medicine.* 2019;8(7).

93. Jackman M, Novak I, Lannin N, Froude E, Miller L, Galea C. Effectiveness of Cognitive Orientation to daily Occupational Performance over and above functional hand splints for children with cerebral palsy or brain injury: a randomized controlled trial. *BMC Pediatrics.* 2018;18(1):248.

94. Ko EJ, Sung IY, Moon HJ, Yuk JS, Kim H-S, Lee NH. Effect of Group-Task-Oriented Training on Gross and Fine Motor Function, and Activities of Daily Living in Children with Spastic Cerebral Palsy. *Physical & Occupational Therapy in Pediatrics.* 2020;40(1):18-30.

95. Law MC, Darrah J, Pollock N, et al. Focus on function: a cluster, randomized controlled trial comparing child- versus context-focused intervention for young children with cerebral palsy. *Developmental Medicine & Child Neurology.* 2011;53(7):621-629.

96. Moon J-H, Jung J-H, Hahm S-C, Cho H-y. The effects of task-oriented training on hand dexterity and strength in children with spastic hemiplegic cerebral palsy: A preliminary study. *Journal of physical therapy science.* 2017;29(10):1800-1802.

97. Sousa LK, Brandao MB, Curtin CM, Magalhaes LC. A Collaborative and Cognitive-based Intervention for Young People with Cerebral Palsy. *Canadian Journal of Occupational Therapy - Revue Canadienne d Ergotherapie.* 2020;87(4):319-330.

98. Yuan A, Hou M, Wang S, Liu Q, Li Y, Chen JI. Goals-activity-motor enrichment can improve the motor functioning of infants with a mild to moderate developmental disorder *Chinese Journal of Physical Medicine and Rehabilitation* 2023;12:808-812.

99. Wallen M, O'Flaherty SJ, Waugh MC. Functional outcomes of intramuscular botulinum toxin type a and occupational therapy in the upper limbs of children with cerebral palsy: a randomized controlled trial. *Archives of Physical Medicine & Rehabilitation.* 2007;88(1):1-10.

100. Friel KM, Ferre CL, Brandao M, et al. Improvements in Upper Extremity Function Following Intensive Training Are Independent of Corticospinal Tract Organization in Children With Unilateral Spastic Cerebral Palsy: A Clinical Randomized Trial. *Frontiers in neurology [electronic resource].* 2021;12:660780.

101. Afzal MT, Amjad I, Ghous M. Comparison of classic constraint-induced movement therapy and its modified form on upper extremity motor functions and psychosocial impact in hemiplegic cerebral palsy. *Journal of the Pakistan Medical Association.* 2022;72(7):1418-1421.

102. Bingol H, Gunel MK. Comparing the effects of modified constraint-induced movement therapy and bimanual training in children with hemiplegic cerebral palsy mainstreamed in regular school: A randomized controlled study. *Arch Pediatr.* 2022;29(2):105-115.

103. Dong AQ, Fong NK. Remind to move - A novel treatment on hemiplegic arm functions in children with unilateral cerebral palsy: A randomized cross-over study. *Developmental neurorehabilitation.* 2016;19(5):275-283.

104. Mohamed RA, Yousef AM, Radwan NL, Ibrahim MM. Efficacy of different approaches on quality of upper extremity function, dexterity and grip strength in hemiplegic children: a randomized controlled study. *European Review for Medical & Pharmacological Sciences.* 2021;25(17):5412-5423.

105. Rostami HR, Arastoo AA, Nejad SJ, Mahany MK, Malamiri RA, Goharpey S. Effects of modified constraint-induced movement therapy in virtual environment on upper-limb function in children with spastic hemiparetic cerebral palsy: a randomised controlled trial. *Neurorehabilitation.* 2012;31(4):357-365.
