# Supplementary figures and images for: Active upper‐limb therapies for hand function, individual goal achievement, and self‐care in children with cerebral palsy: A network meta‐analysis
Source: Dev Med Child Neurol. 2025 Sep 5;67(12):1543–53. doi: 10.1111/dmcn.16476 (PMC12618955; doi:10.1111/dmcn.16476)

## Figure S1 PRISMA Flowchart


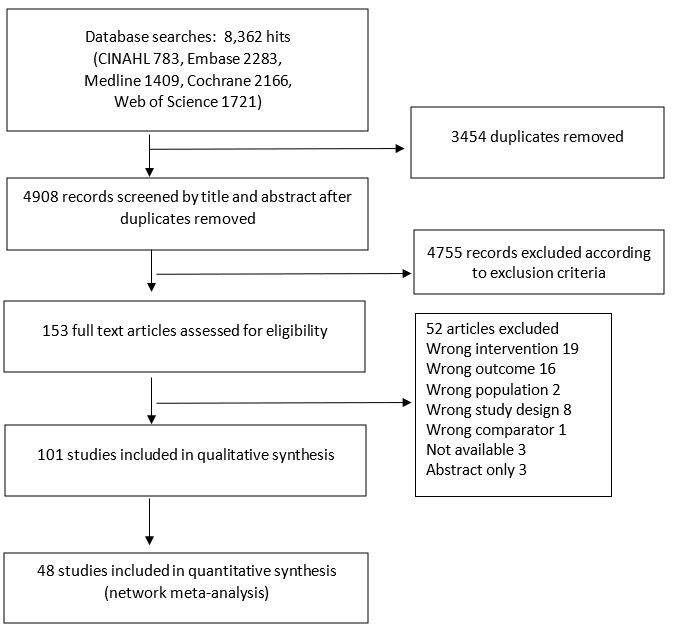

Supplement: Supplementary file 11 — Figure S1: PRISMA flowchart [file DMCN-67-1543-s001.docx]
